# Base metal-catalyzed benzylic oxidation of (aryl)(heteroaryl)methanes with molecular oxygen

**DOI:** 10.3762/bjoc.12.16

**Published:** 2016-01-27

**Authors:** Hans Sterckx, Johan De Houwer, Carl Mensch, Wouter Herrebout, Kourosch Abbaspour Tehrani, Bert U W Maes

**Affiliations:** 1 Department of Chemistry, University of Antwerp, Groenenborgerlaan 171, B-2020 Antwerp, Belgium

**Keywords:** base metal, benzylic, catalyzed, molecular oxygen, oxygenation

## Abstract

The methylene group of various substituted 2- and 4-benzylpyridines, benzyldiazines and benzyl(iso)quinolines was successfully oxidized to the corresponding benzylic ketones using a copper or iron catalyst and molecular oxygen as the stoichiometric oxidant. Application of the protocol in API synthesis is exemplified by the alternative synthesis of a precursor to the antimalarial drug Mefloquine. The oxidation method can also be used to prepare metabolites of APIs which is illustrated for the natural product papaverine. ICP–MS analysis of the purified reaction products revealed that the base metal impurity was well below the regulatory limit.

## Introduction

Direct oxidation of C(sp^3^)–H bonds is a useful and fast method to convert fairly unreactive substrates to useful functional groups for organic synthesis like alcohols, ketones, aldehydes and carboxylic acids. Classical oxidation protocols rely on the use of (super)stoichiometric quantities of oxyanions of toxic metals like Mn(VII) and Cr(VI) [1–2]. The amount of waste these oxidants produce and limitations on their use by new legislation [3] has prompted scientists to search for more sustainable oxidation methods. The use of transition metal- or organocatalysis in combination with molecular oxygen has received a great deal of attention from the scientific community [4–7]. Molecular oxygen is considered to be the greenest oxidant available and it is already widely employed by the commodity chemical industry [8]. However, when looking at the preparation of more complex molecules, typical for fine chemicals, the use of aerobic oxidations is more the exception than the norm [9]. This is partly due to the limited synthetic scope and selectivity of the available oxidation methods. Further research into selective and mild aerobic oxidations is therefore of vital importance. Of special interest are the transition metal- and organocatalyzed oxidations of activated methylenes such as in benzylic methylenes or their heteroaromatic analogues. Due to the activation, the formation of the corresponding ketones and aldehydes becomes feasible under mild conditions. Oxidations of this kind using Oxone^®^ [10–11], NaOCl [12] or especially peroxides [13–19] as the terminal oxidant are quite numerous. However, transformations using molecular oxygen are rare. Ishii showed that organocatalysts such as *N*-hydroxyphthalimide (NHPI) in combination with molecular oxygen can be used to perform benzylic oxidations [20]. The aerobic copper-catalyzed α-oxygenation of 2-arylthioacetamides was reported by Moghaddam [21]. In this transformation CuCl_2_ and K_2_CO_3_ in DMF were used to produce α-ketoarylthioacetamides. The coupling of 2-arylacetaldehydes with anilines resulting in the formation of 2-aryl-α-ketoacetamides was reported by Jiao [22] and a closely related intramolecular variant leading to isatins has been published by Ilangovan [23]. A remarkable Cu-catalyzed chemoselective oxidative C–C bond cleavage of methyl ketones was reported by the group of Liu and Bi [24]. This useful transformation makes use of CuI/O_2_ in DMSO to convert methyl ketones into aldehydes in a sustainable manner.

Recently our group reported a synthetic protocol for the copper- and iron-catalyzed aerobic oxidation of the methylene group of aryl(di)azinylmethanes using acetic acid as a promotor [25]. The resulting ketones are very valuable as they are intermediates in the synthesis of a variety of pharmaceuticals such as the antimalarial Mefloquine (Lariam^®^), the antihistamine Acrivastine, the β_2_-adrenergic agonist Rimiterol and the anxiolytic Bromazepam [26]. Furthermore, they can also be used to synthesize the 1st and 2nd generation antihistamines Carbinoxamine, Bepotastine and Triprolidine through an alternative synthetic route. In addition to these synthetic examples it has been shown by Kamijo that 4-benzoylpyridines can act as efficient organocatalysts in the photoinduced oxidation of secondary alcohols [27]. Very recently the group of Zhuo and Lei disclosed an alteration to our reaction conditions to further extend the substrate scope [28]. Ethyl chloroacetate was used as the promotor instead of acetic acid, allowing the authors to additionally oxidize less reactive alkyl-substituted pyridines. Gao showed that NH_4_I can also be used as an organocatalyst in combination with AcOH to facilitate the oxidation of benzylpyridines to benzoylpyridines [29]. Satoh and Miura showed that when replacing O_2_ for Na_2_S_2_O_8_ chemoselective methylenation occurred over oxygenation of the methylene with DMA acting as a one-carbon source [30]. An alternative method to synthesize picolinic amides from picolines and ammonium acetate or amines using a similar oxidation protocol was simultaneously proposed by the groups of Deng and Yin [31–32]. In the current work we study the expansion of the scope of our previously disclosed method and provide specific examples of applications in organic synthesis.

## Results and Discussion

### Substrate scope

In our communication we provided a reaction scope of phenyl-substituted 2-benzylpyridines and showed that both, electron-withdrawing and donating groups are well tolerated. The results additionally indicated that either Cu and Fe catalysts (CuI and FeCl_2_·4H_2_O) worked equally well for this substrate class [25]. In the framework of this work a similar study was executed for the regioisomeric 4-benzylpyridines using FeCl_2_·4H_2_O as the catalyst. Under the standard conditions previously developed for 2-benzylpyridines these substrates smoothly oxidized giving the corresponding ketones in moderate to good yields (Table 1). Also in this case electron-donating as well as electron-withdrawing substituents on the phenyl ring are well tolerated and their electronic properties have little influence on the yield of the reaction. Even substituents that are sensitive to oxidation such as NH_2_ (**2b**) and SMe (**2c**) appear to be no problem although the reaction products were isolated in slightly lower yields.

**Table 1:** Iron-catalyzed aerobic oxidation of phenyl-substituted 4-benzylpyridines (**1**).^a^

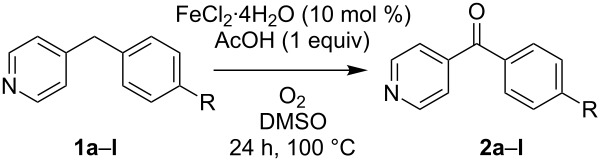				

Entry	Substrate	R	Product	Yield (%)^b^

1	**1a**	H	**2a**	70
2	**1b**	NH_2_	**2b**	55
3	**1c**	SMe	**2c**	56
4	**1d**	OMe	**2d**	67
5	**1e**	Me	**2e**	79
6	**1f**	I	**2f**	77
7	**1g**	Br	**2g**	85
8	**1h**	Cl	**2h**	66
9	**1i**	F	**2i**	76
10	**1j**	CO_2_Et	**2j**	61
11	**1k**	CN	**2k**	79
12	**1l**	NO_2_	**2l**	60

^a^Reactions were performed on a 0.5 mmol scale in 1 mL of solvent using 1 atmosphere of O_2_ (balloon). ^b^Isolated yields.

Next, pyridine rather than phenyl-substitution was studied. In contrast to the phenyl-substituted compounds, substitution on the pyridine ring exerted a large influence on the rate of the reaction (Table 2). This is not surprising when considering the mechanism of the reaction involving an initial acid catalyzed imine–enamine tautomerization (the calculation of the equilibrium constants can be found in Supporting Information File 1, Table S1) [33]. As the substituent is now located in the ring where the tautomerization will take place the electronic effect and the position of this substituent is expected to have a large effect on it. In general one expects the tautomerization to proceed more efficiently when the pyridine nitrogen becomes more basic and the methylene hydrogen becomes more acidic. In Table 2 the results on pyridine-substituted 2-benzylpyridines using both Fe and Cu catalysis are shown. Under the standard conditions only a small number of substrates reached full conversion after 24 hours. Based on our findings that placing substituents on the phenyl ring, both in 2- and 4-benzylpyridines and irrespective of their electronic nature, has little influence on the yield of the reaction the largest substituent effect is expected to be on the basicity of the pyridine nitrogen and not that much on the acidity of the methylene hydrogen.

**Table 2:** Iron and copper-catalyzed aerobic oxidation of pyridine-substituted 2-benzylpyridines (**3**).^a^

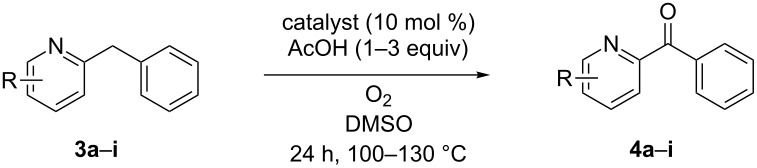						

Entry	Catalyst	Substrate	R	Product	Yield **3** (%)^b^	Yield **4** (%)^b^

1	FeCl_2_·4H_2_O	**3a**	5-CN	**4a**	19	67
2	CuI	**3a**	5-CN	**4a**	18	66
3	CuI	**3a**	5-CN^c^	**4a**	0	83
4	FeCl_2_·4H_2_O	**3b**	5-Me	**4b**	9	73
5	CuI	**3b**	5-Me	**4b**	9	82
6	CuI	**3b**	5-Me^c^	**4b**	0	72
7	FeCl_2_·4H_2_O	**3c**	5-OMe	**4c**	65	15
8	CuI	**3c**	5-OMe	**4c**	66	15
9	CuI	**3c**	5-OMe^d,e^	**4c**	0	65
10	FeCl_2_·4H_2_O	**3d**	5-CO_2_Me	**4d**	0	69
11	CuI	**3d**	5-CO_2_Me	**4d**	0	62
12	FeCl_2_·4H_2_O	**3e**	5-NHCOMe	**4e**	0	64
13	CuI	**3e**	5-NHCOMe	**4e**	27	56
14	CuI	**3e**	5-NHCOMe^c^	**4e**	0	91
15	FeCl_2_·4H_2_O	**3f**	4-Cl	**4f**	0	85
16	CuI	**3f**	4-Cl	**4f**	8	88
17	FeCl_2_·4H_2_O	**3g**	3-Cl	**4g**	73	23
18	CuI	**3g**	3-Cl	**4g**	71	22
19	CuI	**3g**	3-Cl^d,e^	**4g**	0	87
20	FeCl_2_·4H_2_O	**3h**	5-Cl	**4h**	70	20
21	CuI	**3h**	5-Cl	**4h**	69	15
22	CuI	**3h**	5-Cl^d,e^	**4h**	0	92
23	FeCl_2_·4H_2_O	**3i**	6-Cl	**4i**	90	0
24	CuI	**3i**	6-Cl	**4i**	91	0
25	CuI	**3i**	6-Cl^e, f^	**4i**	0	59

^a^Reactions were performed on a 0.5 mmol scale in 1 mL of solvent using 1 atmosphere of O_2_ (balloon). ^b^Isolated yields. ^c^48 h. ^d^AcOH (3 equiv). ^e^130 °C. ^f^TFA (3 equiv).

**Figure 1:**
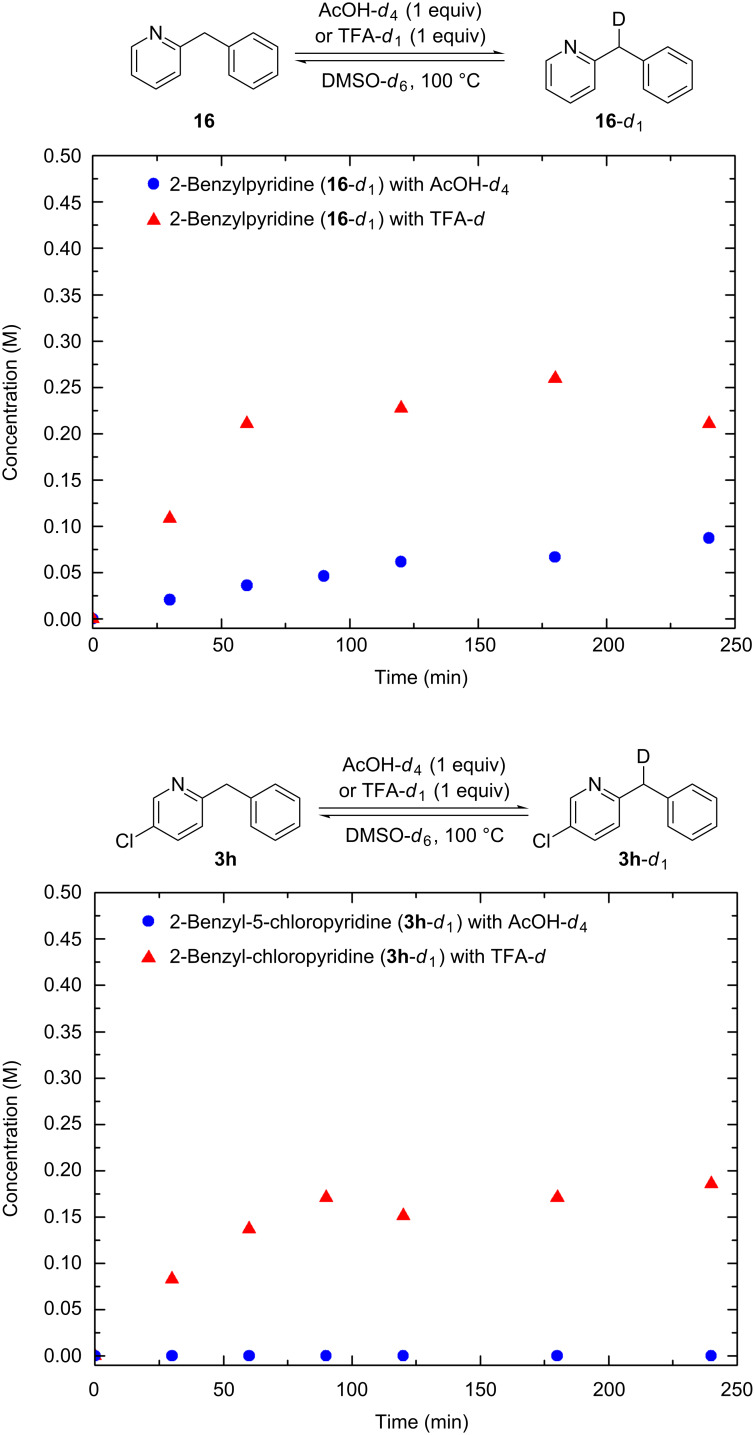
Hydrogen–deuterium exchange through acid-catalyzed imine–enamine tautomerization of **3h** (0.5 M) and **16** (0.5 M) using AcOH-*d*_4_ (0.5 M) or TFA-*d*_1_ (0.5 M). ^1^H NMR spectroscopy was used to quantify the monodeuterated species.

While the thermodynamical equilibrium constant between the imine and enamine tautomers predicts whether or not a substrate can be oxidized (see Supporting Information File 1), it does not provide an explanation for the incomplete conversion that is seen in most cases of the pyridine-substituted 2-benzylpyridines. We attribute the low conversions to the fact that the pyridine nitrogen becomes less basic and therefore protonation by AcOH becomes unfavored. This is supported by the fact that the rate of deuterium incorporation in the benzylic position of **16** and **3h** through acid-catalyzed imine–enamine tautomerization is dependent on the strength of the acid used (Figure 1). The rate of deuterium incorporation in 2-benzylpyridine (**16**, p*K*_a_ ≈ 5.2) is much faster when using TFA-*d*_1_ than with AcOH-*d*_4_ [34–35]. With the less basic 2-benzyl-5-chloropyridine (**3h**, p*K*_a_ ≈ 3.0) the difference is even more pronounced: Almost no deuterium incorporation could be detected using AcOH-*d*_4_ while the reaction ran smoothly using the stronger acid TFA-*d*_1_. From this we conclude that when the pyridine nitrogen becomes less basic and protonation by the acid thus becomes more difficult, using more equivalents of the acid or a stronger acid is needed to reach full conversion. When we compare the different p*K*_a_ values of substituted pyridines we see that 2-benzylpyridine (**16**, p*K*_a_ ≈ 5.2) is one of the most basic pyridines [34–35]. Substituents in the 5-position generally give poor conversion in accordance with their lower p*K*a values: 5-CN (p*K*_a_ ≈ 1.3, Table 2, entries 1 and 2), 5-OMe (p*K*_a_ ≈ 4.9, Table 2, entries 7 and 8) and 5-Cl (p*K*_a_ ≈ 3.0, Table 2, entries 20 and 21) with the exception of 5-CO_2_Me (p*K*_a_ ≈ 3.1, Table 2, entries 10 and 11). When investigating regioisomeric substrates featuring chloro substituents in all possible positions of the pyridine ring only the 4-Cl (p*K*_a_ ≈ 3.8, Table 2, entries 15 and 16) substrate reaches full conversion under the standard conditions. The 3-Cl and 5-Cl regioisomers have a similar p*K*_a_ value (p*K*_a_ ≈ 2.98) and therefore show similar reactivity (Table 2, entries 17 and 18 and entries 20 and 21) with only limited conversion. A remarkable case is the 6-Cl-substituted substrate (p*K*_a_ ≈ 0.8, Table 2, entries 23 and 24), which did not react at all under the standard conditions. The low basicity of the pyridine nitrogen is the reason for this lack of reactivity. In addition the enamine form of this compound is also highly unfavored (see Supporting Information File 1).

To reach full conversion for all substrates we re-optimized the reaction conditions. Although similar results for Fe and Cu catalysis were obtained after 24 h, CuI was selected for this purpose. The reasoning behind this is reflected in the chemoselectivity experiments (vide infra), where it was shown that CuI is a slightly more potent catalyst than FeCl_2_·4H_2_O. Additionally, comparison of the reaction rate for both catalysts on the standard substrate 2-benzylpyridine (**16**) further supports this (v_i,Cu_ = 1.088 × 10^−3^ M min^−1^; v_i,Fe_ = 1.013 × 10^−3^ M min^−1^, see Supporting Information File 1, Figure S2). For substrates that already gave reasonable conversions (>60%) using the standard conditions (Table 2, entries 3, 6 and 14) the reaction time was doubled to 48 hours which was sufficient to achieve full conversion. For substrates that are harder to oxidize (<60% conversion) due to too low p*K*_a_ a combination of a higher temperature and the addition of more (three) equivalents of acetic acid was needed (Table 2, entries 9, 19 and 22). Considering the low basicity of compound **3i**, 3 equivalents of the stronger acid TFA were used (Table 2, entry 25).

**Scheme 1:**
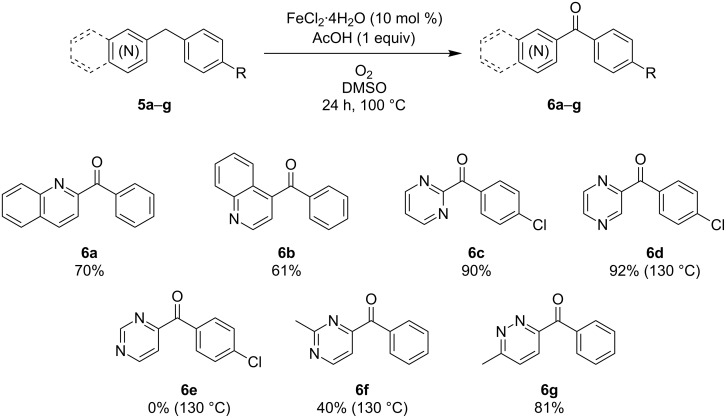
Benzylic oxygenation of benzoannulated azines and diazines (**5**).

Next, the effect of benzoannulation (quinoline) and C–H for N substitution (diazines) in the pyridine ring was studied. Scheme 1 provides an overview of the results for these more challenging substrates. Interestingly, phenyl(quinolin-2-yl)methanone (**6a**) and phenyl(quinolin-4-yl)methanone (**6b**) were formed in moderate yields indicating that larger aromatic systems are compatible with the reaction conditions. In the case of 2-(4-chlorobenzyl)pyrimidine (**5c**) the standard conditions allowed smooth oxidation providing the target compound in an excellent yield. For the regioisomeric diazine, (4-chlorobenzyl)pyrazine (**5d**), no oxidation was observed after 24 h at 100 °C. To our delight, by increasing the reaction temperature to 130 °C, (4-chlorophenyl)(pyrazin-2-yl)methanone (**6d**) could be obtained in 92% yield. In contrast to the two former cases, 4-(4-chlorobenzyl)pyrimidine (**5e**) could neither be oxidized at 100 °C nor at 130 °C. Competitive C–H activation of the 2 position is presumed to be the reason for this observation. Blocking this position by a methyl group (**5f**) delivered the corresponding ketone **6f**, albeit in a poor yield of 40%, thus supporting the metalation hypothesis. The regioisomer of diazine **5f**, 3-benzyl-6-methylpyridazine (**5g**), could be smoothly oxidized to the corresponding ketone in 81% yield. It is worth mentioning that in **5f** as well as **5g** no additional oxidation of the methyl group was seen [25].

### Applications

**Scheme 2:**
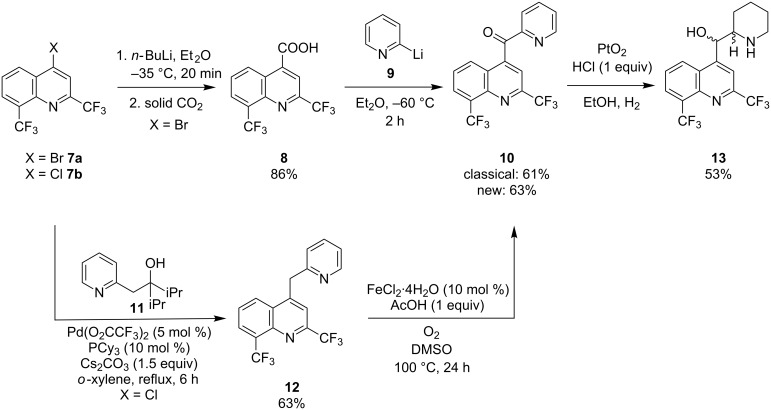
Classical (top) and new formal (bottom) synthesis of Mefloquine.

An example of an important pharmaceutical which is industrially prepared from an azinyl benzoazinyl ketone, namely (2,8-bis(trifluoromethyl)quinolin-4-yl)(pyridin-2-yl)methanone (**10**), is the antimalarial Mefloquine (**13**) [36]. This drug is listed on the World Health Organization essential medicines list and despite numerous side effects it remains one of the most effective antimalarial drugs on the market [37–38]. Its classical synthesis (Scheme 2, top) is based on the lithiation of 4-bromo-2,8-bis(trifluoromethyl)quinoline (**7a**) and quenching with CO_2_ resulting in the formation of 2,8-bis(trifluoromethyl)quinoline-4-carboxylic acid (**8**). Reaction of **8** with in situ generated 2-pyridyllithium (**9**) finally yields ketone **10** [39].

We considered a new approach based on 4-(pyridin-2-ylmethyl)-2,8-bis(trifluoromethyl)quinoline (**12**) as the substrate. This compound is structurally interesting as it is activated by both a pyridin-2-yl and a quinolin-4-yl moiety (Scheme 2, bottom). The synthesis of substrate **12** was accomplished by a cross-coupling reaction of pyridine alcohol **11** with commercial 4-chloro-2,8-bis(trifluoromethyl)quinoline (**7b**) according to a procedure published by Oshima [40]. In this way, **12** was obtained in 63% yield and its subsequent Fe-catalyzed oxidation provided **10** in 63% isolated yield. In principle substrate **7a** could also be used but the chloro analogue is cheaper. The final reduction of **10** into Mefloquine has been described earlier and can also be achieved in an enantioselective manner [41].

**Scheme 3:**
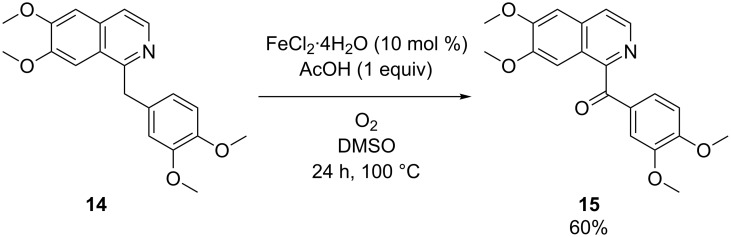
Iron-catalyzed aerobic oxidation of papaverine (**15**).

Human metabolism can produce metabolites of pharmaceuticals that possess completely different properties such as for instance biological activity, toxicity and clearance rates. Rapid identification and synthesis of potential drug metabolites is therefore of great importance to facilitate the drug discovery process [42]. For this purpose chemoselective oxidation protocols are a valuable tool since they can provide us with metabolites typically generated by cytochrome P450 enzymes. Bearing this in mind we attempted to oxygenate the benzylic position of the antispasmodic drug papaverine (**14**) applying our oxidation protocol (Scheme 3). The resulting compound is known as papaveraldine (**15**), a byproduct from the extraction of papaverine from *Papaver somniferum*. Papaverine is a challenging substrate as besides the methylene part it also features two oxidation sensitive veratrole units. Interestingly, a highly chemoselective oxidation was observed and compound **15** was isolated in 60% yield.

### Trace metal analysis

When using transition metal catalysis in the synthesis of compounds designated for application (pharmaceuticals, agrochemicals, materials) it is of vital importance to control and determine any metal impurity in the reaction product. In case the selected purification methods proved insufficient to get the metal contaminations below the maximum allowed threshold value set for active ingredients (AIs), an extra treatment might be required. This is especially true if the catalysis is performed in a late stage of a synthesis thus making the method less attractive and sustainable. While class 1 metals such as Pd and Pt have oral exposure limits of 10 ppm, Cu and Fe are respectively class 2 and class 3 metals with oral exposure limits of 250 ppm and 1300 ppm [43]. The fact that Cu and Fe are more abundant, cheaper and benign makes them an interesting choice as transition metal catalysts. To determine the Cu and Fe contents present in our samples ICP–MS analysis was performed. Copper contents ranged from 5 to 21 ppm, all well below the limit of 250 ppm. Iron contents were slightly higher ranging from 29 to 57 ppm but again still well below the regulatory maximum value (see Supporting Information File 1, Figure S1). It should be noted that these values pertain to the purified compounds after column chromatography. The influence of the work-up procedure on the remaining metal impurities was investigated for one of our applications, namely the papaveraldine (**15**) synthesis (Table 3). Applying the standard purification resulted in 54 ppm of Fe remaining. Omitting the column chromatography step and solely performing the aqueous extraction provided a much higher value, namely 1097 ppm. However, if after the extraction a recrystallization step of the reaction product is performed the Fe level can be lowered further to 300 ppm which is well below the legal limit for oral exposure (see Supporting Information File 1).

**Table 3:** The influence of the purification method on the amount of Fe impurities in papaveraldine (**15**) after oxidation.

Entry	Purification method	Fe impurity in **15** (ppm)

1	Extraction^a^ + column chromatography^b^	54
2	Extraction^a^	1097
3	Extraction^a^ + recrystallization^c^	300

^a^Washing subsequently with sat. aq NaHCO_3_ and brine, extraction with dichloromethane. ^b^Silica flash cartridge applying a heptane/ethyl acetate gradient. ^c^Recrystallization from a 2 M HCl solution.

### Alternative solvents

A solvent screening was subsequently performed focusing mainly on greener solvents than DMSO with a boiling point above 100 °C in order to allow a direct comparison at the same reaction temperature [44–45] (Table 4). All reactions were performed in a round-bottomed flask equipped with a reflux condenser under oxygen atmosphere at a 5 mmol scale to be able to reliably quantify remaining starting material. 2-Benzylpyridine (**16**) was selected as the substrate for this study. The reaction in anisole and *n-*BuOAc gave full conversion after 24 hours with excellent yields. With 1,4-dioxane, toluene, cyclopentyl methyl ether and *n-*BuOH as the solvent some starting product was recovered, however, with a good mass balance suggesting that the reaction is just slower in these solvents than in DMSO. From this we can conclude that the reaction is compatible with a variety of other solvents. In addition to the above results, Kappe et al. successfully applied our protocol in a flow process [46]. They intensified the process by working at 200 °C which allowed them to lower the catalyst loading (FeCl_3_) to 5 mol % and to omit acetic acid as the activator. As DMSO degraded and produced repulsive odors at these high temperatures the authors switched to propylene carbonate as the solvent.

**Table 4:** An extended solvent screening for the base metal-catalyzed aerobic oxidation reaction.^a^

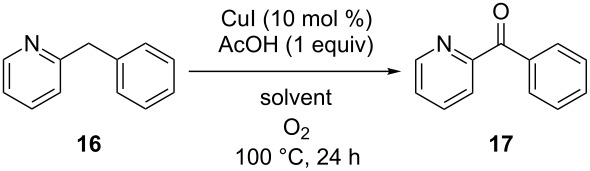			

Entry	Solvent^b^	Yield **16** (%)^c^	Yield **17** (%)^c^

1	DMSO^d^	0	87
2	anisole^e^	0	85
3	*n-*BuOAc^e^	0	89
4	1,4-dioxane^f^	2	85
5	toluene^d^	18	67
6	CPME^d^	5	80
7	*n-*BuOH^e^	5	73

^a^Reactions were performed on a 5 mmol scale in 10 mL of solvent using 1 atmosphere of O_2_ (balloon). ^b^Classification of solvents as provided in [44]. ^c^Isolated yields. ^d^Problematic. ^e^Recommended. ^f^Hazardous.

### Chemoselectivity

When multiple activated methylene motifs are present the chemoselective oxidation of one of these positions can be achieved as we previously exemplified for 2-methyl-6-(4-methylbenzyl)pyridine (**18b**, Table 5, entries 4 and 5). This interesting selectivity was further expanded on 6-(4-methylbenzyl)-2-methylpyrazine (**18a**). Pyrazine **18a** features three possible positions for methylene oxidation: a benzyl, benzhydryl and a 1,4-diazinylmethyl moiety. When **18a** was submitted to the Cu-catalyzed reaction conditions at 130 °C, only the bis-oxidation product 6-(4-methylbenzoyl)pyrazine-2-carbaldehyde (**20a**) was obtained in 61% (Table 5, entry 1). Interestingly, when switching to FeCl_2_·4H_2_O as the catalyst in the reaction, only mono-oxidation at the benzhydrylic methylene occurred, providing (6-methylpyrazin-2-yl)(*p*-tolyl)methanone (**19a**) in 57% yield (Table 5, entry 2). A similar chemoselectivity was observed for the oxidation of **18b** where Cu catalysis lead to bis-oxidation (**20b**, Table 5, entry 4) while Fe catalysis resulted in mono-oxidation (**19b**, Table 5, entry 5).

**Table 5:** Chemoselectivity obtained by selection of catalyst and solvent.^a^

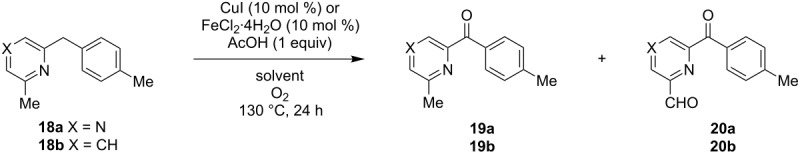					

Entry	Substrate	Catalyst	Solvent	Yield **19** (%)^b^	Yield **20** (%)^b^

1	**18a**	CuI	DMSO	0	61
2	**18a**	FeCl_2_·4H_2_O	DMSO	57	0
3	**18a**	CuI	*n-*BuOAc	78^c^	0
4	**18b**^d^	CuI	DMSO	0	62
5	**18b**^d^	FeCl_2_·4H_2_O	DMSO	85	0
6	**18b**	CuI	*n-*BuOAc	64	0

^a^Reactions were performed on a 0.5 mmol scale in 1 mL of solvent using 1 atmosphere of O_2_ (balloon). ^b^Isolated yields. ^c^48 h. ^d^Reported in our communication, see [25].

When the reaction with **18a** using Cu catalysis is performed in *n-*BuOAc as solvent instead of DMSO only compound **19a** was isolated after 48 hours of reaction (starting material was still present after 24 hours, Table 5, entry 3). The same trend was observed in the reaction of **18b**. Here under Cu catalysis at 130 °C in *n-*BuOAc also only benzhydrylic oxidation occurred and (6-methylpyridin-2-yl)(*p-*tolyl)methanone (**19b**) was isolated in 64% yield as the sole product. These results demonstrate that the oxidation power of the catalytic system can be tuned by careful selection of the solvent as well as the base metal.

## Conclusion

This work shows that the oxidation protocol disclosed in 2012 by our group can be applied to a much broader substrate scope than originally investigated. Furthermore we have shown that when the nature of the substituents does not permit full conversion after 24 hours, the standard conditions can be easily amended to increase the rate of the reaction. ICP–MS analysis was performed on a representative set of molecules from the scope disclosed to determine the Cu or Fe impurities remaining after work-up of the reaction products. This revealed that only low amounts remained, that are well below the regulatory limits. In addition, for papaveraldine a comparison between different purification procedures was performed in order to determine their influence on the amount of metal impurity remaining. While the reaction is compatible with a large number of solvents including sustainable ones, DMSO appears to give the fastest reactions. This is also reflected in the chemoselectivity studies where DMSO is the only solvent in which oxidation of a (di)azinylmethyl is possible.

## Supporting Information

File 1 Experimental procedures, compound characterization data and copies of ^1^H and ^13^C NMR spectra of all new starting materials and reaction products.
