# Supplementary material for: Base metal-catalyzed benzylic oxidation of (aryl)(heteroaryl)methanes with molecular oxygen
Source: Beilstein J Org Chem. 2016 Jan 27;12:144–53. doi: 10.3762/bjoc.12.16 (PMC4734388; doi:10.3762/bjoc.12.16)

# Supporting Information

## for

# Base metal-catalyzed benzylic oxidation of (aryl)(heteroaryl)methanes with molecular oxygen

Hans Sterckx, Johan De Houwer, Carl Mensch, Wouter Herrebout, Kourosch Abbaspour Tehrani and Bert U. W. Maes*

Address: Department of Chemistry, University of Antwerp, Groenenborgerlaan 171, B-2020 Antwerp, Belgium

Email: Bert U. W. Maes - bert.maes@uantwerpen.be

* Corresponding author

**Experimental procedures, compound characterization data and copies of $^{1}$H and $^{13}$C NMR spectra of all new starting materials and reaction products**

## Table of Contents

I. General considerations........S2

II. Synthetic experimental procedures and NMR data........S2

III. ICP-MS analysis results........S20

IV. In situ IR reaction monitoring........S21

V. DFT calculation of equilibrium constants........S21

VI. References........S26

VII. NMR Spectra........S27

# I. General considerations

Melting points were determined on an automated apparatus and are uncorrected. The $^{1}$H NMR and $^{13}$C NMR spectra were recorded on a 400 MHz spectrometer with TMS as the internal standard in the solvent indicated (for $^{13}$C spectra the solvent was chosen as a reference ($CDCl_3$ = 77.16 ppm)). Coupling constants are given in Hertz and the chemical shifts are given in ppm. For high resolution mass spectrometric analysis, samples were dissolved in $CH_3OH$ and diluted to a concentration of approximately $10^{-5}$ mol/L and 2 µL were injected using the CapLC system and electrosprayed through the nanoelectrospray source. The nanoelectrospray source operated in the positive ion mode at an electrospray potential of 1.7 kV. The eluent used was 30% A ($H_2O$, 0.1% formic acid) and 70% B ($CH_3OH/H_2O$ 95:5, 0.1% formic acid) at a flow rate of 6 µL/min. Samples were injected with an interval of 3 minutes. Before analysis and after each seventh sample a 2 µL volume of a 0.025% $H_3PO_4$ solution (MeOH/$H_2O$ 50:50) was injected and used as lock mass. The MS was calibrated prior to use with a 0.015% $H_3PO_4$ solution. The spectra were lock-mass corrected using the known mass of the nearest $H_3PO_4$ cluster. Flash column chromatography was performed on an automated chromatography system with silica flash cartridges. All chemicals, which are not described in the experimental part, were obtained from commercial sources and used as such. The oxygen used was standard industrial oxygen.

# II. Synthetic experimental procedures and NMR data

### 1. General procedure A (oxidation):

Two 10 mL vials were subsequently charged with CuI (0.0095 g, 0.05 mmol, 10 mol %) or $FeCl_2 \cdot 4H_2O$ (0.0094 g, 0.05 mmol, 10 mol %), substrate (0.5 mmol), DMSO (1 mL) and acetic acid (0.030 g, 0.5 mmol). The vials were flushed with $O_2$ for 1 min, capped with an aluminum crimp cap/septum and finally stirred at 100 °C for 24 h in an oil bath with a balloon filled with $O_2$ through the septum. After cooling to room temperature, the content of the vials was transferred into a separation funnel and the vials were rinsed with dichloromethane (20 mL). Sodium bicarbonate solution (10 mL, sat.) was added and the organic phase was separated. The aqueous phase was extracted twice with dichloromethane (10 mL). The combined organic fractions were washed with brine (20 mL), dried over $MgSO_4$ and filtered over a pad of Celite®. The solvent was removed under reduced pressure and the resulting residue was purified by column chromatography with an automated chromatography system using a silica flash cartridge (40 g) applying a heptane/ethyl acetate gradient (from 100% heptane to 100% ethyl acetate in 25 minutes, 25 mL/min).

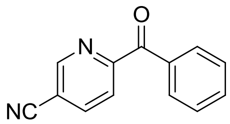


6-Benzoylpyridine-3-carbonitrile (**4a**): General procedure A was followed using 6-benzylpyridine-3-carbonitrile (**3a**, 0.097 g, 0.5 mmol). 6-Benzoylpyridine-3-carbonitrile (**4a**) was isolated as a white solid in 66% yield (83% after 48 h, CuI) and 67% yield ($FeCl_2 \cdot 4H_2O$).

**$^{1}$H-NMR ($CDCl_3$, 400 MHz)** $\delta_H$: 8.99 (dd, 1H, *J* = 2.0, 1.0 Hz), 8.20 (dd, 1H, *J* = 8.2, 2.0 Hz), 8.15 (dd, 1H, 8.1, 1.0 Hz), 8.08-8.04 (m, 2H), 7.65 (tt, 1H, *J* = 7.5, 1.4 Hz), 7.52 (tt, 2H, *J* = 8.0, 1.6 Hz).
**$^{13}$C-NMR ($CDCl_3$, 100 MHz)** $\delta_C$: 192.1, 157.4, 151.2, 140.6, 135.2, 133.8, 131.1, 128.5, 124.4, 116.1, 112.1.
HRMS (ESI) for $C_{13}H_9N_2O[M+H]^+$ calcd 209.0709, found 209.0718. Melting point: 93 °C. No spectroscopic data are available in literature.

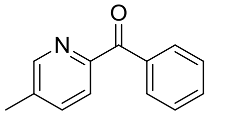

(5-Methylpyridin-2-yl)(phenyl)methanone (**4b**): General procedure A was followed using 2-benzyl-5-methylpyridine (**3b**, 0.092 g, 0.5 mmol). (5-Methylpyridin-2-yl)(phenyl)methanone (**4b**) was isolated as a colorless oil in 82% yield (CuI) and 73% yield ($FeCl_2 \cdot 4H_2O$).

**$^{1}$H-NMR ($CDCl_3$, 400 MHz)** $\delta_H$: 8.54 (dd, 1H, $J$ = 1.5, 0.7 Hz), 8.08-8.04 (m, 2H), 7.96 (bd, 1H, $J$ = 8.0 Hz), 7.68 (bd, 1H, $J$ = 8.0 Hz), 7.57 (tt, 1H, $J$ = 7.4, 1.4 Hz), 7.50-7.43 (tt, 2H, $J$ = 7.7, 1.1 Hz), 2.43 (s, 3H).
**$^{13}$C-NMR ($CDCl_3$, 100 MHz)** $\delta_C$: 193.8, 152.5, 149.1, 137.4, 136.6, 136.6, 132.7, 130.9, 128.1, 124.4, 18.7.
HRMS (ESI) for $C_{13}H_{12}NO[M+H]^+$, calcd 198.0919, found 198.0910. No spectroscopic data are available in literature.

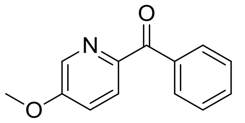

(5-Methoxypyridin-2-yl)(phenyl)methanone (**4c**): General procedure A was followed using 2-benzyl-5-methoxypyridine (**3c**, 0.100 g, 0.5 mmol). (5-Methoxypyridin-2-yl)(phenyl)methanone (**4c**) was isolated as a brown oil in 15% yield (65% using 3 equiv of AcOH at 130 °C) (CuI) and 15% yield ($FeCl_2 \cdot 4H_2O$).

**$^{1}$H-NMR ($CDCl_3$, 400 MHz)** $\delta_H$: 8.38 (bd, 1H, $J$ = 2.8 Hz), 8.11 (bd, 1H, $J$ = 8.7 Hz), 8.07-8.04 (m, 2H), 7.56 (tt, 1H, $J$ = 7.4, 1.3 Hz), 7.50-7.44 (m, 2H), 7.33 (dd, 1H, $J$ = 8.7, 2.9 Hz), 3.94 (s, 3H).
**$^{13}$C-NMR ($CDCl_3$, 100 MHz)** $\delta_C$: 192.9, 157.8, 147.6, 137.0, 136.7, 132.5, 130.9, 128.1, 126.4, 120.2, 55.9.
HRMS (ESI) for $C_{13}H_{12}NO_2[M+H]^+$, calcd 214.0868, 214.0872. No spectroscopic data are available in literature.

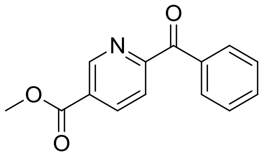

Methyl 6-benzoylpyridine-3-carboxylate (**4d**): General procedure A was followed using methyl 6-benzylpyridine-3-carboxylate (**3d**, 0.114 g, 0.5 mmol). Methyl 6-benzoylpyridine-3-carboxylate (**4d**) was isolated as a grey solid in 62% yield (CuI) and 69% yield ($FeCl_2 \cdot 4H_2O$).

**$^{1}$H-NMR ($CDCl_3$, 400 MHz)** $\delta_H$: 9.30 (d, 1H, $J$ = 1.3 Hz), 8.50 (dd, 1H, $J$ = 8.1, 2.1 Hz), 8.12-8.04 (m, 3H), 7.62 (tt, 1H, $J$ = 7.4, 1.3 Hz), 7.53-7.47 (m, 2H), 4.00 (s, 3H).
**$^{13}$C-NMR ($CDCl_3$, 100 MHz)** $\delta_C$: 193.1, 165.2, 158.0, 149.7, 138.3, 135.8, 133.4, 131.1, 128.3, 127.8, 124.2, 52.8.
HRMS (ESI) for $C_{14}H_{12}N_1O_3[M+H]^+$ calcd 242.0812, found 242.0818. Melting point: 109 °C. No spectroscopic data are available in literature.

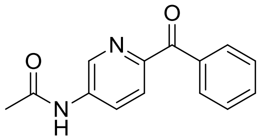

*N*-(6-Benzoylpyridin-3-yl)acetamide (**4e**): General procedure A was followed using *N*-(6-benzylpyridin-3-yl)acetamide (**3e**, 0.113 g, 0.5 mmol). *N*-(6-Benzoylpyridin-3-yl)acetamide (**4e**) was isolated as a white solid in 56% yield (91% after 48 h) (CuI) and 64% yield ($FeCl_2 \cdot 4H_2O$).

**$^{1}$H-NMR ($CDCl_3$, 400 MHz)** $\delta_H$: 8.70 (d, 1H, $J$ = 2.5 Hz), 8.39 (dd, 1H, $J$ = 8.6, 2.1 Hz), 8.11 (d, 1H, $J$ = 8.6 Hz), 8.06 (m, 2H), 7.60 (t, 2H, $J$ = 7.4 Hz)), 7.50 (t, 2H, $J$ = 7.6 Hz), 2.29 (s, 3H).
**$^{13}$C-NMR ($CDCl_3$, 100 MHz)** $\delta_C$: 192.7, 168.8, 150.1, 139.2, 137.1, 136.6, 132.7, 130.9, 128.1, 126.9, 125.7, 24.6.
HRMS (ESI) for $C_{14}H_{13}N_2O_2[M+H]^+$ calcd 241.0972, found 241.0977. Melting point: 149 °C. No spectroscopic data are available in literature.

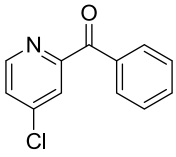

(4-Chloropyridin-2-yl)(phenyl)methanone (**4f**): General procedure A was followed using 2-benzyl-4-chloropyridine (**3f**, 0.102 g, 0.5 mmol). (4-Chloropyridin-2-yl)(phenyl)methanone (**4f**) was isolated as a yellow solid in 88% yield (CuI) and 85% yield ($FeCl_2{\cdot}4H_2O$).

**$^{1}$H-NMR ($CDCl_3$, 400 MHz)** $\delta_H$: 8.60 (dd, 1H, *J* = 5.2, 0.4 Hz), 8.09-8.05 (m, 2H), 8.03 (dd, 1H, *J* = 2.1, 0.6 Hz) 7.62–7.56 (m, 1H), 7.51-7.45 (m, 3H).
**$^{13}$C-NMR ($CDCl_3$, 100 MHz)** $\delta_C$: 192.4, 156.3, 149.5, 145.5, 135.7, 133.3, 131.0, 128.2, 126.3, 125.0.
HRMS (ESI) for $C_{12}H_9NOCl[M+H]^+$ calcd 218.0367, found 218.0368. Melting point: 63 °C. The reported spectroscopic data are consistent with the available literature data [4].

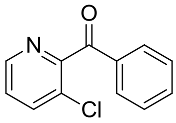

(3-Chloropyridin-2-yl)(phenyl)methanone (**4g**): General procedure A was followed using 2-benzyl-3-chloropyridine (**3g**, 0.102 g, 0.5 mmol). (3-Chloropyridin-2-yl)(phenyl)methanone (**4g**) was isolated as a yellow liquid in 22% yield (87% using 3 equiv of AcOH at 130 °C) (CuI) and 23% yield ($FeCl_2{\cdot}4H_2O$).

**$^{1}$H-NMR ($CDCl_3$, 400 MHz)** $\delta_H$: 8.58 (dd, 1H, *J* = 4.7, 1.4 Hz), 7.88-7.81 (m, 3H), 7.64-7.58 (m, 1H), 7.51-7.44 (m, 2H), 7.40 (dd, 1H, *J* = 8.2, 4.6 Hz).
**$^{13}$C-NMR ($CDCl_3$, 100 MHz)** $\delta_C$: 192.6, 154.6, 147.1, 138.1, 135.3, 134.1, 130.4, 129.7, 128.7, 125.5.
HRMS (ESI) for $C_{12}H_9NOCl[M+H]^+$ calcd 218.0367, found 218.0369. The reported spectroscopic data are consistent with the available literature data [5].

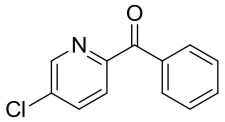

(5-Chloropyridin-2-yl)(phenyl)methanone (**4h**): General procedure A was followed using 2-benzyl-5-chloropyridine (**3h**, 0.102 g, 0.5 mmol). (5-Chloropyridin-2-yl)(phenyl)methanone (**4h**) was isolated as a white solid in 15% yield (92% using 3 equiv of AcOH at 130 °C) (CuI) and 20% yield ($FeCl_2{\cdot}4H_2O$).

**$^{1}$H-NMR ($CDCl_3$, 400 MHz)** $\delta_H$: 8.67 (d, 1H, *J* = 1.9 Hz), 8.09-8.00 (m, 3H), 7.87 (dd, 1H, *J* = 8.4, 2.4 Hz), 7.60 (tt, 1H, *J* = 7.4, 1.3 Hz), 7.52-7.46 (m , 2H).
**$^{13}$C-NMR ($CDCl_3$, 100 MHz)** $\delta_C$: 192.56, 153.0, 147.5, 136.9, 136.0, 135.2, 133.1, 131.0, 128.2, 125.6.
HRMS (ESI) for $C_{12}H_9NOCl[M+H]^+$ calcd 218.0367, found 218.0368. Melting point: 62 °C. The reported spectroscopic data are consistent with the available literature data [6].

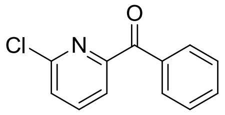

(6-Chloropyridin-2-yl)(phenyl)methanone (**4i**): General procedure A (*T* = 130 °C and 3 equiv of AcOH) was followed using 2-benzyl-6-chloropyridine (**3i**, 0.102 g, 0.5 mmol). (6-Chloropyridin-2-yl)(phenyl)methanone (**4i**) was isolated as a brown solid in 29% yield (CuI).

**$^{1}$H-NMR ($CDCl_3$, 400 MHz)** $\delta_H$: 8.13-8.08 (m, 2H), 7.95 (dd, 1H, *J* = 7.60, 0.88 Hz), 7.86 (t, 1H, *J* = 7.80 Hz), 7.65-7.57 (m, 1H), 7.54-7.46 (m, 3H).
**$^{13}$C-NMR ($CDCl_3$, 100 MHz)** $\delta_C$: 191.8, 155.3, 150.3, 139.6, 135.5, 133.3, 131.1, 128.3, 127.1, 123.1.
HRMS (ESI) for $C_{12}H_9N_1OCl[M+H]^+$ calcd 218.0367, found 218.0380. Melting point: 56 °C. The reported spectroscopic data are consistent with the available literature data [6].

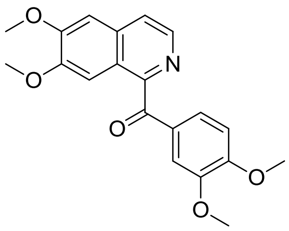

(6,7-Dimethoxyisoquinolin-1-yl)(3,4-dimethoxyphenyl)methanone (**15**): General procedure A was followed using 1-(3,4-dimethoxybenzyl)-6,7-dimethoxyisoquinoline (**14**, papaverine, 0.170 g, 0.5 mmol). (6,7-Dimethoxyisoquinolin-1-yl)(3,4-dimethoxyphenyl)methanone (**15**) (papaveral-dine) was isolated as a brown solid in 60% yield ($FeCl_2{\cdot}4H_2O$).

**$^1$H-NMR ($CDCl_3$, 400 MHz)** $\delta_H$: 8.46 (d, 1H, *J* = 5.5 Hz), 7.71 (d, 1H, *J* = 1.9 Hz), 7.64 (d, 1H, *J* = 5.5 Hz), 7.55 (s, H), 7.43 (dd, 1H, *J* = 8.4, 2.0 Hz), 7.14 (s, 1H), 6.87 (d, 1H, *J* = 8.5 Hz), 4.05 (s, 3H), 3.96 (bs, 6H), 3.94 (s, 3H).
**$^{13}$C-NMR ($CDCl_3$, 100 MHz)** $\delta_C$: 194.0, 153.9, 153.8, 153.2, 151.1, 149.1, 140.1, 134.0, 130.0, 126.9, 122.9, 121.2, 112.1, 110.0, 104.9, 104.2, 56.1, 56.1, 56.1, 56.0.
HRMS (ESI) for $C_{20}H_{20}NO_5[M+H]^+$ calcd 354.1336, found 354.1353. Melting point: 203 °C. The reported spectroscopic data are consistent with the available literature data [7].

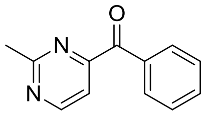

(2-Methylpyrimidin-4-yl)(phenyl)methanone (**6f**): General procedure A (*T* = 130 °C) was followed using 4-benzyl-2-methylpyrimidine (**5f**, 0.092 g, 0.5 mmol). (2-Methylpyrimidin-4-yl)(phenyl)methanone (**6f**) was isolated as a yellow liquid in 40% yield ($FeCl_2{\cdot}4H_2O$).

**$^1$H-NMR ($CDCl_3$, 400 MHz)** $\delta_H$: 8.90 (d, 1H, *J* = 5.0 Hz), 8.10 (d, 2H, *J* = 7.5 Hz), 7.66-7.60 (m, 2H), 7.50 (t, 2H, *J* = 7.6 Hz), 2.83 (s, 3H).
**$^{13}$C-NMR ($CDCl_3$, 100 MHz)** $\delta_C$: 192.5, 167.9, 161.8, 158.7, 134.9, 133.8, 131.0, 128.4, 117.0, 26.1.
HRMS (ESI) for $C_{12}H_{11}N_2O[M+H]^+$ calcd 199.0866, found 199.0869. No spectroscopic data are available in literature.

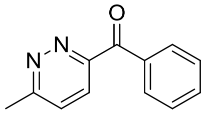

(6-Methylpyridazin-3-yl)(phenyl)methanone (**6g**): General procedure A was followed using 3-benzyl-6-methylpyridazine (**5g**, 0.092 g, 0.5 mmol). (6-Methylpyridazin-3-yl)(phenyl)methanone (**6g**) was isolated as a brown solid in 81% yield ($FeCl_2{\cdot}4H_2O$).

**$^1$H-NMR ($CDCl_3$, 400 MHz)** $\delta_H$: 8.26-8.19 (m, 2H), 8.07 (d, 1H, *J* = 8.57 Hz), 7.62 (tt, 1H, *J* = 7.44, 1.26 Hz), 7.54 (d, 1H, *J* = 8.60 Hz), 7.54-7.47 (m, 2H), 2.83 (s, 3H).
**$^{13}$C-NMR ($CDCl_3$, 100 MHz)** $\delta_C$: 191.9, 161.7, 156.0, 135.6, 133.4, 131.4, 128.3, 127.6, 127.4, 22.5.
HRMS (ESI) for $C_{12}H_{11}N_2O[M+H]^+$ calcd 199.0806, found 199.0871. Melting point: 69 °C. No spectroscopic data are available in literature.

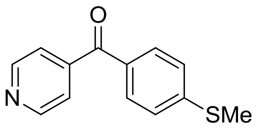


[4-(Methylthio)phenyl](pyridin-4-yl)methanone (**2c**): General procedure A was followed using 4-[4-(methylthio)benzyl]pyridine (**1c**, 0.108 g, 0.5 mmol). [4-(Methylthio)phenyl](pyridin-4-yl)methanone (**2c**) was isolated as a white solid in 56% yield ($FeCl_2{\cdot}4H_2O$).

**$^1$H-NMR ($CDCl_3$, 400 MHz)** $\delta_H$: 8.80 (dd, 2H, *J* = 4.4, 1.6 Hz), 7.75 (bd, 2H, *J* = 8.6 Hz), 7.55 (dd, 2H, *J* = 4.4, 1.6 Hz), 7.31 (bd, 2H, *J* = 8.6 Hz), 2.55 (s, 3H).
**$^{13}$C-NMR ($CDCl_3$, 100 MHz)** $\delta_C$: 194.2, 150.4, 147.2, 144.9, 132.0, 130.7, 125.0, 122.8, 14.8.
HRMS (ESI) for $C_{13}H_{12}NOS$ $[M+H]^+$, calcd 230.0634, found 230.0638. Melting point: 135 °C. The reported spectroscopic data are consistent with the available literature data [8].

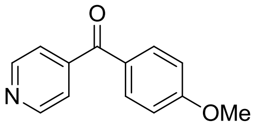

(4-Methoxyphenyl)(pyridin-4-yl)methanone (**2d**): General procedure A was followed using 4-(4-methoxybenzyl)pyridine (**1d**, 0.100 g, 0.5 mmol). (4-Methoxyphenyl)(pyridin-4-yl)methanone (**2d**) was isolated as a white solid in 67% yield ($FeCl_2{\cdot}4H_2O$).

**$^1$H-NMR ($CDCl_3$, 400 MHz)** $\delta_H$: 8.78 (dd, 2H, *J* = 4.4, 1.6 Hz), 7.82 (bd, 2H, *J* = 8.9 Hz), 7.52 (dd, 2H, *J* = 4.4, 1.6 Hz), 6.98 (bd, 2H, *J* = 8.9 Hz), 3.89 (s, 3H).
**$^{13}$C-NMR ($CDCl_3$, 100 MHz)** $\delta_C$: 193.5, 164.0, 150.2, 145.2, 132.6, 128.6, 122.7, 113.9, 55.6.
HRMS (ESI) for $C_{13}H_{12}NO_2$ $[M+H]^+$, calcd 214.0863, found 214.0869. Melting point: 118-121 °C. The reported spectroscopic data are consistent with the available literature data [8].

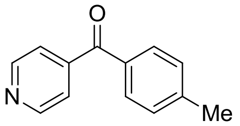

(4-Methylphenyl)(pyridin-4-yl)methanone (**2e**): General procedure A was followed using 4-(4-methylbenzyl)pyridine (**1e**, 0.092 g, 0.5 mmol). (4-Methylphenyl)(pyridin-4-yl)methanone (**2e**) was isolated as a white solid in 79% yield ($FeCl_2{\cdot}4H_2O$).

**$^1$H-NMR ($CDCl_3$, 400 MHz)** $\delta_H$: 8.80 (dd, 2H, *J* = 4.4, 1.6 Hz), 7.73 (bd, 2H, *J* = 8.2 Hz), 7.56 (dd, 2H, *J* = 4.4, 1.6 Hz), 7.31 (bd, 2H, *J* = 8.0 Hz), 2.45 (s, 3H).
**$^{13}$C-NMR ($CDCl_3$, 100 MHz)** $\delta_C$: 194.8, 150.3, 144.8, 144.7, 133.3, 130.4, 129.4, 122.9, 21.8.
HRMS (ESI) for $C_{13}H_{12}NO$ $[M+H]^+$, calcd 198.0913, found 198.0913. Melting point: 93-95 °C. The reported spectroscopic data are consistent with the available literature data [8].

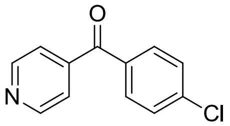

(4-Chlorophenyl)(pyridin-4-yl)methanone (**2h**): General procedure A was followed using 4-(4-chlorobenzyl)pyridine (**1h**, 0.102 g, 0.5 mmol). (4-Chlorophenyl)(pyridin-4-yl)methanone (**2h**) was isolated as a white solid in 66% yield ($FeCl_2{\cdot}4H_2O$).

**$^1$H-NMR ($CDCl_3$, 400 MHz)** $\delta_H$: 8.82 (dd, 2H, *J* = 4.4, 1.6 Hz), 7.77 (bd, 2H, *J* = 8.6 Hz), 7.55 (dd, 2H, *J* = 4.4, 1.6 Hz), 7.50 (bd, 2H, *J* = 8.6 Hz).
**$^{13}$C-NMR ($CDCl_3$, 100 MHz)** $\delta_C$: 194.0, 150.6, 144.1, 140.3, 134.3, 131.6, 129.2, 122.8.
HRMS (ESI) for $C_{12}H_9ClNO$ $[M+H]^+$, calcd 218.0367, found 218.0372. Melting point: 110 °C. The reported spectroscopic data are consistent with the available literature data [9].

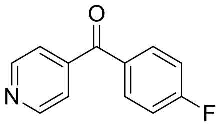

(4-Fluorophenyl)(pyridin-4-yl)methanone (**2i**): General procedure A was followed using 4-(4-fluorobenzyl)pyridine (**1i**) (0.094 g, 0.5 mmol). (4-Fluorophenyl)(pyridin-4-yl)methanone (**2i**) was isolated as a white solid in 76% yield ($FeCl_2{\cdot}4H_2O$).

**$^1$H-NMR ($CDCl_3$, 400 MHz)** $\delta_H$: 8.82 (dd, 2H, *J* = 4.4, 1.6 Hz), 7.87 (dd, 2H, *J* = 8.9, 5.3 Hz), 7.55 (dd, 2H, *J* = 4.4, 1.6 Hz), 7.20 (bt, 2H, *J* = 8.5 Hz).
**$^{13}$C-NMR ($CDCl_3$, 100 MHz)** $\delta_C$: 193.7, 165.6 (d, $J_{CF}$ = 256 Hz), 150.5, 144.4, 132.9 (d, $J_{CF}$ = 9.4 Hz), 132.4 (d, $J_{CF}$ = 2.9 Hz), 122.8, 116.0 (d, $J_{CF}$ = 22 Hz).
HRMS (ESI) for $C_{12}H_9FNO$ $[M+H]^+$, calcd 202.0663, found 202.0659. Melting point: 87 °C. No spectroscopic data are available in literature.

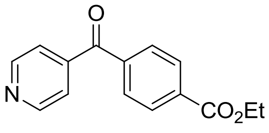

Ethyl 4-(pyridin-4-ylcarbonyl)benzoate (**2j**): General procedure A was followed using ethyl 4-(pyridin-4-ylmethyl)benzoate (**1j**, 0.121 g, 0.5 mmol). Ethyl 4-(pyridin-4-ylcarbonyl)benzoate (**2j**) was isolated as a faint yellow solid in 61% yield ($FeCl_2 \cdot 4H_2O$).

**$^1$H-NMR (CDCl$_3$, 400 MHz)** $\delta_H$: 8.84 (dd, 2H, $J$ = 4.4, 1.6 Hz), 8.18 (bd, 2H, $J$ = 8.5 Hz), 7.86 (bd, 2H, $J$ = 8.6 Hz), 7.58 (dd, 2H, $J$ = 4.4, 1.6 Hz), 4.44 (q, 2H, $J$ = 7.1 Hz), 1.43 (t, 3H, $J$ = 7.1 Hz).
**$^{13}$C-NMR (CDCl$_3$, 100 MHz)** $\delta_C$: 194.7, 165.6, 150.7, 143.8, 139.4, 134.7, 129.9, 122.9, 61.7, 14.4.
HRMS (ESI) for $C_{15}H_{14}NO_3$ $[M+H]^+$, calcd 256.0968, found 256.0972. Melting point: 64-66 °C. No spectroscopic data are available in literature.

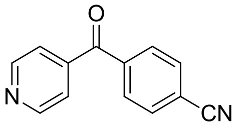

4-(Pyridin-4-ylcarbonyl)benzonitrile (**2k**): General procedure A was followed using methyl 4-(pyridin-4-ylmethyl)benzonitrile (**1k**, 0.097 g, 0.5 mmol). 4-(Pyridin-4-ylcarbonyl)benzonitrile (**2k**) was isolated as a faint yellow solid in 79% yield ($FeCl_2 \cdot 4H_2O$).

**$^1$H-NMR (CDCl$_3$, 400 MHz)** $\delta_H$: 8.86 (dd, 2H, $J$ = 4.4, 1.6 Hz), 7.91 (bd, 2H, $J$ = 8.6 Hz), 7.83 (bd, 2H, $J$ = 8.6 Hz), 7.57 (dd, 2H, $J$ = 4.4, 1.7 Hz).
**$^{13}$C-NMR (CDCl$_3$, 100 MHz)** $\delta_C$: 193.7, 150.8, 143.0, 139.4, 132.6, 130.4, 122.7, 117.7, 116.9.
HRMS (ESI) for $C_{13}H_9N_2O$ $[M+H]^+$, calcd 209.0709, found 209.0711. Melting point: 130-132 °C. No spectroscopic data are available in literature.

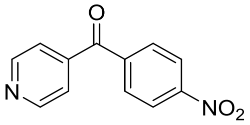

(4-Nitrophenyl)(pyridin-4-yl)methanone (**2l**): General procedure A was followed using 4-(4-nitrobenzyl)pyridine (**1l**, 0.107 g, 0.5 mmol). (4-Nitrophenyl)(pyridin-4-yl)methanone (**2l**) was isolated as a white solid in 60% yield ($FeCl_2 \cdot 4H_2O$).

**$^1$H-NMR (CDCl$_3$, 400 MHz)** $\delta_H$: 8.88 (dd, 2H, $J$ = 4.5, 1.5 Hz), 8.38 (bd, 2H, $J$ = 8.7 Hz), 7.98 (bd, 2H, $J$ = 8.7 Hz), 7.59 (dd, 2H, $J$ = 4.5, 1.5 Hz).
**$^{13}$C-NMR (CDCl$_3$, 100 MHz)** $\delta_C$: 193.6, 150.9, 150.5, 142.9, 140.9, 131.0, 124.0, 122.7.
HRMS (ESI) for $C_{12}H_9N_2O_3$ $[M+H]^+$, calcd 229.0608, found 229.0607. mp: 120-123 °C. The reported spectroscopic data are consistent with the available literature data [8].

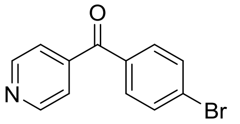

(4-Bromophenyl)(pyridin-4-yl)methanone (**2g**): General procedure A was followed using 4-(4-bromobenzyl)pyridine (**1g**, 0.124 g, 0.5 mmol). (4-Bromophenyl)(pyridin-4-yl)methanone (**2g**) was isolated as a white solid in 85% yield ($FeCl_2 \cdot 4H_2O$).

**$^1$H-NMR (CDCl$_3$, 400 MHz)** $\delta_H$: 8.82 (dd, 2H, $J$ = 4.4, 1.5 Hz), 7.71-7.65 (m, 4H), 7.55 (dd, 2H, $J$ = 4.4, 1.6 Hz).
**$^{13}$C-NMR (CDCl$_3$, 100 MHz)** $\delta_C$: 194.0, 150.1, 144.4, 134.6, 132.2, 131.6, 129.1, 123.0.
HRMS (ESI) for $C_{12}H_9BrNO$ $[M+H]^+$, calcd 261.9862, found 261.9861. mp: 122-125 °C. No spectroscopic data are available in literature.

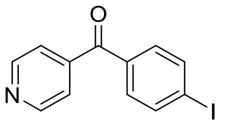

Iodophenyl)(pyridin-4-yl)methanone (**2f**): General procedure A was followed using 4-(4-iodobenzyl)pyridine (**1f**, 0.148 g, 0.5 mmol). (4-Iodophenyl)(pyridin-4-yl)methanone (**2f**) was isolated as a white solid in 77% yield ($FeCl_2 \cdot 4H_2O$).

**$^1$H-NMR (CDCl$_3$, 400 MHz)** $\delta_H$: 8.82 (d, 2H, $J$ = 5.5 Hz), 7.89 (bd, 2H, $J$ = 8.4 Hz), 7.57-7.51 (m, 4H).
**$^{13}$C-NMR (CDCl$_3$, 100 MHz)** $\delta_C$: 193.3, 148.4, 146.6, 131.4, 130.3, 129.0, 124.2, 102.6.

HRMS (ESI) for $C_{12}H_9INO$ $[M+H]^+$, calcd 309.9723, found 309.9723. mp: 183-185 °C. No spectroscopic data are available in literature.

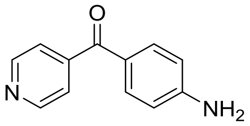

(4-Aminophenyl)(pyridin-4-yl)methanone (**2b**): General procedure A was followed using 4-(pyridin-4-ylmethyl)aniline (**1b**, 0.092 g, 0.5 mmol). (4-Aminophenyl)(pyridin-4-yl)methanone (**2b**) was isolated as a yellow solid in 55% yield ($FeCl_2 \cdot 4H_2O$).

**$^1$H-NMR ($CDCl_3$, 400 MHz)** $\delta_H$: 8.76 (dd, 2H, *J* = 4.4, 1.4 Hz), 7.70 (bd, 2H, *J* = 8.6 Hz), 7.50 (dd, 2H, *J* = 4.4, 1.5 Hz), 6.68 (bd, 2H, *J* = 8.7 Hz), 4.27 (bs, 2H).
**$^{13}$C-NMR ($CDCl_3$, 100 MHz)** $\delta_C$: 193.3, 151.9, 150.1, 146.1, 133.1, 125.9, 122.8, 113.8.
HRMS (ESI) for $C_{12}H_{11}N_2O$ $[M+H]^+$, calcd 199.0866, found 199.0869. mp: 118-120 °C. No spectroscopic data are available in literature.

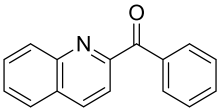

Phenyl(quinolin-2-yl)methanone (**6a**): General procedure A was followed using 2-benzylquinoline (**5a**, 0.109 g, 0.5 mmol). Phenyl(quinolin-2-yl)methanone (**6a**) was isolated as an off-white solid in 70% yield ($FeCl_2 \cdot 4H_2O$).

**$^1$H-NMR ($CDCl_3$, 400 MHz)** $\delta_H$: 8.34 (d, 1H, *J* = 8.5 Hz), 8.27-8.22 (m, 2H), 8.20 (bd, 1H, *J* = 8.5 Hz), 8.11 (d, 1H, *J* = 8.5 Hz), 7.91 (bd, 1H, *J* = 8.2 Hz), 7.78 (ddd, 1H, *J* = 8.4, 6.9, 1.4 Hz), 7.66 (ddd, 1H, *J* = 8.1, 7.0, 1.1 Hz), 7.62 (tt, 1H, *J* = 6.8, 1.3 Hz), 7.51 (bt, 2H, *J* = 7.6 Hz).
**$^{13}$C-NMR ($CDCl_3$, 100 MHz)** $\delta_C$: 193.9, 154.8, 146.8, 137.2, 136.2, 133.2, 131.5, 130.6, 130.2, 129.0, 128.5, 128.2, 127.7, 120.9.
HRMS (ESI) for $C_{16}H_{12}NO$ $[M+H]^+$, calcd 234.0913, found 234.0914. Melting point: 104-108 °C. The reported spectroscopic data are consistent with the available literature data [10].

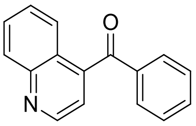

Phenyl(quinolin-4-yl)methanone (**6b**): General procedure A was followed using 4-benzylquinoline (**5b**, 0.109 g, 0.5 mmol). Phenyl(quinolin-4-yl)methanone (**6b**) was isolated as a yellow oil in 61% yield ($FeCl_2 \cdot 4H_2O$).

**$^1$H-NMR ($CDCl_3$, 400 MHz)** $\delta_H$: 9.03 (d, 1H, *J* = 4.3 Hz), 8.22 (d, 1H, *J* = 8.5 Hz), 7.89-7.83 (m, 3H), 7.77 (ddd, 1H, *J* = 8.3, 6.9, 1.3 Hz), 7.65 (tt, 1H, *J* = 7.4, 1.3 Hz), 7.54 (ddd, 1H, *J* = 8.3, 6.9, 1.2 Hz), 7.49 (bt, 2H, *J* = 8.1 Hz), 7.41 (d, 1H, *J* = 4.3 Hz).
**$^{13}$C-NMR ($CDCl_3$, 100 MHz)** $\delta_C$: 196.2, 149.6, 148.7, 144.5, 136.7, 134.3, 130.4, 130.1, 128.9, 127.8, 125.5, 125.1, 119.7.
HRMS (ESI) for $C_{16}H_{12}NO$ $[M+H]^+$, calcd 234.0913, found 234.0909. The reported spectroscopic data are consistent with the available literature data [11].

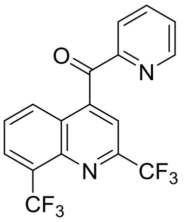

[2,8-Bis(trifluoromethyl)quinolin-4-yl](pyridin-2-yl)methanone (**10**): General procedure A was followed using 4-(pyridin-2-ylmethyl)-2,8-bis(trifluoromethyl)quinoline (**12**, 0.185 g, 0.5 mmol). Column chromatography was performed applying dichloromethane (100%) as eluent. [2,8-Bis(trifluoromethyl)quinolin-4-yl](pyridin-2-yl)methanone (**10**) was isolated as a faint brown solid in 63% yield ($FeCl_2 \cdot 4H_2O$).

**$^1$H-NMR ($CDCl_3$, 400 MHz)** $\delta_H$: 8.62 (ddd, 1H, *J* = 4.6, 1.4, 0.8 Hz), 8.36 (bd, 1H, *J* = 7.8 Hz), 8.20 (bd, 1H, *J* = 7.2 Hz), 8.13 (bd, 1H, *J* = 8.5 Hz), 8.02 (td, 1H, *J* = 7.7, 1.7 Hz), 7.91 (s, 1H), 7.69 (t, 1H, *J* = 7.8 Hz), 7.58 (ddd, 1H, *J* = 7.6, 4.7, 1.2 Hz).

**$^{13}$C-NMR (CDCl$_3$, 100 MHz)** $\delta_C$: 194.2, 153.0, 149.6, 147.7 (q, $J_{CF}$ = 35 Hz), 146.4, 144.2, 137.6, 129.8, 129.4 (q, $J_{CF}$ = 30 Hz), 129.4 (q, $J_{CF}$ = 5.5 Hz), 128.2, 128.0, 126.5, 124.2, 123.6 (q, $J_{CF}$ = 273 Hz), 121.2 (q, $J_{CF}$ = 275 Hz), 117.6 (q, $J_{CF}$ = 2.0 Hz).
HRMS (ESI) for $C_{17}H_9F_6N_2O$ [M+H]$^+$, calcd 371.0614, found 371.0618. mp: 128-130°C. The reported spectroscopic data are consistent with the available literature data [12].

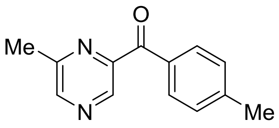


(4-Methylphenyl)(6-methylpyrazin-2-yl)methanone (**19a**): General procedure A ($T$ = 130 °C) was followed using 6-(4-methylbenzyl)-2-methylpyrazine (**18a**, 0.099 g, 0.5 mmol). (4-Methylphenyl)(6-methylpyrazin-2-yl)methanone (**19a**) was isolated as an off-white solid in 57% yield ($FeCl_2{\cdot}4H_2O$).

**$^1$H-NMR (CDCl$_3$, 400 MHz)** $\delta_H$: 8.97 (bs, 1H), 8.63 (bs, 1H), 7.99 (d, 2H, $J$ = 8.0 Hz), 7.31 (d, 2H, $J$ = 8.0 Hz), 2.66 (s, 3H), 2.44 (s, 3H).
**$^{13}$C-NMR (CDCl$_3$, 100 MHz)** $\delta_C$: 192.1, 152.6, 149.4, 146.5, 144.6, 142.7, 133.1, 131.2, 129.1, 21.8, 21.7.
HRMS (ESI) for $C_{13}H_{13}N_2O$ [M+H]$^+$, calcd 213.1022, found 213.1026. mp: 59-63 °C. No spectroscopic data are available in literature.

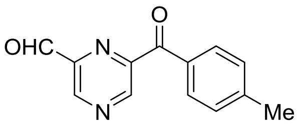


6-(4-Methylbenzoyl)pyrazine-2-carbaldehyde (**20a**): General procedure A ($T$ = 130 °C) was followed using 6-(4-methylbenzyl)-2-methylpyrazine (**18a**, 0.099 g, 0.5 mmol). 6-(4-Methylbenzoyl)pyrazine-2-carbaldehyde (**20a**) was isolated as a brown oil in 61% (CuI) yield.

**$^1$H-NMR (CDCl$_3$, 400 MHz)** $\delta_H$: 10.16 (s, 1H), 9.38 (s, 1H), 9.30 (s, 1H), 8.06-8.02 (m, 2H), 7.34-7.30 (m, 2H), 2.46 (s, 3H).
**$^{13}$C-NMR (CDCl$_3$, 100 MHz)** $\delta_C$: 191.5, 190.3, 149.7, 149.1, 144.9, 144.4, 144.4, 132.2, 130.9, 129.0, 21.6.
HRMS (ESI) for $C_{13}H_{10}N_2O_2$ [M+H]$^+$, calcd 226.0742 , found 226.0740 . No spectroscopic data are available in literature.

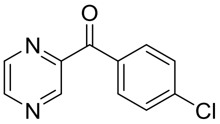


(4-Chlorophenyl)(pyrazin-2-yl)methanone (**6d**): General procedure A ($T$ = 130 °C) was followed using (4-chlorobenzyl)pyrazine (**5d**, 0.102 g, 0.5 mmol). (4-Chlorophenyl)(pyrazin-2-yl)methanone (**6d**) was isolated as an off-white solid in 92% yield ($FeCl_2{\cdot}4H_2O$).

**$^1$H-NMR (CDCl$_3$, 400 MHz)** $\delta_H$: 9.28 (s, 1H), 8.80 (s, 1H), 8.68 (s, 1H), 8.09 (bd, 2H, $J$ = 8.8 Hz), 7.49 (bd, 2H, $J$ = 8.8 Hz).
**$^{13}$C-NMR (CDCl$_3$, 100 MHz)** $\delta_C$: 190.9, 149.5, 147.1, 146.2, 142.9, 140.2, 133.9, 132.4, 128.8.
HRMS (ESI) for $C_{11}H_8ClN_2O$ [M+H]$^+$, calcd 219.0320, found 219.0325. mp: 75-77 °C. The reported spectroscopic data are consistent with the available literature data [13].

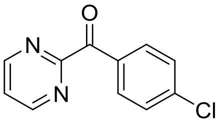


(4-Chlorophenyl)(pyrimidin-2-yl)methanone (**6c**): General procedure A was followed using 2-(4-chlorobenzyl)pyrimidine (**5c**, 0.102 g, 0.5 mmol). (4-Chlorophenyl)(pyrimidin-2-yl)methanone (**6c**) was isolated as a faint orange solid in 90% yield ($FeCl_2{\cdot}4H_2O$).

**$^1$H-NMR (CDCl$_3$, 400 MHz)** $\delta_H$: 8.95 (d, 2H, $J$ = 4.9 Hz), 8.02 (d, 2H, $J$ = 8.7 Hz), 7.51 (t, 1H, $J$ = 4.9 Hz), 7.46 (d, 2H, $J$ = 8.7 Hz).
**$^{13}$C-NMR (CDCl$_3$, 100 MHz)** $\delta_C$: 189.8, 162.1, 157.4, 140.1, 133.5, 132.3, 128.7, 122.4.

HRMS (ESI) for $C_{11}H_8ClN_2O$ $[M+H]^+$, calcd 219.0320, found 219.0323. mp: 90-93 °C. The reported spectroscopic data are consistent with the available literature data [13].

**2. General procedure B (cross coupling) [1]:**

To a flame-dried 100 mL flask under argon containing bis(triphenylphosphine)nickel(II)chloride (1.308 g, 2.0 mmol), 6-chloropyridine-3-carbonitrile (5 mmol) and dry THF (50 mL) was added benzylzinc bromide (17 mL, 0.5 M in THF, 8.5 mmol) via syringe. The mixture was stirred at rt for 48 h. After the reaction was complete, the mixture was quenched by the addition of 10% aqueous ammonium chloride (75 mL) and allowed to stir for 30 min. The mixture was extracted with ethyl acetate (150 mL), washed with brine (3 x 75 mL) and dried over $MgSO_4$. After filtration the solvent was removed by rotary evaporation to yield a liquid which was purified by flash chromatography with an automated chromatography system using a silica flash cartridge applying a heptane/ethyl acetate gradient (from 100% heptane to 100% ethyl acetate in 25 min, 25 mL/min).

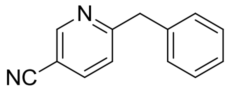


6-Benzylpyridine-3-carbonitrile (**3a**): General procedure B was followed using 6-chloropyridine-3-carbonitrile (0.693 g, 5 mmol). 6-Benzylpyridine-3-carbonitrile (**3a**) was isolated as a faint yellow oil in 57% yield.

**$^1$H-NMR ($CDCl_3$, 400 MHz)** $\delta_H$: 8.82 (s, 1H), 7.83 (dd, 1H, *J* = 8.1, 2.2 Hz), 7.35-7.29 (m, 2H), 7.28-7.19 (m, 4H), 4.22 (s, 2H).
**$^{13}$C-NMR ($CDCl_3$, 100 MHz)** $\delta_C$: 165.6, 152.2, 139.7, 137.8, 129.2, 129.0, 127.1, 123.2, 117.0, 107.6, 45.0.
HRMS (ESI) for $C_{13}H_{11}N_2$$[M+H]^+$, calcd 195.0922, found 195.0926. No spectroscopic data are available in literature.

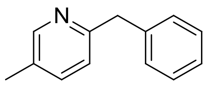


2-Benzyl-5-methylpyridine (**3b**): General procedure B was followed using 2-chloro-5-methylpyridine (0.546 mL, 5 mmol). 2-Benzyl-5-methylpyridine (**3b**) was isolated as a colorless oil in 48% yield.

**$^1$H-NMR ($CDCl_3$, 400 MHz)** $\delta_H$: 8.37 (d, 1H, *J* = 1.6 Hz), 7.40-7.36 (m , 1H), 7.32-7.23 (m, 4H), 7.21 (tt, 1H, *J* = 7.0, 1.6 Hz), 7.00 (d, 1H, *J* = 7.9 Hz), 4.12 (s, 2H), 2.28 (s, 3H).
**$^{13}$C-NMR ($CDCl_3$, 100 MHz)** $\delta_C$: 158.1, 149.7, 139.9, 137.2, 130.6, 129.1, 128.6, 126.4, 122.7, 44.3, 18.1.
HRMS (ESI) for $C_{13}H_{14}N$ $[M+H]^+$ calcd 184.1121, found 184.1120. The reported spectroscopic data are consistent with the available literature data [14].

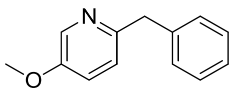


2-Benzyl-5-methoxypyridine (**3c**): General procedure B was followed using 2-bromo-5-methoxypyridine (0.940 g, 5 mmol). 2-Benzyl-5-methoxypyridine (**3c**) was isolated as a colorless oil in 59% yield.

**$^1$H-NMR ($CDCl_3$, 400 MHz)** $\delta_H$: 8.24 (d, 1H, *J* = 2.9 Hz), 7.32-7.26 (m, 2H), 7.25-7.17 (m, 3H), 7.10 (dd, 1H, *J* = 8.6, 3.0 Hz), 7.03 (d, 1H, *J* = 8.5), 4.10 (s, 2H), 3.82 (s, 3H).
**$^{13}$C-NMR ($CDCl_3$, 100 MHz)** $\delta_C$: 154.1, 153.2, 140.1, 136.6, 129.0, 128.6, 126.3, 123.3, 121.5, 55.7, 43.8.
HRMS (ESI) for $C_{13}H_{14}NO$$[M+H]^+$, calcd 200.1075, found 200.1072. No spectroscopic data are available in literature.

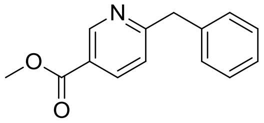

Methyl 6-benzylpyridine-3-carboxylate (**3d**): General procedure B was followed using methyl 6-chloropyridine-3-carboxylate (0.858 g, 5 mmol). Methyl 6-benzylpyridine-3-carboxylate (**3d**) was isolated as a colorless oil in 75% yield.

**$^1$H-NMR ($CDCl_3$, 400 MHz)** $\delta_H$: 9.15 (d, 1H, *J* = 1.7 Hz), 8.17 (dd, 1H, *J* = 8.1, 2.2 Hz), 7.34-7.28 (m, 2H), 7.27-7.20 (m, 3H), 7.18 (d, 1H, *J* = 8.1 Hz), 4.21 (s, 2H), 3.93 (s, 3H).
**$^{13}$C-NMR ($CDCl_3$, 100 MHz)** $\delta_C$: 165.9, 165.6, 150.7, 138.6, 137.7, 129.2, 128.8, 126.8, 123.9, 122.8, 52.4, 44.9.
HRMS (ESI) for $C_{14}H_{14}NO_2[M+H]^+$, calcd 228.1025, found 228.1030. No spectroscopic data are available in literature.

**3. General procedure C (cross coupling):**

In a flame-dried 100 mL flask under argon containing tetrakis(triphenylphosphine)palladium(0) (0.289 g, 0.250 mmol), 2-bromo-4-chloropyridine (5 mmol) and dry THF (30 mL) were stirred until a clear solution was obtained. Next, benzylzinc bromide (13 mL, 0.5 M in THF, 6.50 mmol) was added via a syringe. The mixture was stirred at 70 °C for 18 h. After the reaction was complete the mixture was quenched by the addition of 10% aqueous ammonium chloride (75 mL) and allowed to stir for 30 min. The mixture was extracted with ethyl acetate (150 mL), washed with brine (3 x 75 mL) and dried over $MgSO_4$. After filtration the solvent was removed under reduced pressure to yield a liquid which was purified by flash chromatography with an automated chromatography system using a silica flash cartridge applying a heptane/ethyl acetate gradient (from 100% heptane to 100% ethyl acetate in 25 min, 25 mL/min).

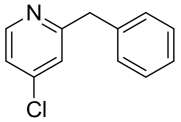

2-Benzyl-4-chloropyridine (**3f**): General procedure C was followed using 2-bromo-4-chloropyridine (0.962 g, 5 mmol). 2-Benzyl-4-chloropyridine (**3f**) was isolated as a faint yellow oil in 58% yield.

**$^1$H-NMR ($CDCl_3$, 400 MHz)** $\delta_H$: 8.44 (d, 1H, *J* = 5.3 Hz), 7.34-7.29 (m, 2H), 7.27-7.21 (m, 3H), 7.15-7.10 (m, 2H), 4.14 (s, 2H).
**$^{13}$C-NMR ($CDCl_3$, 100 MHz)** $\delta_C$: 162.8, 150.2, 144.5, 138.6, 129.2, 128.8, 126.7, 123.4, 121.7, 44.5.
HRMS (ESI) for $C_{12}H_{11}NCl[M+H]^+$, calcd 204.0575, found 204.0582. No spectroscopic data are available in literature.

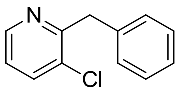

2-Benzyl-3-chloropyridine (**3g**): General procedure C was followed using 2,3-dichloropyridine (0.740 g, 5 mmol). 2-Benzyl-3-chloropyridine (**3g**) was isolated as a faint yellow oil in 91% yield.

**$^1$H-NMR ($CDCl_3$, 400 MHz)** $\delta_H$: 8.46 (dd, 1H, *J* = 4.7, 1.5 Hz), 7.64 (dd, 1H, *J* = 8.0, 1.5 Hz), 7.28 (m, 4H), 7.19 (m, 1H), 7.10 (dd, 1H, *J* = 8.0, 4.7 Hz), 4.13 (s, 2H).
**$^{13}$C-NMR ($CDCl_3$, 100 MHz)** $\delta_C$: 159.3, 148.2, 139.1, 136.3, 129.8, 129.1, 128.8, 126.7, 123.9, 44.1.
HRMS (ESI) for $C_{12}H_{11}NCl[M+H]^+$, calcd 204.0575, found 204.0589. No spectroscopic data are available in literature.

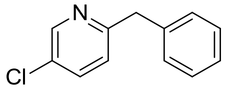

2-Benzyl-5-chloropyridine (**3h**): General procedure C was followed using 2,5-dichloropyridine (0.740 g, 5 mmol). 2-Benzyl-5-chloropyridine (**3h**) was isolated as a faint yellow oil in 81% yield.

**$^{1}$H-NMR (CDCl$_3$, 400 MHz)** $\delta_H$: 8.50 (d, 1H, *J* = 2.4 Hz), 7.54 (dd, 1H, *J* = 8.3, 2.5 Hz), 7.33-7.27 (m, 2H), 7.25-7.19 (m, 3H), 7.05 (d, 1H, *J* = 8.3 Hz), 4.13 (s, 2H).
**$^{13}$C-NMR (CDCl$_3$, 100 MHz)** $\delta_C$: 157.9, 147.5, 138.3, 137.3, 131.5, 129.1, 128.5, 126.5, 122.7, 41.7.
HRMS (ESI) for $C_{12}H_{11}NCl[M+H]^+$, calcd 204.0575, found 204.0584. No spectroscopic data are available in literature.

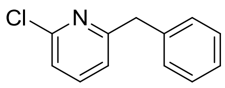

2-Benzyl-6-chloropyridine (**3i**): General procedure C was followed using 2,6-dichloropyridine (0.740 g, 5 mmol). 2-Benzyl-6-chloropyridine (**3i**) was isolated as a faint yellow oil in 51% yield.

**$^{1}$H-NMR (CDCl$_3$, 400 MHz)** $\delta_H$: 7.51 (t, 1H, *J* = 7.7 Hz), 7.34-7.28 (m, 2H), 7.28-7.20 (m, 3H), 7.15 (d, 1H, *J* = 7.9 Hz), 6.98 (d, 1H, *J* = 7.6 Hz), 4.13 (s, 2H).
**$^{13}$C-NMR (CDCl$_3$, 100 MHz)** $\delta_C$: 162.2, 150.8, 139.1, 138.7, 129.3, 128.8, 126.7, 121.8, 121.6, 44.3.
HRMS (ESI) for $C_{12}H_{11}NCl[M+H]^+$, calcd 204.0575, found 204.0582. The reported spectroscopic data are consistent with the available literature data [3].

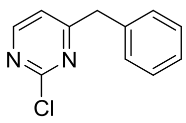

4-Benzyl-2-chloropyrimidine: General procedure C was followed using 2,4-dichloropyrimidine (1.49 g, 10 mmol). 4-Benzyl-2-chloropyrimidine was isolated as a brown oil in 66% yield.

**$^{1}$H-NMR (CDCl$_3$, 400 MHz)** $\delta_H$: 8.45 (d, 1H, *J* = 5.1 Hz), 7.37-7.32 (m, 2H), 7.30-7.24 (m, 3H), 7.00 (d, 1H, *J* = 5.1 Hz), 4.11 (s, 2H).
**$^{13}$C-NMR (CDCl$_3$, 100 MHz)** $\delta_C$: 173.3, 161.3, 159.5, 136.5, 129.4, 129.1, 127.4, 118.9, 44.0.
HRMS (ESI) for $C_{11}H_{10}N_2Cl[M+H]^+$ calcd 205.0527, found 205.0526. No spectroscopic data are available in literature.

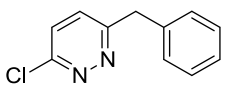

3-Benzyl-6-chloropyridazine: General procedure C was followed using 3,6-dichloropyridazine (2.98 g, 20 mmol). 3-Benzyl-6-chloropyridazine was isolated as a fluffy white solid in 30% yield.

**$^{1}$H-NMR (CDCl$_3$, 400 MHz)** $\delta_H$: 7.40 (d, 1H, *J* = 8.8 Hz), 7.37-7.31 (m, 2H), 7.31-7.25 (m, 3H), 7.24 (d, 1H, *J* = 8.8 Hz), 4.36 (s, 2H).
**$^{13}$C-NMR (CDCl$_3$, 100 MHz)** $\delta_C$: 162.2, 155.4, 137.6, 129.1, 129.0, 128.9, 128.4, 127.1, 41.8.
HRMS (ESI) for $C_{11}H_{10}N_2Cl[M+H]^+$ calcd 205.0527, found 205.0530. Melting point: 92.7 °C. No spectroscopic data are available in literature.

**4. General procedure D (cross coupling) [2]:**

A dry 250 mL Kjeldahl flask, under Ar atmosphere, was charged with 2,2,6,6-tetramethylpiperidine (10.2 mL, 60 mmol) and dry THF (60 mL). After cooling to −20 °C, BuLi (24.0 mL, 2.5 M in hexanes, 60 mmol) was added dropwise. The resulting yellow mixture was stirred for another 1 h, until it reached −10 °C. Then $ZnCl_2$ (33.0 mL, 2.0 M in 2-MeTHF, 66 mmol) was added dropwise. The mixture was stirred for 30 min at −10 °C, followed by 30 min at 25 °C. Afterwards all volatiles were removed under vacuum. Dry THF was added until all orange solid dissolved. Titration of the resulting TMPZnCl·LiCl solution, using salicylaldehyde phenylhydrazone as titrator/indicator, gave a concentration of 0.6–0.8 M.

A dry 50 mL Kjeldahl flask, under Ar atmosphere, was charged with dry THF (10 mL) and 4-methylpyridine (0.64 mL, 6.5 mmol). The TMPZnCl·LiCl solution (15 mL, 0.6–0.8 M in THF, ca. 9.0 mmol) was added dropwise at 25 °C. After stirring for 1 h at 25 °C, palladium(II) trifluoroacetate (0.050 g, 0.15 mmol), S-Phos (0.123 g, 0.30 mmol) and aryl bromide (5.0 mmol) were added. The resulting mixture was stirred for 1 h at 50 °C. After quenching with a mixture of saturated aqueous $NH_4Cl$ and $NH_3$ (10:1, 10 mL), the reaction mixture was extracted three times with ethyl acetate (20 mL). The combined organic fractions were washed with brine (20 mL), dried over $MgSO_4$ and filtered over a pad of Celite®. The solvent was removed under reduced pressure and the resulting residue was finally purified via column chromatography with an automated chromatography system using a silica flash cartridge applying a heptane/ethyl acetate gradient (from 100% heptane to 100% diethyl ether in 25 min, 25 mL/min).

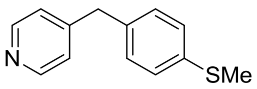


[4-(Methylthio)benzyl]pyridine (**1c**): General procedure D was followed using 4-bromothioanisole (1.015 g, 5.0 mmol). 4-[4-(Methylthio)benzyl]pyridine (**1c**) was isolated as an orange oil in 35% yield.

**$^1$H-NMR (CDCl$_3$, 400 MHz)** $\delta_H$: 8.49 (dd, 2H, $J$ = 4.5, 1.5 Hz), 7.21 (bd, 2H, $J$ = 8.3 Hz), 7.11-7.06 (m, 4H), 3.91 (s, 2H), 2.46 (s, 3H).
**$^{13}$C-NMR (CDCl$_3$, 100 MHz)** $\delta_C$: 149.9, 136.8, 135.8, 129.6, 127.2, 124.1, 40.7, 16.1.
HRMS (ESI) for $C_{13}H_{14}NS$ [M+H]$^+$, calcd 216.0841, found 216.0848. No spectroscopic data are available in literature.

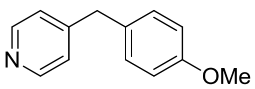


4-(4-Methoxybenzyl)pyridine (**1d**): General procedure D was followed using 4-bromoanisole (0.935 g, 5.0 mmol). 4-(4-Methoxybenzyl)pyridine (**1d**) was isolated as a yellow oil in 67% yield.

**$^1$H-NMR (CDCl$_3$, 400 MHz)** $\delta_H$: 8.48 (dd, 2H, $J$ = 4.4, 1.6 Hz), 7.11-7.06 (m, 4H), 6.85 (bd, 2H, $J$ = 8.7 Hz), 3.90 (s, 2H), 3.78 (s, 3H).
**$^{13}$C-NMR (CDCl$_3$, 100 MHz)** $\delta_C$: 158.5, 150.5, 149.9, 131.0, 130.1, 124.1, 114.2, 55.3, 40.4.
HRMS (ESI) for $C_{13}H_{14}NO$ [M+H]$^+$, calcd 200.1070, found 200.1074. The reported spectroscopic data are consistent with the available literature data [2].

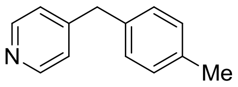


4-(4-Methylbenzyl)pyridine (**1e**): General procedure D was followed using 4-bromotoluene (0.855 g, 5.0 mmol). 4-(4-Methylbenzyl)pyridine (**1e**) was isolated as a faint brown solid in 77% yield.

**$^1$H-NMR (CDCl$_3$, 400 MHz)** $\delta_H$: 8.48 (dd, 2H, $J$ = 4.6, 1.5 Hz), 7.12 (bd, 2H, $J$ = 7.9 Hz), 7.08 (dd, 2H, $J$ = 4.5, 1.5 Hz), 7.06 (bd, 2H, $J$ = 8.0 Hz), 3.92 (s, 2H), 2.33 (s, 3H).
**$^{13}$C-NMR (CDCl$_3$, 100 MHz)** $\delta_C$: 150.4, 149.9, 136.3, 135.9, 129.5, 129.0, 124.2, 40.9, 21.1.
HRMS (ESI) for $C_{13}H_{14}N$ [M+H]$^+$, calcd 184.1121, found 184.1128. Melting point: 53 °C. The reported spectroscopic data are consistent with the available literature data [15].

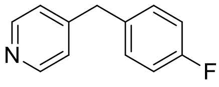


4-(4-Fluorobenzyl)pyridine (**1i**): General procedure D was followed using 4-fluoro-1-bromobenzene (0.875 g, 5.0 mmol). 4-(4-Fluorobenzyl)pyridine (**1i**) was isolated as a yellow oil in 83% yield.

**$^{1}$H-NMR (CDCl$_3$, 400 MHz)** $\delta_H$: 8.50 (dd, 2H, $J$ = 4.4, 1.6 Hz), 7.13 (dd, 2H, $J$ = 8.8, 6.4 Hz), 7.09-7.06 (m, 2H), 7.00 (bt, 2H, $J$ = 8.7 Hz), 3.94 (s, 2H).
**$^{13}$C-NMR (CDCl$_3$, 100 MHz)** $\delta_C$: 161.3 (d, $J_{CF}$ = 245 Hz), 150.0, 149.9, 134.6 (d, $J_{CF}$ = 3.3 Hz), 130.6 (d, $J_{CF}$ = 8.0 Hz), 124.1, 115.6 (d, $J_{CF}$ = 21.4 Hz), 40.4.
HRMS (ESI) for $C_{12}H_{11}FN$ $[M+H]^+$, calcd 188.0870, found 188.0874. The reported spectroscopic data are consistent with the available literature data [16].

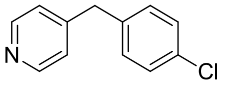


4-(4-Chlorobenzyl)pyridine (**1h**): General procedure D was followed using 4-chloro-1-bromobenzene (0.957 g, 5.0 mmol). 4-(4-Chlorobenzyl)pyridine (**1h**) was isolated as a brown viscous oil in 73% yield.

**$^{1}$H-NMR (CDCl$_3$, 400 MHz)** $\delta_H$: 8.50 (dd, 2H, $J$ = 4.5, 1.6 Hz), 7.28 (bd, 2H, $J$ = 8.4 Hz), 7.10 (bd, 2H, $J$ = 8.4 Hz), 7.07 (bd, 2H, $J$ = 6.0 Hz), 3.93 (s, 2H).
**$^{13}$C-NMR (CDCl$_3$, 100 MHz)** $\delta_C$: 150.0, 149.4, 137.3, 132.6, 130.4, 128.9, 124.1, 40.5.
HRMS (ESI) for $C_{12}H_{11}ClN$ $[M+H]^+$, calcd 204.0575, found 204.0579. No spectroscopic data are available in literature.

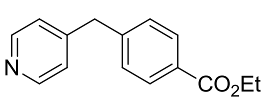


Ethyl 4-(pyridin-4-ylmethyl)benzoate (**1j**): General procedure D was followed using ethyl 4-bromobenzoate (1.145 g, 5.0 mmol). After stirring for 1 h at 25 °C, first $Sc(OTf)_3$ (0.320 g, 0.65 mmol) was added and then after 15 min stirring at 25 °C, the catalyst, ligand and substrate were added. Ethyl 4-(pyridin-4-ylmethyl)benzoate (**1j**) was isolated as a brown oil in 58% yield.

**$^{1}$H-NMR (CDCl$_3$, 400 MHz)** $\delta_H$: 8.51 (dd, 2H, $J$ = 4.6, 1.4 Hz), 8.00 (bd, 2H, $J$ = 8.3 Hz), 7.24 (bd, 2H, $J$ = 8.4 Hz), 7.08 (dd, 2H, $J$ = 4.5, 1.5 Hz), 4.37 (q, 2H, $J$ = 7.1 Hz), 4.01 (s, 2H), 1.38 (t, 3H, $J$ = 7.1 Hz).
**$^{13}$C-NMR (CDCl$_3$, 100 MHz)** $\delta_C$: 166.4, 150.1, 149.1, 144.0, 130.1, 129.1, 129.1, 124.2, 61.0, 41.2, 14.4.
HRMS (ESI) for $C_{15}H_{15}NO_2$ $[M+H]^+$, calcd 242.1176, found 242.1177. The reported spectroscopic data are consistent with the available literature data [2].

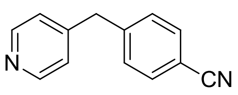


4-(Pyridin-4-ylmethyl)benzonitrile (**1k**): General procedure D was followed using 4-bromobenzonitrile (0.910 g, 5.0 mmol). After stirring for 1 h at 25 °C, first $Sc(OTf)_3$ (0.320 g, 0.65 mmol) was added and then after 15 min stirring at 25 °C, the catalyst, ligand and substrate were added. 4-(Pyridin-4-ylmethyl)benzonitrile (**1k**) was isolated as an orange solid in 82% yield.

**$^{1}$H-NMR (CDCl$_3$, 400 MHz)** $\delta_H$: 8.54 (d, 2H, $J$ = 4.3 Hz), 7.60 (bd, 2H, $J$ = 8.3 Hz), 7.29 (d, 2H, $J$ = 8.5 Hz), 7.07 (d, 2H, $J$ = 5.8 Hz), 4.03 (s, 2H).
**$^{13}$C-NMR (CDCl$_3$, 100 MHz)** $\delta_C$: 150.2, 148.1, 144.4, 132.5, 129.8, 124.1, 118.6, 110.8, 41.2.
HRMS (ESI) for $C_{13}H_{11}N_2$ $[M+H]^+$, calcd 195.0917, found 195.0918. Melting point: 58-61°C. The reported spectroscopic data are consistent with the available literature data [2].

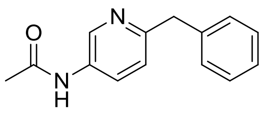


*N*-(6-Benzylpyridin-3-yl)acetamide (**3e**): 6-benzylpyridin-3-amine (0.737 g, 4 mmol) was dissolved in 20 mL of acetone and triethylamine (0.726 mL, 5.2 mmol) was added, followed by the dropwise addition of acetyl chloride (0.370 ml, 5.2 mmol) in acetone (5 mL). The reaction mixture was stirred for 3 h, after which it was quenched by the addition of water (5 mL) and the solvent was removed by evaporation. The residue was taken up in $CH_2Cl_2$ (30 mL) and

the obtained solution was washed with water (30 mL). The organic layer was dried with magnesium sulfate, filtered, and the solvent was evaporated. After purification by column chromatography *N*-(6-Benzylpyridin-3-yl)acetamide (**3e**) was isolated as a white solid in a 46% yield.

**$^{1}$H-NMR ($CDCl_3$, 400 MHz)** $\delta_H$: 8.44 (d, 1H, *J* = 2.5 Hz), 8.03 (dd, 1H, *J* = 8.4, 2.6 Hz), 7.78 (bs, 1H), 7.31-7.25 (m, 2H), 7.24-7.17 (m, 3H), 7.08 (d, 1H, *J* = 8.4 Hz), 4.10 (s, 2H), 2.14 (s, 3H).
**$^{13}$C-NMR ($CDCl_3$, 100 MHz)** $\delta_C$: 168.9, 156.6, 140.7, 139.5, 132.9, 129.1, 128.7, 128.3, 126.5, 123.2, 44.1, 24.4.
HRMS (ESI) for $C_{14}H_{15}N_2O[M+H]^+$, calcd 227.1179, found 227.1176. Melting point: 168 °C. No spectroscopic data are available in literature.

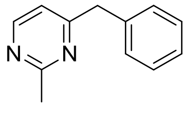


4-Benzyl-2-methylpyrimidine (**5f**): To a flame-dried flask under argon containing: $Pd(PPh_3)_4$ (0.757 g, 0.0655 mmol), 4-benzyl-2-chloropyrimidine (1.340 g, 6.55 mmol) and 50 mL of dry THF was added trimethylaluminum (6.55 mL, 2 M in hexane, 13.10 mmol) via a syringe. The mixture was stirred at 75 °C for 18 h. After completion 20 mL of methanol were added very slowly to quench the reaction, followed by 50 mL of water. The reaction mixture was extracted with ethyl acetate (3 x 50 mL), the combined organic fractions were washed with brine (3 x 75 mL) and dried over magnesium sulfate. The solvent was removed by rotary evaporation to yield a brown liquid which was purified by flash chromatography applying a heptane/ethyl acetate gradient. 4-Benzyl-2-methylpyrimidine (**5f**) was isolated as a brown oil in 68% yield.

**$^{1}$H-NMR ($CDCl_3$, 400 MHz)** $\delta_H$: 8.47 (d, 1H, *J* = 5.2 Hz), 7.36-7.30 (m, 2H), 7.28-7.23 (m, 3H), 6.85 (d, 1H, *J* = 5.2 Hz), 4.08 (s, 2H), 2.73 (s, 3H).
**$^{13}$C-NMR ($CDCl_3$, 100 MHz)** $\delta_C$: 169.5, 168.0, 157.1, 137.6, 129.3, 128.9, 127.0, 117.3, 44.3, 26.2.
HRMS (ESI) for $C_{12}H_{13}N_2[M+H]^+$ calcd 185.1073, found 185.1072. No spectroscopic data are available in literature.

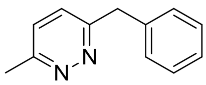


3-Benzyl-6-methylpyridazine (**5g**): To a flame-dried flask under argon containing: $Pd(PPh_3)_4$ (0.689 g, 0.596 mmol), 3-benzyl-6-chloropyridazine (1.22 g, 5.96 mmol) and 50 mL of dry THF was added trimethylaluminium (6.56 ml, 2 M in hexane, 13.11 mmol) via syringe. The mixture was stirred at 75 °C for 18 h. After completion 20 mL of methanol was added very slowly to quench the reaction, followed by 50 mL of water. The reaction mixture was extracted with ethyl acetate (3 x 50 mL), the combined organic fractions were washed with brine (3 x 75 mL) and dried over magnesium sulfate. The solvent was removed by rotary evaporation to yield a brown liquid which was purified by flash chromatography applying a heptane/ethyl acetate gradient. 3-Benzyl-6-methylpyridazine (**5g**) was isolated as a brown solid in 69% yield.

**$^{1}$H-NMR ($CDCl_3$, 400 MHz)** $\delta_H$: 7.34-7.20 (m, 5H), 7.18 (d, 1H, *J* = 8.6 Hz), 7.12 (d, 1H, *J* = 8.6 Hz), 4.31 (s, 2H), 2.66 (s, 3H).
**$^{13}$C-NMR ($CDCl_3$, 100 MHz)** $\delta_C$: 160.4, 158.1, 138.5, 129.1, 128.8, 127.1, 126.7, 126.5, 42.2, 22.0.
HRMS (ESI) for $C_{12}H_{13}N_2[M+H]^+$ calcd 185.1073, found 185.1079. Melting point: 50 °C. No spectroscopic data are available in literature.

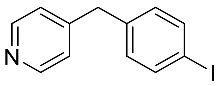


4-(4-Iodobenzyl)pyridine (**1f**): A 100 mL three-necked round-bottomed flask was charged with 4-(pyridin-4-ylmethyl)aniline (2.0 g, 11.0 mmol) and HCl (10 mL, 6 M in water). The mixture was cooled to 0 °C before a solution of sodium nitrite (1.15 g,

16.6 mmol) in water (5 mL) was added dropwise, maintaining the temperature below 5 °C. Subsequently a solution of potassium iodide (4.6 g, 27.7 mmol) in water (5 mL) was added, maintaining the temperature below 20 °C. The resulting mixture was stirred for 30 min at rt and for another 30 min at 60 °C. After cooling to rt, the mixture was made alkaline by the addition of NaOH (5 M in water) and extracted with dichloromethane (3 x 50 mL). The combined organic fractions were washed with sodium bisulfite solution (50 mL, sat.), dried over $MgSO_4$ and filtered over a pad of Celite®. The solvent was removed under reduced pressure and the resulting residue was finally purified via column chromatography with an automated chromatography system using a silica flash cartridge applying a heptane/ethyl acetate gradient (from 100% heptane to 100% ethyl acetate in 25 min, 25 mL/min). 4-(4-Iodobenzyl)pyridine (**1f**) was isolated as an off-white solid in 38% yield.

**$^1$H-NMR ($CDCl_3$, 400 MHz)** $\delta_H$: 8.50 (dd, 2H, *J* = 4.5, 1.4 Hz), 7.64 (bd, 2H, *J* = 8.3 Hz), 7.07 (dd, 2H, *J* = 4.3, 1.4 Hz), 6.92 (bd, 2H, *J* = 8.3 Hz), 3.90 (s, 2H).
**$^{13}$C-NMR ($CDCl_3$, 100 MHz)** $\delta_C$: 150.0, 149.4, 138.6, 137.9, 131.1, 124.2, 92.1, 40.8.
HRMS (ESI) for $C_{12}H_{11}IN$ $[M+H]^+$, calcd 295.9931, found 295.9940. mp: 64-68 °C. The reported spectroscopic data are consistent with the available literature data [15].

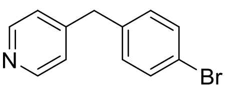


4-(4-Bromobenzyl)pyridine (**1g**): A 100 mL three-necked round-bottomed flask was charged with 4-(pyridin-4-ylmethyl)aniline (0.860 g, 4.67 mmol) and HBr (10 mL, 6 M in water). The mixture was cooled to 0 °C before a solution of sodium nitrite (0.450 g, 6.52 mmol) in water (5 mL) was added dropwise, maintaining the temperature below 5 °C. Meanwhile another 100 mL three-necked round-bottomed flask was charged with CuBr (2.0 g, 13.9 mmol), HBr (5 mL, 48 wt % in water) and water (5 mL). The diazonium salt solution was added dropwise to the latter flask, maintaining the temperature below 20 °C. The resulting mixture was stirred for 1 h between 0 °C and 60 °C. After cooling to room temperature, the mixture was made alkaline by the addition of NaOH (5 M in water) and extracted with dichloromethane (3 x 50 mL). The combined organic fractions were washed twice with ammonium hydroxide solution (50 mL, 28–30 wt % in water), once with brine (50 mL), dried over $MgSO_4$ and filtered over a pad of Celite®. The solvent was removed under reduced pressure and the resulting residue was finally purified via column chromatography with an automated chromatography system using a silica flash cartridge applying a heptane/ethyl acetate gradient (from 100% heptane to 100% ethyl acetate in 25 min, 25 mL/min). 4-(4-Bromobenzyl)pyridine (**1g**) was isolated as a faint yellow oil in 70% yield.

**$^1$H-NMR ($CDCl_3$, 400 MHz)** $\delta_H$: 8.50 (dd, 2H, *J* = 4.5, 1.5 Hz), 7.44 (bd, 2H, *J* = 8.4 Hz), 7.07 (dd, 2H, *J* = 4.5, 1.5 Hz), 7.04 (bd, 2H, *J* = 8.5 Hz), 3.92 (s, 2H).
**$^{13}$C-NMR ($CDCl_3$, 100 MHz)** $\delta_C$: 150.1, 149.3, 137.9, 131.9, 130.8, 124.1, 120.7, 40.7.
HRMS (ESI) for $C_{12}H_{11}BrN$ $[M+H]^+$, calcd 248.0069, found 248.0074. The reported spectroscopic data are consistent with the available literature data [17].

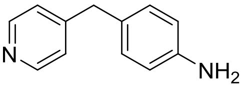


4-(Pyridin-4-ylmethyl)aniline (**1b**): A 500 mL Parr bottle was charged with 4-(4-nitrobenzyl)pyridine (6.9 g, 32.2 mmol), EtOH (100 mL) and flushed thoroughly with Ar before adding palladium on carbon (0.7 g, 10 wt %). The resulting mixture was shaken under $H_2$ atmosphere (50 psi) at rt for 1.5 h, then filtered over a pad of Celite® and rinsed with EtOH (50 mL).The solvent was removed under reduced pressure and the resulting residue was finally recrystallized from ethyl acetate. 4-(Pyridin-4-ylmethyl)aniline (**1b**) was isolated as a white solid in 84% yield.

**$^{1}$H-NMR (CDCl$_3$, 400 MHz)** $\delta_H$: 8.46 (dd, 2H, *J* = 4.4, 1.6 Hz), 7.07 (dd, 2H, *J* = 4.4, 1.5 Hz), 6.94 (bd, 2H, *J* = 8.4 Hz), 6.62 (bd, 2H, *J* = 8.4 Hz), 3.84 (s, 2H), 3.63 (bs, 2H).
**$^{13}$C-NMR (CDCl$_3$, 100 MHz)** $\delta_C$: 150.9, 149.8, 145.1, 129.9, 128.8, 124.1, 115.4, 40.4.
HRMS (ESI) for $C_{12}H_{13}N_2$ [M+H]$^+$, calcd 185.1073, found 185.1073. mp: 158-160 °C. The reported spectroscopic data are consistent with the available literature data [15].

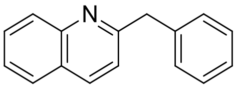

2-Benzylquinoline (**5a**): A dry 50 mL Kjeldahl flask, under Ar atmosphere, was charged with dry THF (10 mL) and 2-methylquinoline (0.90 mL, 6.65 mmol). A TMPZnCl·LiCl solution (8 mL, 1.2 M in THF, 9.5 mmol) was added dropwise at 25 °C. After stirring for 1 h at 25 °C, palladium(II) acetate (0.034 g, 0.15 mmol), XantPhos (0.176 g, 0.30 mmol) and bromobenzene (0.785 g, 5.0 mmol) were added. The resulting mixture was stirred for 1 h at 50 °C. After quenching with a mixture of saturated aqueous $NH_4Cl$ and $NH_3$ (10:1, 10 mL), the reaction mixture was extracted three times with ethyl acetate (20 mL). The combined organic fractions were washed with brine (20 mL), dried over $MgSO_4$ and filtered over a pad of Celite®. The solvent was removed under reduced pressure and the resulting residue was finally purified via column chromatography with an automated chromatography system using a silica flash cartridge applying a heptane/ethyl acetate gradient (from 100% heptane to 100% ethyl acetate in 25 min, 25 mL/min). 2-Benzylquinoline (**5a**) was isolated as a yellow oil in 61% yield.

**$^{1}$H-NMR (CDCl$_3$, 400 MHz)** $\delta_H$: 8.09 (d, 1H, *J* = 8.5 Hz), 8.02 (d, 1H, *J* = 8.5 Hz), 7.76 (bd, 1H, *J* = 8.1 Hz), 7.70 (ddd, 1H, *J* = 8.4, 7.0, 1.3 Hz), 7.49 (bt, 1H, *J* = 7.5 Hz), 7.33-7.28 (m, 4H), 7.24-7.19 (m, 2H), 4.35 (s, 2H).
**$^{13}$C-NMR (CDCl$_3$, 100 MHz)** $\delta_C$: 161.3, 147.9, 139.3, 136.6, 129.6, 129.3, 129.1, 128.7, 127.6, 126.9, 126.6, 126.1, 121.6, 45.7.
HRMS (ESI) for $C_{16}H_{14}N$ [M+H]$^+$, calcd 220.1121, found 220.1128. The reported spectroscopic data are consistent with the available literature data [18].

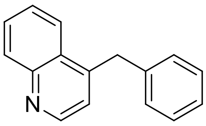

4-Benzylquinoline (**5b**): A dry 50 mL Kjeldahl flask, under Ar atmosphere, was charged with dry THF (10 mL) and 4-methylquinoline (0.90 mL, 6.65 mmol). A TMPZnCl·LiCl solution (10 mL, 1.0 M in THF, 10.0 mmol) was added dropwise at 25 °C. After stirring for 1 h at 25 °C, palladium(II) acetate (0.034 g, 0.15 mmol), XantPhos (0.176 g, 0.30 mmol) and bromobenzene (0.785 g, 5.0 mmol) were added. The resulting mixture was stirred for 1 h at 50 °C. After quenching with a mixture of saturated aqueous $NH_4Cl$ and $NH_3$ (10:1, 10 mL), the reaction mixture was extracted three times with ethyl acetate (20 mL). The combined organic fractions were washed with brine (20 mL), dried over $MgSO_4$ and filtered over a pad of Celite®. The solvent was removed under reduced pressure and the resulting residue was finally purified via column chromatography with an automated chromatography system using a silica flash cartridge applying a heptane/ethyl acetate gradient (from 100% heptane to 100% ethyl acetate in 25 min, 25 mL/min). 4-Benzylquinoline (**5b**) was isolated as an orange oil in 85% yield.

**$^{1}$H-NMR (CDCl$_3$, 400 MHz)** $\delta_H$: 8.82 (d, 1H, *J* = 4.4 Hz), 8.13 (d, 1H, *J* = 8.4 Hz), 8.03 (d, 1H, *J* = 8.4 Hz), 7.69 (ddd, 1H, *J* = 8.3, 6.9, 1.3 Hz), 7.52 (ddd, 1H, *J* = 8.2, 7.1, 1.0 Hz), 7.33-7.17 (m, 5H), 7.13 (d, 1H, *J* = 4.4 Hz), 4.44 (s, 2H).
**$^{13}$C-NMR (CDCl$_3$, 100 MHz)** $\delta_C$: 150.4, 148.5, 146.6, 138.7, 130.3, 129.2, 129.0, 128.8, 127.7, 126.7, 126.6, 123.9, 121.9, 38.2. HRMS (ESI) for $C_{16}H_{14}N$ [M+H]$^+$, calcd 220.1121, found 220.1126. No spectroscopic data are available in literature.

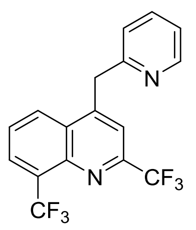

4-(Pyridin-2-ylmethyl)-2,8-bis(trifluoromethyl)quinoline (**12**) [3]: A 50 mL round-bottomed flask was subsequently charged with palladium(II) trifluoroacetate (0.055 g, 0.167 mmol), 4-chloro-2,8-bis(trifluoromethyl)quinoline (**7b**, 1.00 g, 3.34 mmol), 2,4-dimethyl-3-(pyridin-2-ylmethyl)pentan-3-ol (**11**, 0.830 g, 4.0 mmol), $Cs_2CO_3$ (1.631 g, 5.0 mmol), *o*-xylene (10 mL) and tricyclohexylphosphine (0.53 mL, 20 wt % in toluene, 0.334 mmol). The resulting mixture was flushed with $N_2$ for 5 min and stirred at reflux under a $N_2$ atmosphere for 6 h. After cooling to rt, the mixture was filtered over a pad of Celite® (dichloromethane, 50 mL). The solvent was removed under reduced pressure and the crude residue was finally purified via column chromatography with an automated chromatography system using a silica flash cartridge applying dichloromethane (100%) as eluent, 25 mL/min. 4-(Pyridin-2-ylmethyl)-2,8-bis(trifluoromethyl)quinoline (**12**) was isolated as a faint brown viscous oil in 63% yield.

**$^1$H-NMR ($CDCl_3$, 400 MHz)** $\delta_H$: 8.57 (bd, 1H, *J* = 4.8 Hz), 8.41 (bd, 1H, *J* = 8.4 Hz), 8.13 (bd, 1H, *J* = 7.2 Hz), 7.71 (s, 1H), 7.67 (t, 1H, *J* = 7.9 Hz), 7.61 (td, 1H, *J* = 7.7, 1.8 Hz), 7.18 (dd, 1H, *J* = 7.5, 5.0 Hz), 7.11 (bd, 1H, *J* = 7.8 Hz), 4.70 (s, 2H).
**$^{13}$C-NMR ($CDCl_3$, 100 MHz)** $\delta_C$: 157.8, 149.9, 148.4, 148.3 (q, $J_{CF}$ = 35 Hz), 144.0, 137.0, 129.4 (q, $J_{CF}$ = 30 Hz), 128.9 (q, $J_{CF}$ = 5.5 Hz), 128.8, 128.8, 127.2, 123.6 (q, $J_{CF}$ = 273 Hz), 123.2, 122.1, 121.2 (q, $J_{CF}$ = 275 Hz), 118.6 (q, $J_{CF}$ = 2.0 Hz), 41.8.
HRMS (ESI) for $C_{17}H_{11}F_6N_2$ $[M+H]^+$, calcd 357.0821, found 357.0823. No spectroscopic data are available in literature.

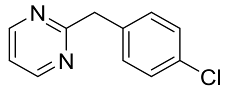

2-(4-Chlorobenzyl)pyrimidine (**5c**) [3]: A 50 mL round-bottomed flask was subsequently charged with palladium(II) trifluoroacetate (0.033 g, 0.10 mmol), 4-chlorobromobenzene (0.383 g, 2.0 mmol), 2,4-dimethyl-3-(pyrimidin-2-ylmethyl)pentan-3-ol (0.417 g, 2.0 mmol), $Cs_2CO_3$ (0.782 g, 2.4 mmol), toluene (6 mL) and tricyclohexylphosphine (0.3 mL, 20 wt % in toluene, 0.19 mmol). The resulting mixture was flushed with $N_2$ for 5 min and stirred at reflux under a $N_2$ atmosphere for 4 h. After cooling to rt, the mixture was filtered over a pad of Celite® (dichloromethane, 20 mL). The solvent was removed under reduced pressure and the crude residue was finally purified via column chromatography with an automated chromatography system using a silica flash cartridge applying a heptane/ethyl acetate gradient (from 100% heptane to 100% ethyl acetate in 25 min, 25 mL/min). 2-(4-Chlorobenzyl)pyrimidine (**5c**) was isolated as an orange oil in 69% yield.

**$^1$H-NMR ($CDCl_3$, 400 MHz)** $\delta_H$: 8.67 (d, 2H, *J* = 4.9 Hz), 7.33-7.23 (m, 4H), 7.12 (t, 1H, *J* = 4.9 Hz), 4.25 (s, 2H).
**$^{13}$C-NMR ($CDCl_3$, 100 MHz)** $\delta_C$: 169.6, 157.3, 136.7, 132.5, 130.5, 128.6, 118.8, 45.3.
HRMS (ESI) for $C_{11}H_{10}ClN_2$ $[M+H]^+$, calcd 205.0527, found 205.0532. The reported spectroscopic data are consistent with the available literature data [19].

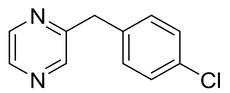

2-(4-Chlorobenzyl)pyrazine (**5d**) [3]: A 100 mL round-bottomed flask was subsequently charged with palladium(II) trifluoroacetate (0.083 g, 0.25 mmol), 4-chlorobromobenzene (0.957 g, 5.0 mmol), 2,4-dimethyl-3-(pyrazin-2-ylmethyl)pentan-3-ol (1.25 g, 6.0 mmol), $Cs_2CO_3$ (1.955 g, 6.0 mmol), toluene (15 mL) and tricyclohexylphosphine (0.8 mL, 20 wt % in toluene, 0.50 mmol). The resulting mixture was flushed with $N_2$ for 5 min and stirred at reflux under a $N_2$ atmosphere for 4 h. After cooling to rt, the mixture was filtered over a pad of Celite® (dichloromethane, 50 mL). The solvent was removed under reduced pressure and the crude residue was finally purified via column chromatography with an automated chromatography system using a silica flash cartridge applying a heptane/ethyl acetate gradient (from 100% heptane to 100% ethyl acetate in 25 min, 25 mL/min). 2-(4-Chlorobenzyl)pyrazine (**5d**) was isolated as a faint yellow solid in 64% yield.

**$^{1}$H-NMR (CDCl$_3$, 400 MHz)** $\delta_H$: 8.52-8.40 (m, 3H), 7.29 (bd, 2H, *J* = 8.4 Hz), 7.20 (bd, 2H, *J* = 8.4 Hz), 4.13 (s, 2H).

**$^{13}$C-NMR (CDCl$_3$, 100 MHz)** $\delta_C$: 156.0, 144.8, 144.3, 142.7, 136.7, 132.8, 130.4, 129.0, 41.3.

HRMS (ESI) for $C_{11}H_{10}ClN_2$ $[M+H]^+$, calcd 205.0527, found 205.0530. mp: 48-52 °C. The reported spectroscopic data are consistent with the available literature data [20].

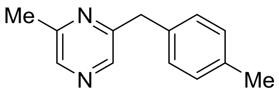


6-(4-Methylbenzyl)-2-methylpyrazine (**18a**) [3]: A 100mL round-bottomed flask was subsequently charged with palladium(II) trifluoroacetate (0.083 g, 0.25 mmol), 4-chlorotoluene (0.633 g, 5.0 mmol), 2,4-dimethyl-3-[(6-methylpyrazin-2-yl)methyl]pentan-3-ol (1.112 g, 5.0 mmol), $Cs_2CO_3$ (1.955 g, 6.0 mmol), *o*-xylene (15 mL) and tricyclohexylphosphine (0.80 mL, 20 wt % in toluene, 0.50 mmol). The resulting mixture was flushed with $N_2$ for 5 min and stirred at reflux under a $N_2$ atmosphere for 6 h. After cooling to rt, the mixture was filtered over a pad of Celite® (dichloromethane, 30 mL). The solvent was removed under reduced pressure and the crude residue was finally purified via column chromatography with an automated chromatography system using a silica flash cartridge applying a heptane/ethyl acetate gradient (from 100% heptane to 100% ethyl acetate in 25 min, 25 mL/min). 6-(4-Methylbenzyl)-2-methylpyrazine (**18a**) was isolated as a yellow oil in 72% yield.

**$^{1}$H-NMR (CDCl$_3$, 400 MHz)** $\delta_H$: 8.26 (s, 1H), 8.21 (s, 1H), 7.17-7.07 (m, 4H), 4.08 (s, 2H), 2.52 (s, 3H), 2.30 (s, 3H).

**$^{13}$C-NMR (CDCl$_3$, 100 MHz)** $\delta_C$: 155.4, 152.9, 141.8, 141.4, 136.1, 135.2, 129.3, 128.8, 41.5, 21.5, 20.9.

HRMS (ESI) for $C_{13}H_{15}N_2$ $[M+H]^+$, calcd 199.1230, found 199.1235. No spectroscopic data are available in literature.

## III. ICP-MS analysis results

Fe and Cu impurities in the reaction products were determined by ICP-MS analysis. A sample of the reaction product was taken and destroyed before analysis. Blank samples underwent the same procedure but contained no organic material.

The destruction was performed by treating the sample with $H_2SO_4$ (5 mL) and $H_2O_2$ (5 mL) and stirring at 90 °C for 20 min followed by stirring 120 °C until a clear solution was formed. Next 2 mL of HCL (30%) is added and the mixture is diluted with Milli-Q water to a volume of 50 mL. ICP-MS analysis is performed on this solution. All glassware used should be properly cleaned using a 10% solution of $HNO_3$.

For the assessement of the purification method on papaveraldine (**15**) the extraction and chromatography procedure described in general procedure A (oxidation) was used. The crystallization method described in reference [21] was used to recrystallize the product.

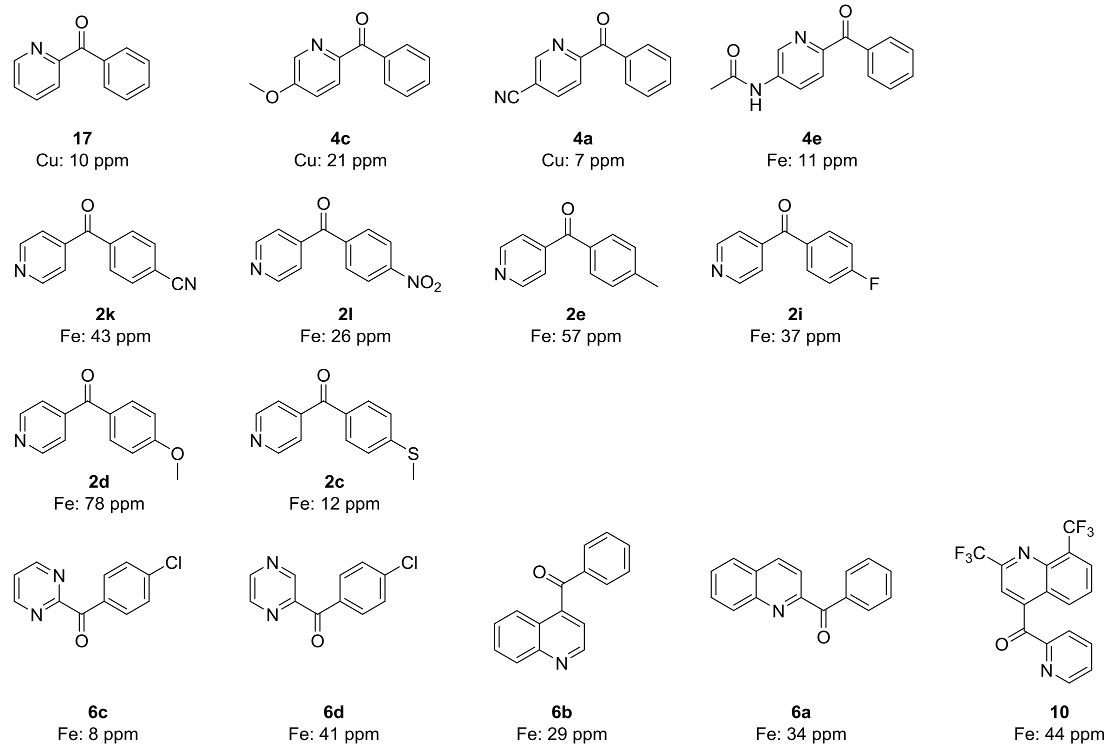


**Figure S1.** Results of the trace-metal analysis using ICP-MS of a representative set of molecules. Blank samples for Cu analysis provided an average 0.19 ppm of Cu, blank samples for Fe analysis provided average 1.37 ppm of Fe.

## IV. In situ IR reaction monitoring

Infrared reaction monitoring experiments were performed on a Matrix-MF and a ReactIR spectrometer using respectively a Diamond ATR fiber probe (IN355, Ø 6.3 mm) and a custom made (DST, Ø 6.35 mm) DiComp AgX probe. The initial reaction rate was determined by following the formation of 2-benzoylpyridine (**17**) over time. More specifically, the carbonyl C=O stretch of the ketone was chosen as a viable peak for integration due to the fact that it is intense and has no overlap with peaks of other reaction components. Integration was executed for the full area under the curve between 1653 $cm^{-1}$ and 1685 $cm^{-1}$.

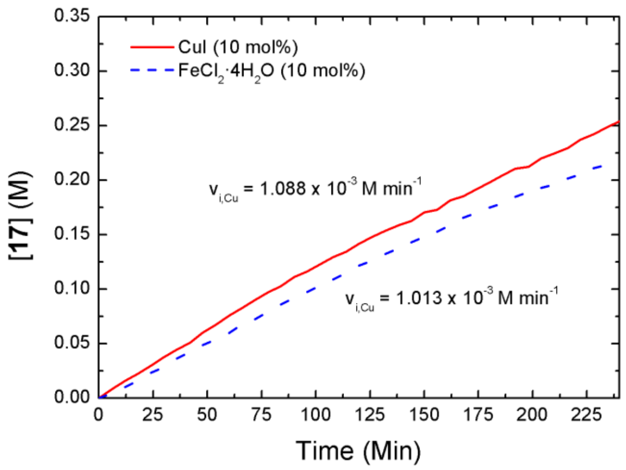


**Figure S2**: Comparison between Cu and Fe catalysis. Reaction conditions: 2-benzylpyridine (**16**, 5 mmol), CuI (10 mol %) or $FeCl_2·4H_2O$ (10 mol %), AcOH (1 equiv), $O_2$ (balloon), DMSO (10 mL), 100 °C.

## V. DFT calculation of equilibrium constants

To calculate the desired equilibrium constants, the geometries of the different conformations of both the imine and the enamine-form were optimized at the B3PW91/aug-cc-pVDZ level of theory. Due to the rather large dipole moment of the molecules and the difference in dipole moment of the imine and the respective enamine tautomer (Table S2), the self-consistent reaction field (scrf) model was used to account for solvent–solute interactions with DMSO. The Gibbs free energies of all conformers were calculated and the most stable imine and respective enamine conformer were used to calculate the equilibrium constant $K_{eq}$ and p$K_{eq}$ as follows:

$$\Delta G° = G°_{enamine} - G°_{imine}$$

$$K_{eq} = \exp\left(-\frac{\Delta G°}{RT}\right)$$

$$pK_{eq} = -\log K_{eq}$$

with the temperature $T$ = 298.15 K and ideal gas constant $R$ = 8.31 $Jmol^{-1}K^{-1}$. For all calculations the Gaussian09 program was used [22].

In the mechanistic study of this oxidation reaction it was established that the reaction is initiated by an imine–enamine tautomerization [23]. This tautomerization can be quantified by an equilibration constant (p$K_{eq}$) which is a thermodynamical parameter. Empirically we found that substrates with a calculated p$K_{eq}$ value smaller than 15 could be oxidized by this method. This is also exemplified by substrate **3i** which has a calculated p$K_{eq}$ of 15.2 (Table S1) and indeed could not be oxidized under the standard conditions (Table 2, entries 23 and 24). While this parameter provides a tool to predict whether or not a substrate can be oxidized using this method it provides no explanation for the incomplete conversion of several pyridine substituted substrates (notably **3a**, **3c**, **3g** and **3h**). The explanation for these low conversions lies in the basicity of the pyridine nitrogen since the first step of the tautomerization is the protonation of the pyridine nitrogen by the added acid (AcOH).

**Table S1:** Calculated $K_{eq}$ and p$K_{eq}$.

| **Molecule** | **substrate** | $K_{eq}$ | p$K_{eq}$ |
|---|---|---|---|
| **1b** | 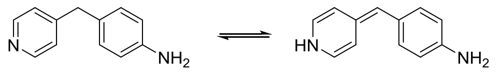 | 3.43E-12 | 11.5 |
| **1c** | 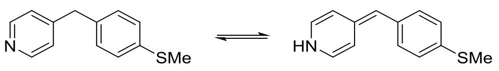 | 2.51E-11 | 10.6 |
| **1d** | 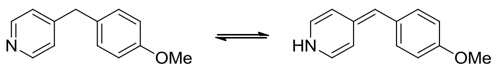 | 4.55E-12 | 11.3 |
| **1e** | 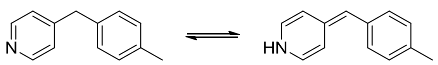 | 6.45E-12 | 11.2 |
| **1f** | 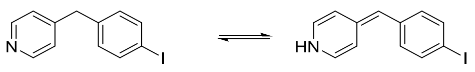 | 5.97E-11 | 10.2 |
| **1g** | 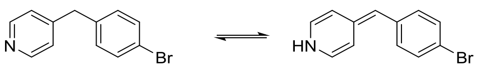 | 4.28E-11 | 10.4 |
| **1h** | 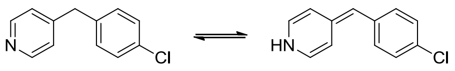 | 5.45E-11 | 10.3 |
| **1i** | 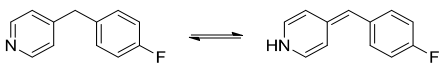 | 1.15E-11 | 10.9 |
| **1j** | 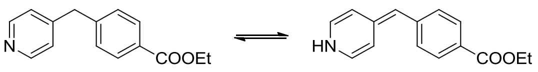 | 8.14E-10 | 9.1 |
| **1k** | 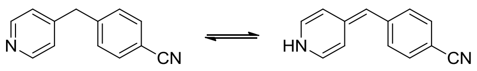 | 5.31E-09 | 8.3 |

| | | | |
|---|---|---|---|
| **1l** | 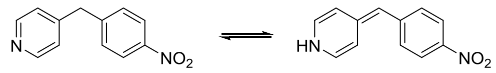 | 1.95E-07 | 6.7 |
| **3a** | 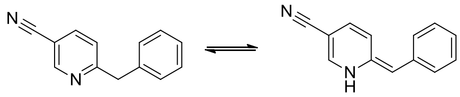 | 1.37E-09 | 8.9 |
| **3b** | 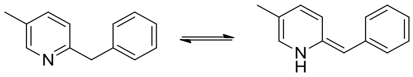 | 4.83E-13 | 12.3 |
| **3c** | 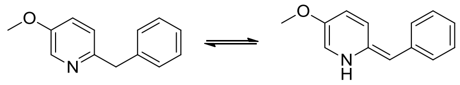 | 9.45E-15 | 14.0 |
| **3d** | 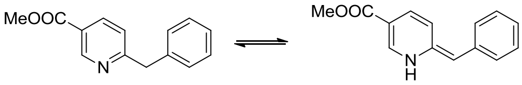 | 2.56E-09 | 8.6 |
| **3e** | 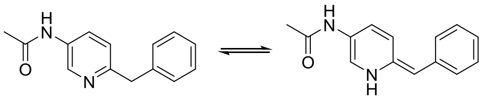 | 2.15E-12 | 11.7 |
| **3f** | 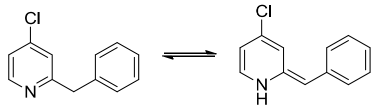 | 2.10E-12 | 11.7 |
| **3g** | 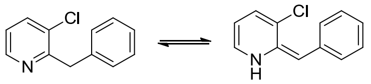 | 7.23E-12 | 11.1 |
| **3h** | 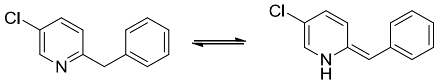 | 1.55E-13 | 12.8 |
| **3i** | 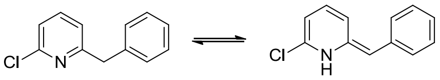 | 6.69E-16 | 15.2 |
| **5a** | 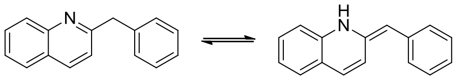 | 8.43E-08 | 7.1 |
| **5b** | 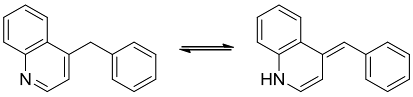 | 4.21E-08 | 7.4 |
| **5c** | 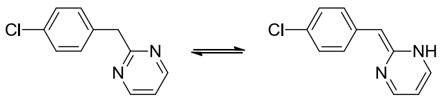 | 9.31E-12 | 11.0 |
| **5d** | 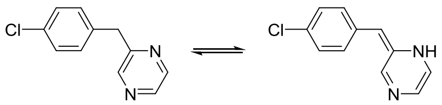 | 2.56E-11 | 10.6 |
| **5f** | 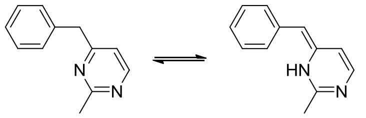 | 1.07E-09 | 9.0 |
| **5g** | 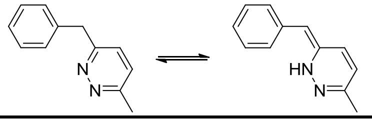 | 7.24E-10 | 9.1 |

| | | | |
|---|---|---|---|
| **15** | 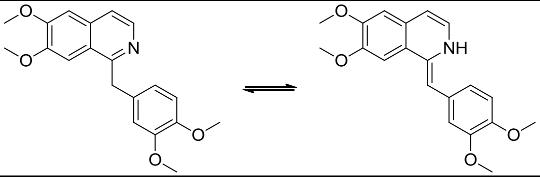 | 5.99E-13 | 12.2 |

**Table S2:** Calculated thermodynamical values and dipole moments.

| **Entry*** | **$\Delta H$ (kJ mol$^{-1}$)** | **$\Delta S$ (kJ mol$^{-1}$)** | **$\Delta G$ (kJ mol$^{-1}$)** | **$\mu_{imine}$ (D)** | **$\mu_{enamine}$ (D)** |
|---|---|---|---|---|---|
| **1b** | 42.3 | 0.001 | 42.1 | 3.6 | 5.5 |
| **1c** | 35.8 | -0.015 | 40.4 | 2.7 | 2.0 |
| **1d** | 60.0 | -0.016 | 64.7 | 4.7 | 7.1 |
| **1e** | 58.1 | -0.019 | 63.8 | 4.0 | 6.6 |
| **1f** | 46.4 | -0.019 | 52.1 | 5.5 | 3.5 |
| **1g** | 70.8 | -0.008 | 73.2 | 5.5 | 1.5 |
| **1h** | 53.9 | -0.015 | 58.6 | 3.4 | 10.1 |
| **1i** | 57.0 | -0.018 | 62.4 | 3.3 | 9.0 |
| **1j** | 45.5 | -0.021 | 51.9 | 4.7 | 12.8 |
| **1k** | 42.0 | -0.018 | 47.2 | 5.8 | 16.5 |
| **1l** | 55.3 | -0.012 | 58.9 | 3.9 | 4.6 |
| **3a** | 46.9 | -0.012 | 50.6 | 5.7 | 5.5 |
| **3b** | 63.6 | -0.022 | 70.3 | 3.3 | 4.4 |
| **3c** | 72.8 | -0.024 | 80.0 | 2.1 | 4.9 |
| **3d** | 44.5 | -0.015 | 49.0 | 0.3 | 4.0 |
| **3e** | 61.8 | -0.016 | 66.6 | 6.6 | 8.8 |
| **3f** | 59.8 | -0.023 | 66.6 | 0.7 | 3.3 |
| **3g** | 61.0 | -0.008 | 63.6 | 2.2 | 4.7 |
| **3h** | 68.5 | -0.016 | 73.1 | 2.8 | 1.0 |
| **3i** | 81.2 | -0.018 | 86.6 | 4.6 | 2.3 |
| **5a** | 35.8 | -0.015 | 40.4 | 2.7 | 2.0 |
| **5b** | 42.3 | 0.001 | 42.1 | 3.6 | 5.5 |
| **5c** | 81.3 | -0.014 | 85.5 | 1.2 | 5.1 |

| | | | | | |
|---|---|---|---|---|---|
| **5d** | 56.1 | -0.014 | 60.4 | 2.4 | 3.0 |
| **5f** | 40.2 | -0.037 | 51.2 | 2.6 | 8.9 |
| **5g** | 46.4 | -0.019 | 52.1 | 5.5 | 3.5 |
| **15** | 69.2 | -0.002 | 69.7 | 4.1 | 3.4 |

For molecule **15** (paparavine) a conformational analysis for both the imine and the enamine tautomers was performed. Two different programs (Spartan, ComputeVOA) were used that explore the conformational space based on molecular force fields. The conformers were optimized at the B3PW91/cc-pVDZ level of theory. The imine (tot# 147) and enamine (tot# 93) conformers with the lowest energy were used as input for the calculations at the PCM/B3PW91/aug-cc-pVDZ level of theory, described above.

# VI. References


[1] Krapcho, A. P.; Ellis, M. *Arkivoc* **2000**, *1*, 43-50.

[2] Duez, S.; Steib, A. K.; Manolikakes, S. M.; Knochel, P. *Angew. Chem. Int. Ed.* **2011**, *50*, 7686-7690.

[3] Niwa, T.; Yorimitsu, H.; Oshima, K. *Angew. Chem. Int. Edit.* **2007**, *46*, 2643-2645.

[4] Busto, E.; Gotor-Fernández, V.; Gotor, V. *Tetrahedron: Asymmetry* **2006**, *17*, 1007-1016.

[5] Choppin, S.; Gros, P.; Fort, Y. *Eur. J. Org. Chem.* **2001**, *2001*, 603-606.

[6] Maerten, E.; Sauthier, M.; Mortreux, A.; Castanet, Y. *Tetrahedron* **2007**, *63*, 682-689.

[7] Awuah, E.; Capretta, A. *J. Org. Chem.* **2010**, *75*, 5627-5634.

[8] Leventis, N.; Rawaswdeh, A.-M. M.; Zhang, G.; Elder, I. A.; Sotiriou-Leventis, C. *J. Org. Chem.* **2002**, *67*, 7501-7510.

[9] Ren, L.; Wang, L.; Lv, Y.; Li, G.; Gao, S. *Org. Lett.* **2015**, *17*, 2078-2081.

[10] Toh, Q. Y.; McNally, A.; Vera, S.; Erdmann, N.; Gaunt, M. J. *J. Am. Chem. Soc.* **2013**, *135*, 3772-3775.

[11] Kuriyama, M.; Hamaguchi, N.; Sakata, K.; Onomura, O. *Eur. J. Org. Chem.* **2013**, *2013*, 3378-3385.

[12] Solange, A. *Tetrahedron* **1989**, *45*, 1409-1414.

[13] Keenan, M.; Abbott, M. J.; Alexander, P. W.; Armstrong, T.; Best, W. M.; Berven, B.; Botero, A.; Chaplin, J. H.; Charman, S. A.; Chatelain, E.; von Geldern, T. W.; Kerfoot, M.; Khong, A.; Nguyen, T.; McManus, J. D.; Morizzi, J.; Ryan, E.; Scandale, I.; Thompson, R. A.; Wang, S. Z.; White, K. L. *J. Med. Chem.* **2012**, *55*, 4189-4204.

[14] Someya, C. I.; Inoue, S.; Krackl, S.; Irran, E.; Enthaler, S. *Eur. J. Inorg. Chem.* **2012**, *2012*, 1269-1277.

[15] Efange, S. M. N.; Michelson, R. H.; Remmel, R. P.; Boudreau, R. J.; Dutta, A. K.; Freshler, A. *J. Med. Chem.* **1990**, *33*, 3133-3138.

[16] Ágai, B.; Proszenyák, Á.; Tárkányi, G.; Vida, L.; Faigl, F. *Eur. J. Org. Chem.* **2004**, *2004*, 3623-3632.

[17] Hu, Q.; Jagusch, C.; Hille, U. E.; Haupenthal, J.; Hartmann, R. W. *J. Med. Chem.* **2010**, *53*, 5749-5758.

[18] Shang, R.; Yang, Z.-W.; Wang, Y.; Zhang, S.-L.; Liu, L. *J. Am. Chem. Soc.* **2010**, *132*, 14391-14393.

[19] De Houwer, J.; Abbaspour Tehrani, K.; Maes, B. U. W. *Angew. Chem. Int. Ed.* **2012**, *51*, 2745-2748.

[20] Augustine, J. K.; Naik, Y. A.; Mandal, A. B.; Kundapur, U. *J. Org. Chem.* **2008**, *73*, 1176-1179.

[21] Hermann, T. W.; Piotrowska K.; Jarzębkiewicz J.; *Acta Pol. Pharm.* **2005**, *4*, 295-297.

[22] Frisch, M. J.; Trucks, G. W.; Schlegel, H. B.; Scuseria, G. E.; Robb, M. A.; Cheeseman, J. R.; Scalmani, G.; Barone, V.; Mennucci, B.; Petersson, G. A.; Nakatsuji, H.; Caricato, M.; Li, X.; Hratchian, H. P.; Izmaylov, A. F.; Bloino, J.; Zheng, G.; Sonnenberg, J. L.; Hada, M.; Ehara, M.; Toyota, K.; Fukuda, R.; Hasegawa, J.; Ishida, M.; Nakajima, T.; Honda, Y.; Kitao, O.; Nakai, H.; Vreven, T.; Montgomery Jr., J. A.; Peralta, J. E.; Ogliaro, F.; Bearpark, M. J.; Heyd, J.; Brothers, E. N.; Kudin, K. N.; Staroverov, V. N.; Kobayashi, R.; Normand, J.; Raghavachari, K.; Rendell, A. P.; Burant, J. C.; Iyengar, S. S.; Tomasi, J.; Cossi, M.; Rega, N.; Millam, N. J.; Klene, M.; Knox, J. E.; Cross, J. B.; Bakken, V.; Adamo, C.; Jaramillo, J.; Gomperts, R.; Stratmann, R. E.; Yazyev, O.; Austin, A. J.; Cammi, R.; Pomelli, C.; Ochterski, J. W.; Martin, R. L.; Morokuma, K.; Zakrzewski, V. G.; Voth, G. A.; Salvador, P.; Dannenberg, J. J.; Dapprich, S.; Daniels, A. D.; Farkas, Ö.; Foresman, J. B.; Ortiz, J. V.; Cioslowski, J.; Fox, D. J.; Gaussian, Inc.: Wallingford, CT, USA, 2009.

[23] Sterckx, H.; De Houwer, J.; Mensch, C.; Caretti, I.; Tehrani, K. A.; Herrebout, W. A.; Van Doorslaer, S.; Maes, B. U. W. *Chem. Sci.* **2015**, DOI: 10.1039/C5SC03530A

# VII. NMR Spectra

6-Benzoylnicotinonitrile

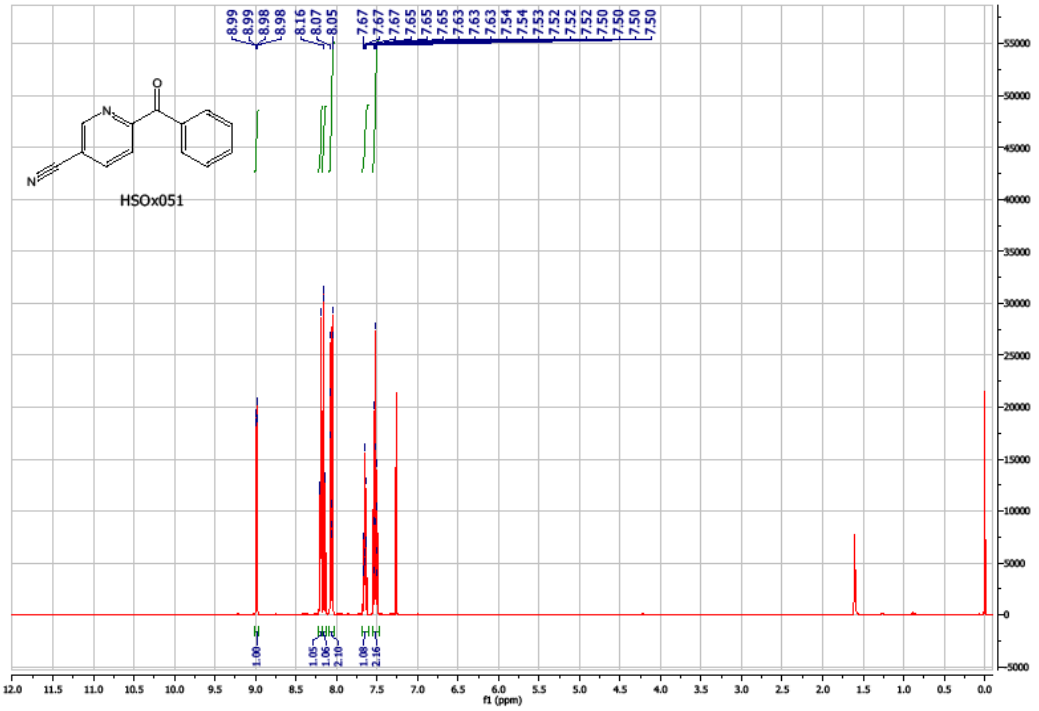


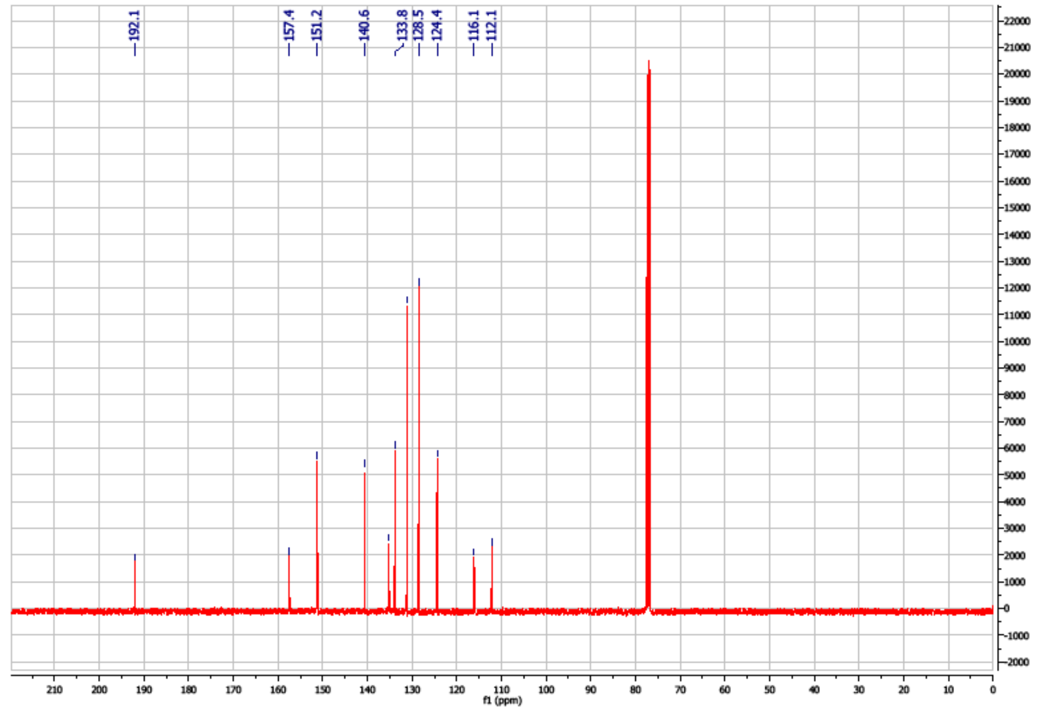

(5-Methylpyridin-2-yl)(phenyl)methanone

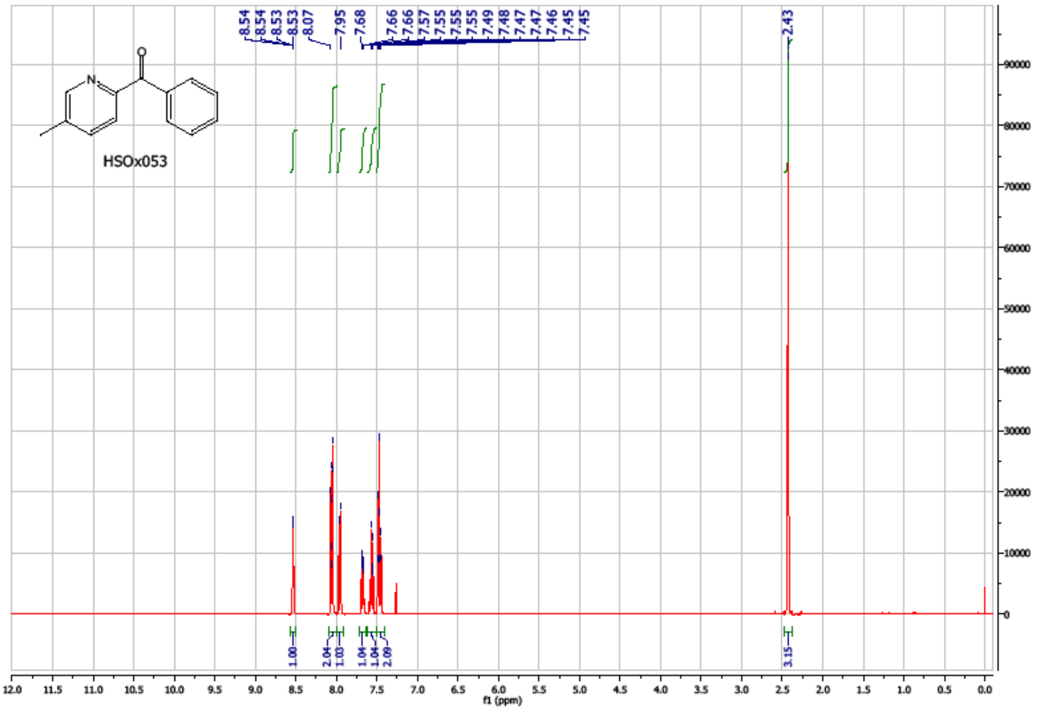


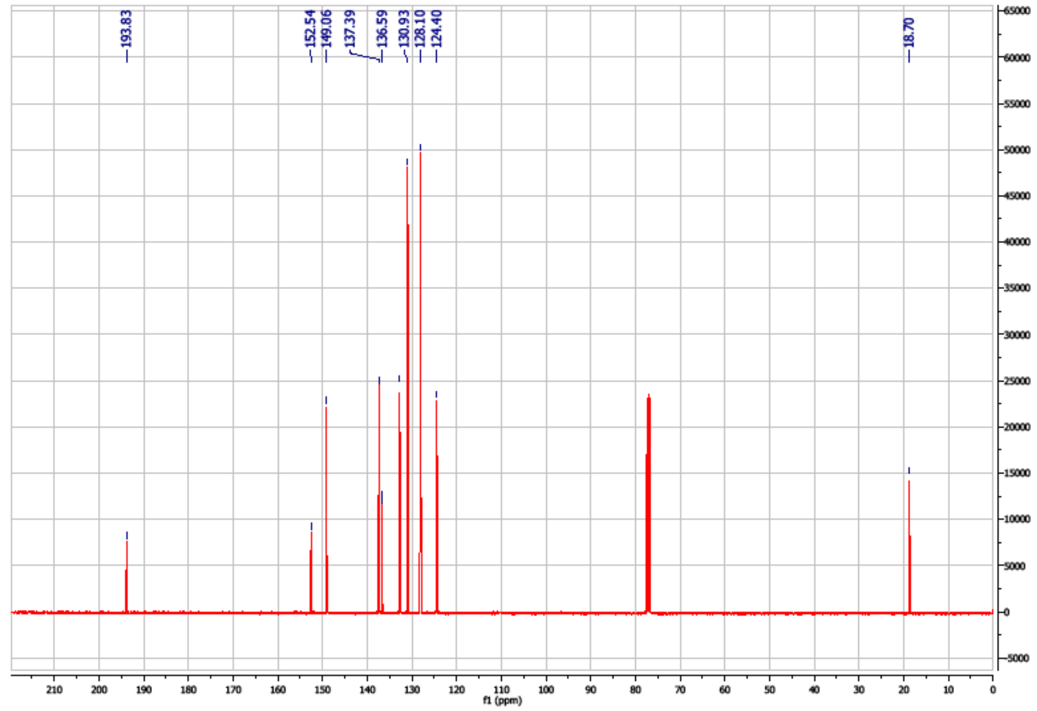

(5-Methoxypyridin-2-yl)(phenyl)methanone

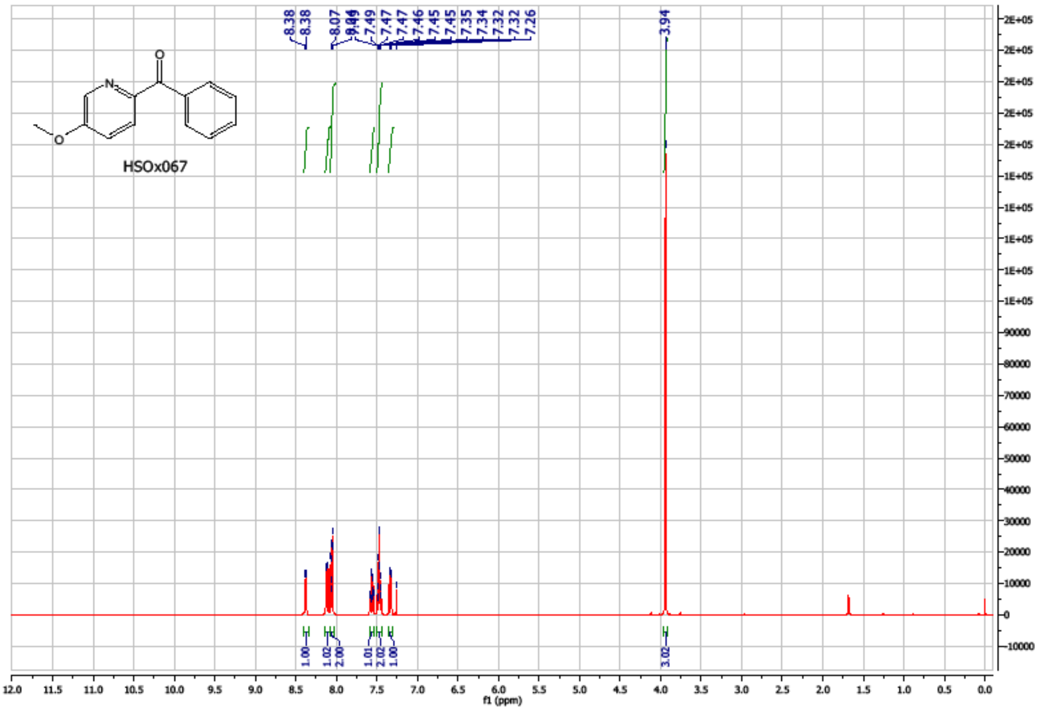


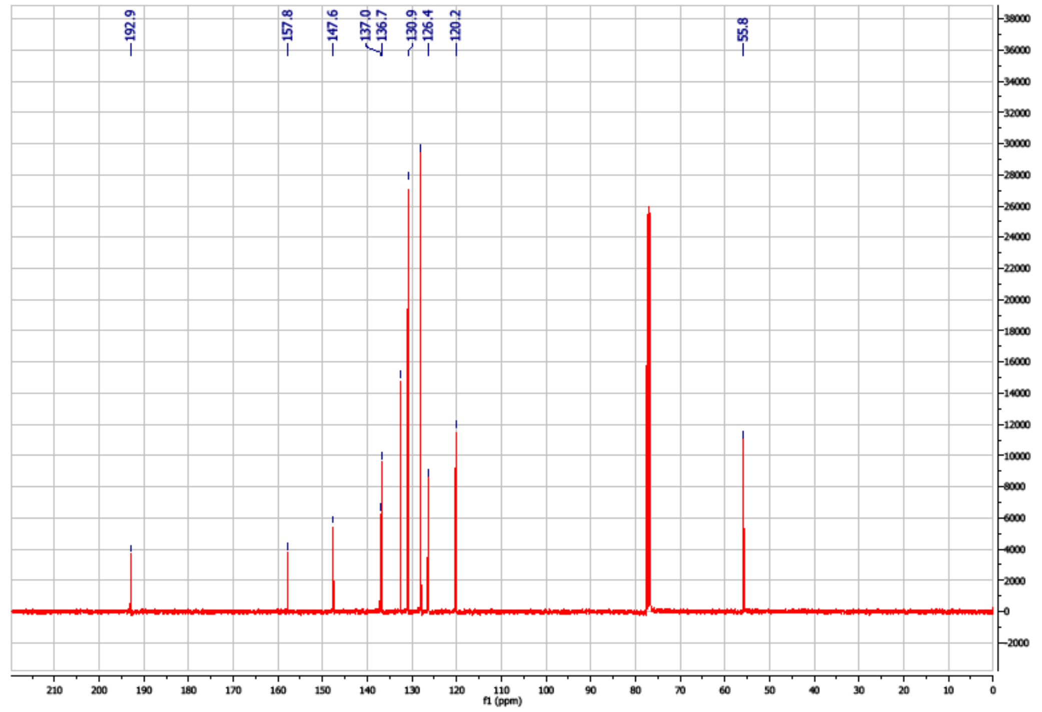

Methyl 6-benzoylnicotinate

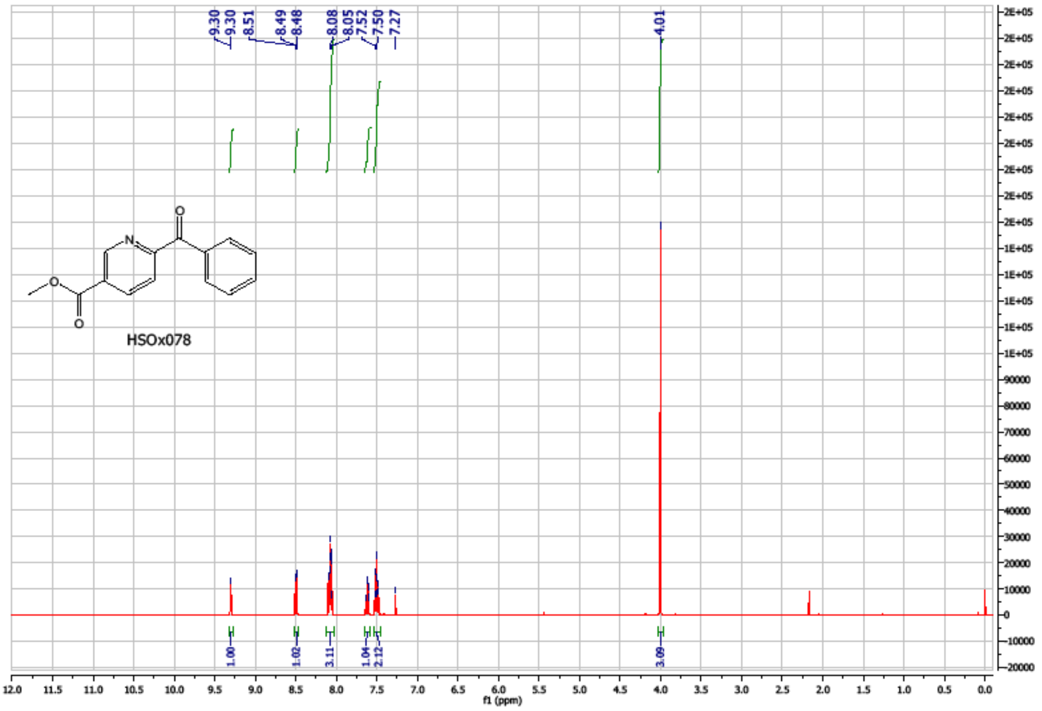


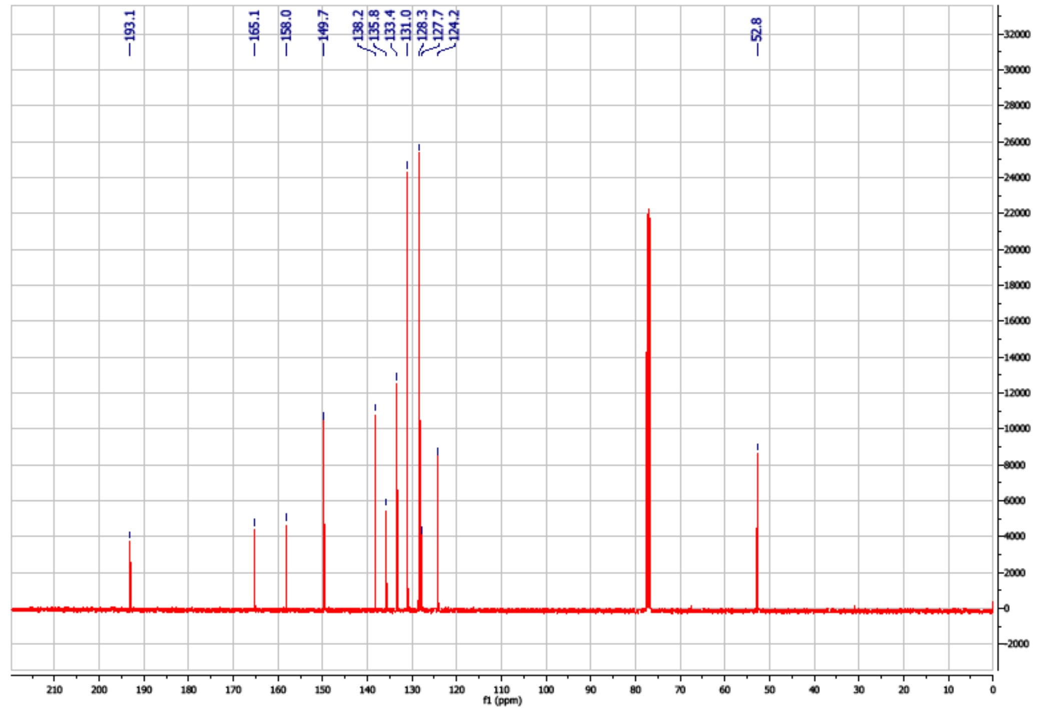

*N*-(6-Benzoylpyridin-3-yl)acetamide

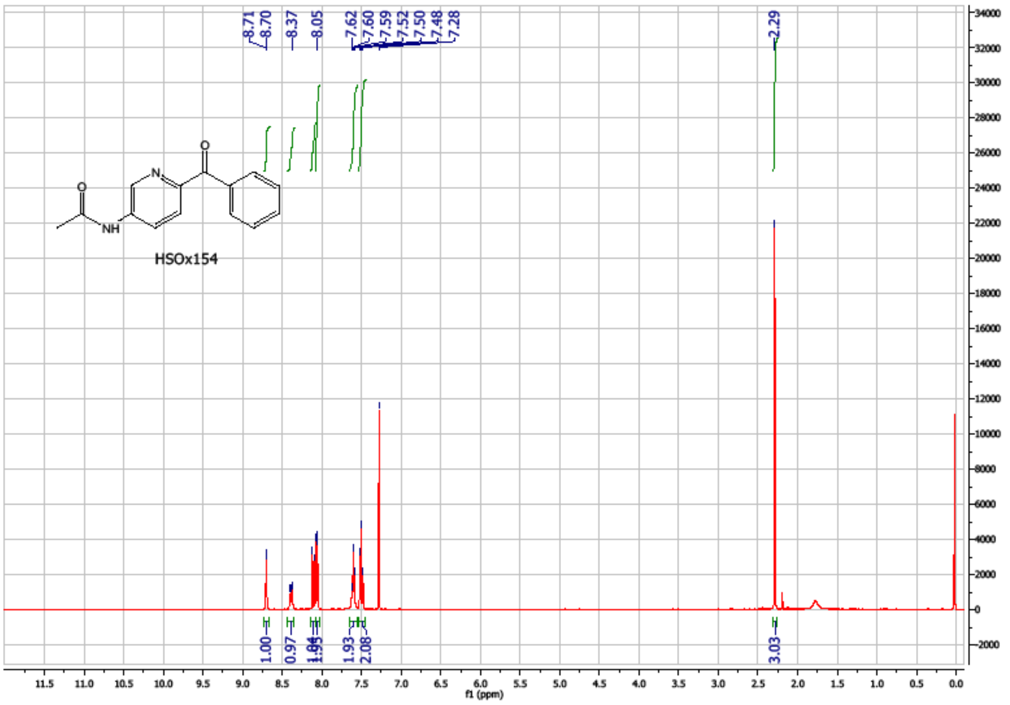


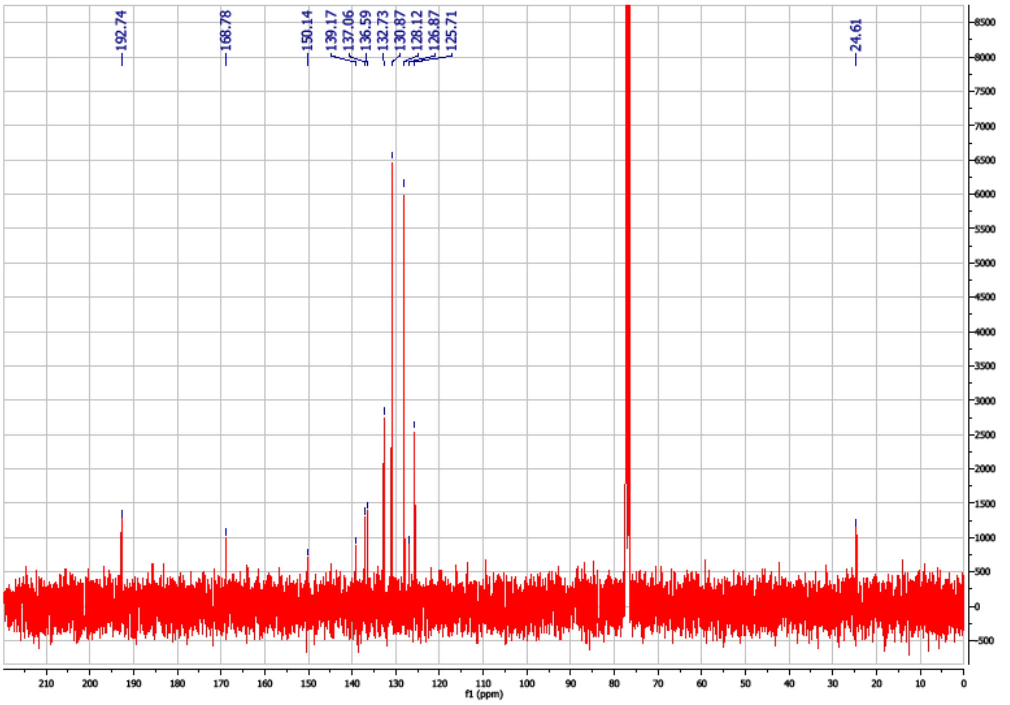

(4-Chloropyridin-2-yl)(phenyl)methanone

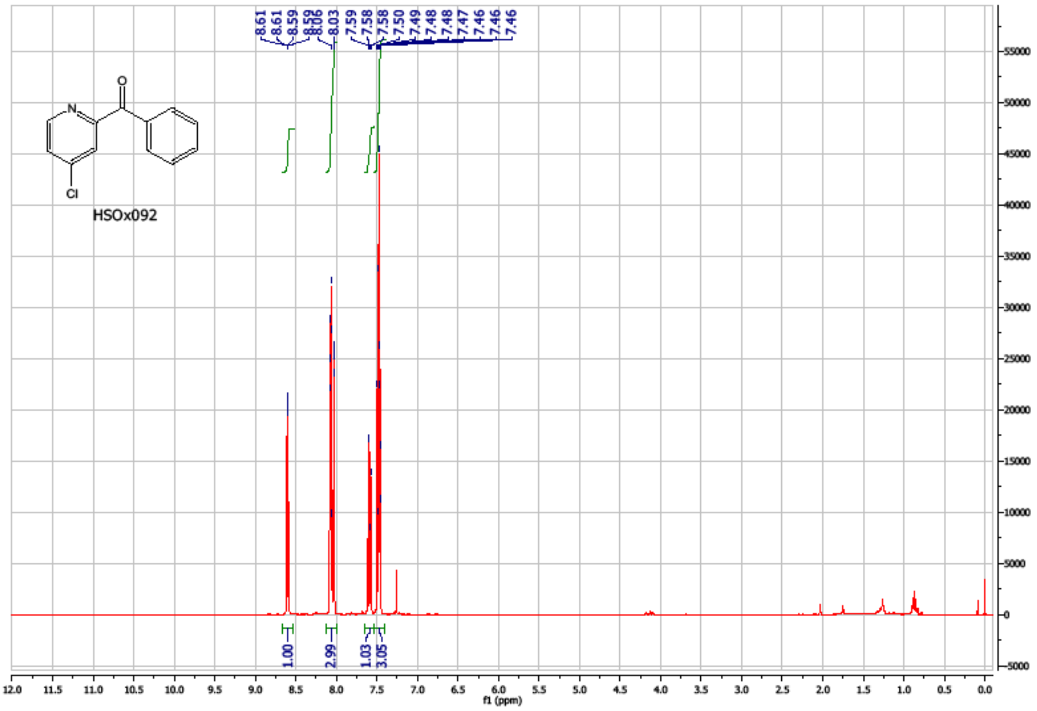


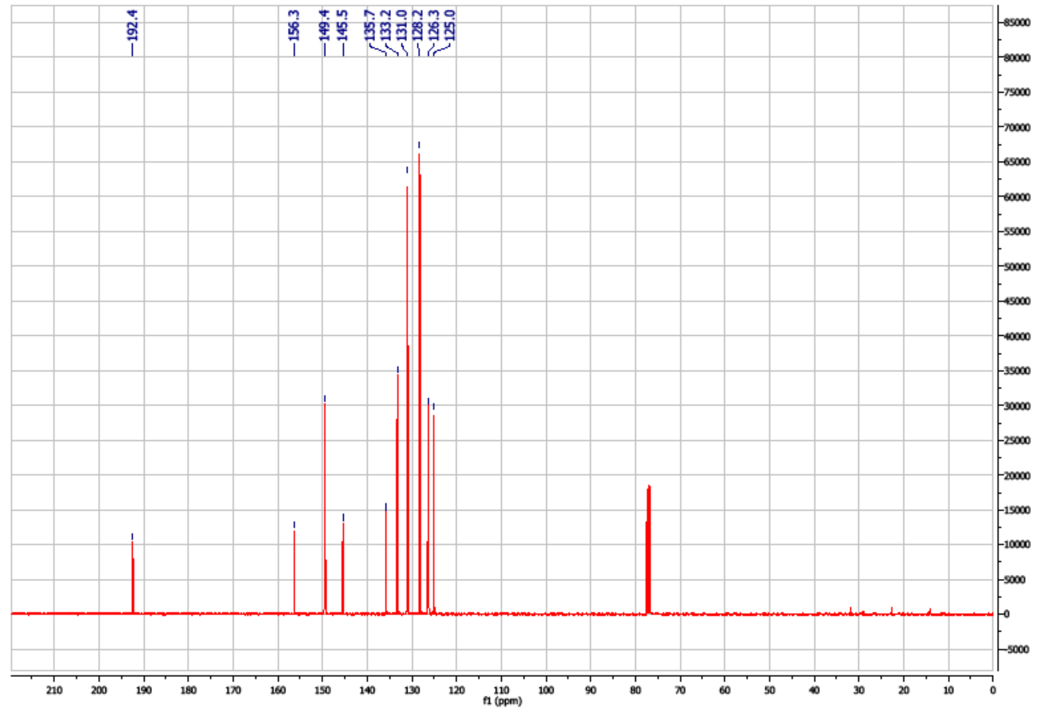

(3-Chloropyridin-2-yl)(phenyl)methanone

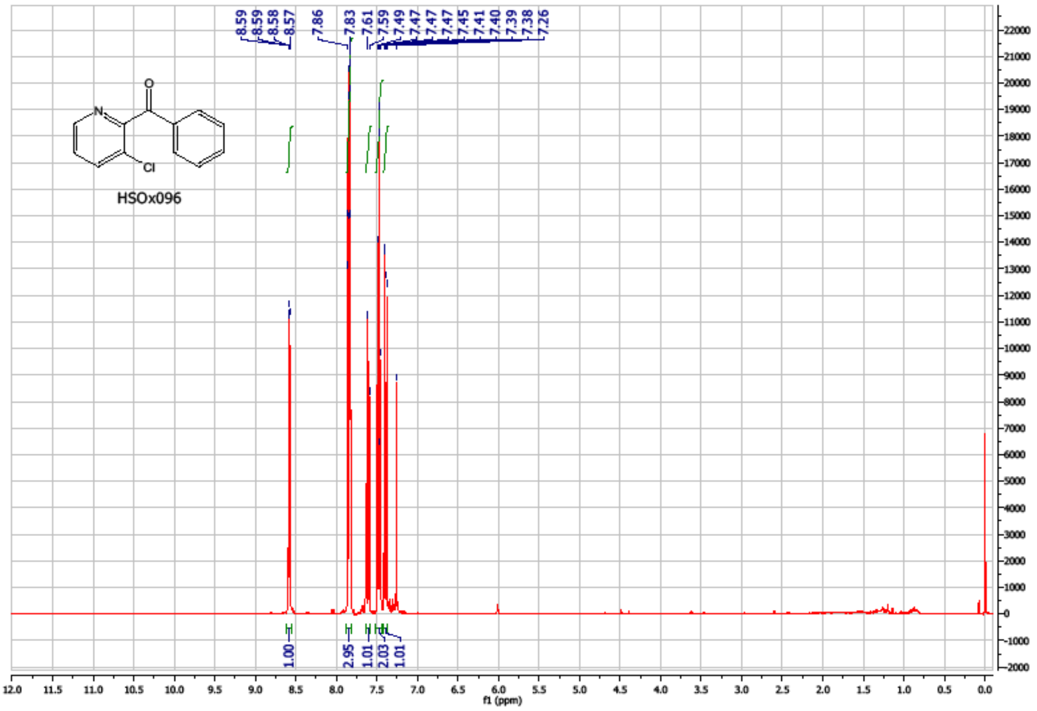


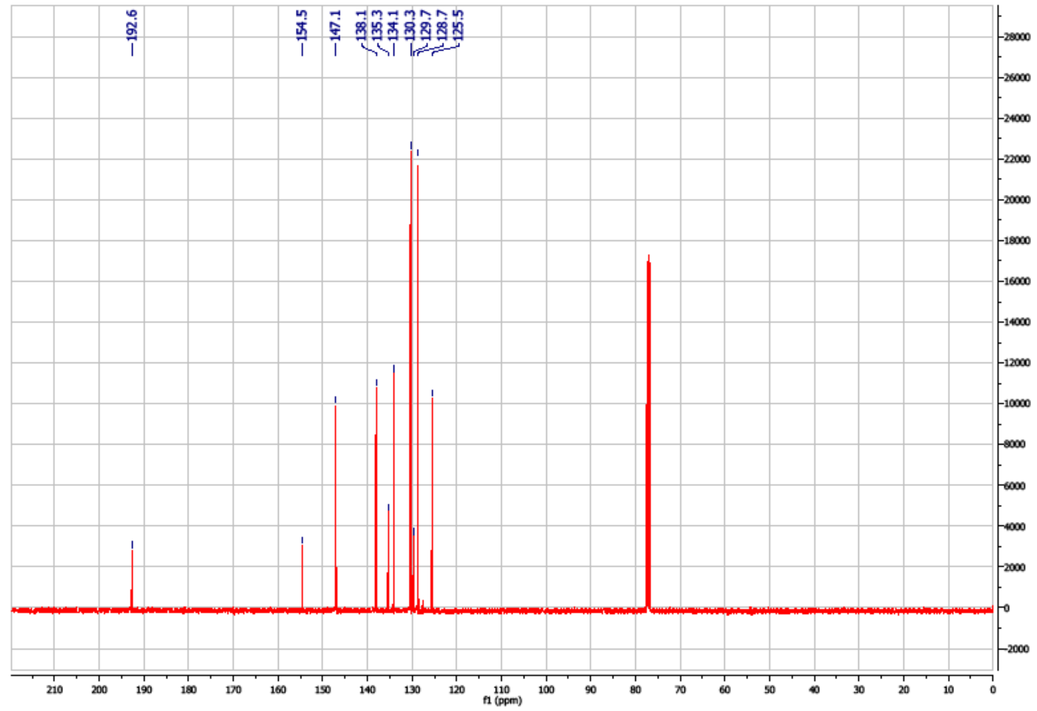

(5-Chloropyridin-2-yl)(phenyl)methanone

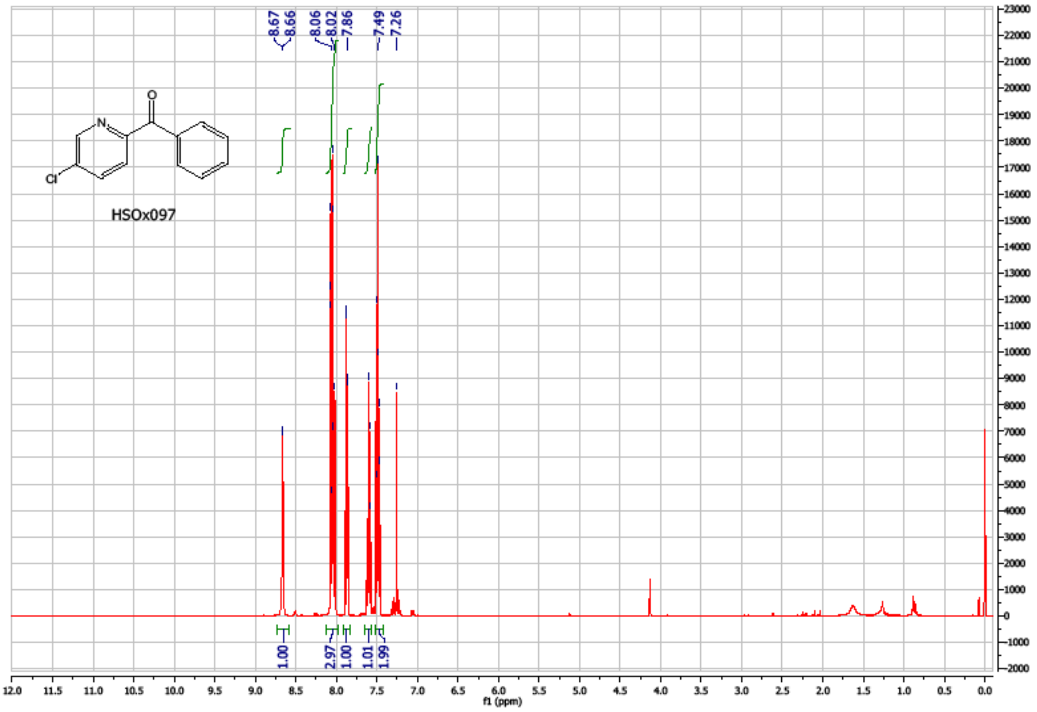


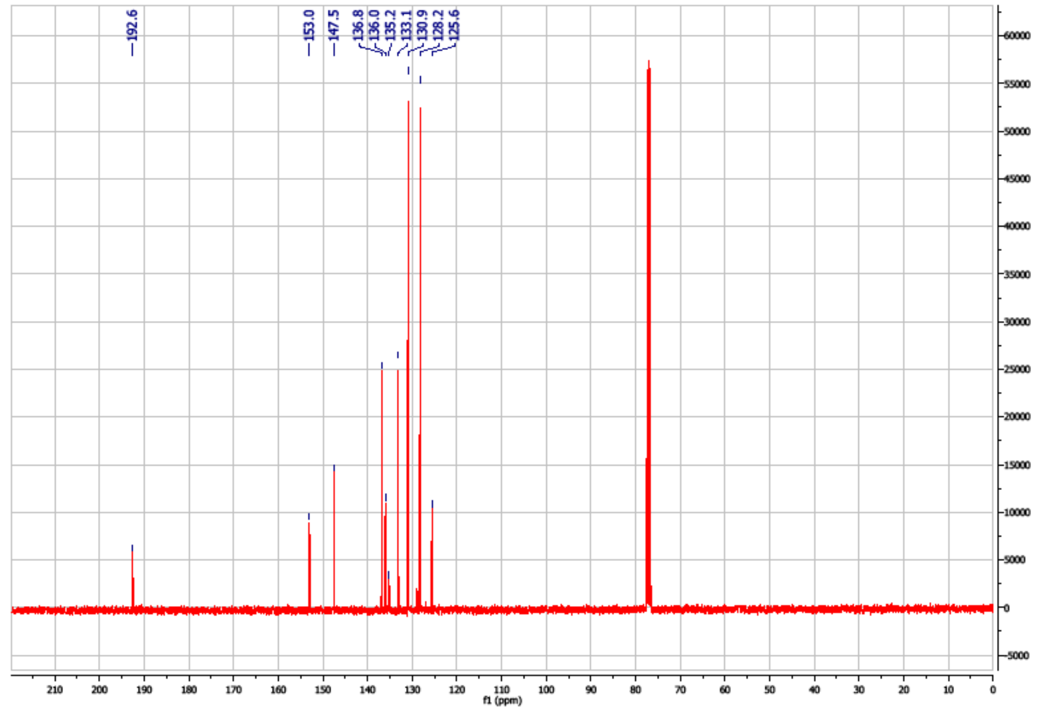

(6-Chloropyridin-2-yl)(phenyl)methanone

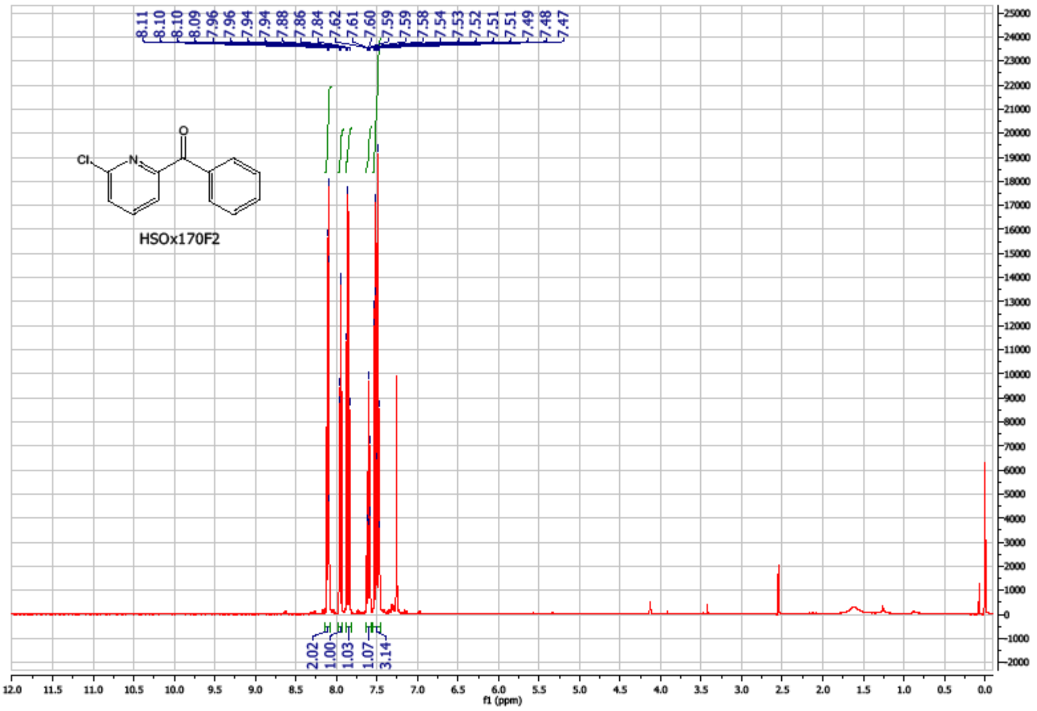


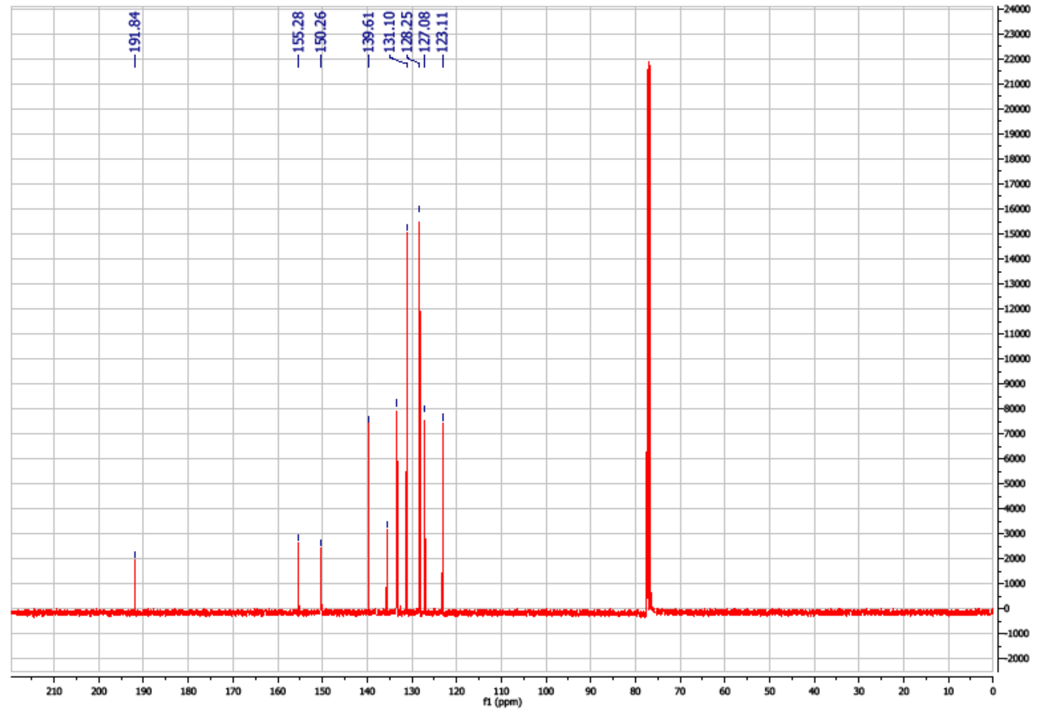

(6,7-Dimethoxyisoquinolin-1-yl)(3,4-dimethoxyphenyl)methanone

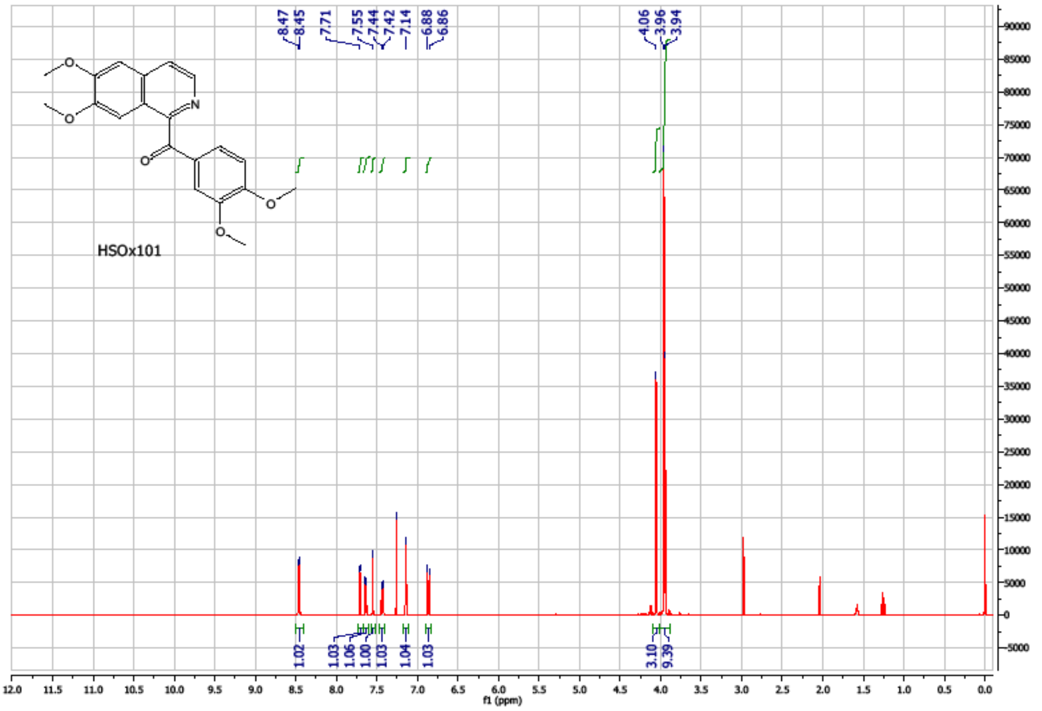


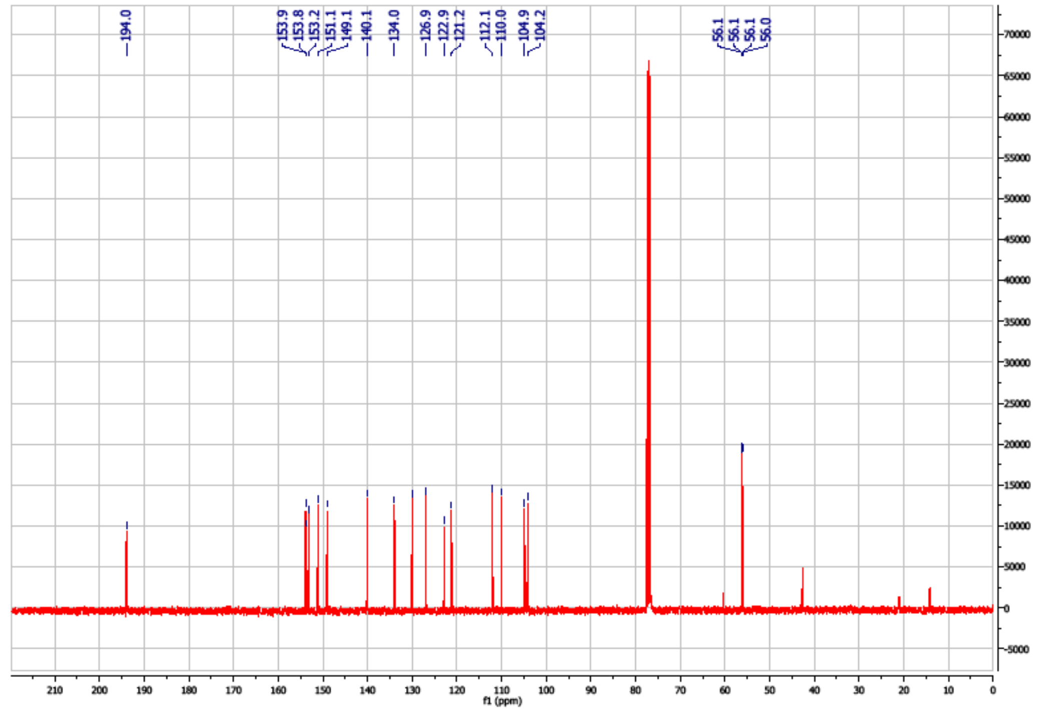

(2-Methylpyrimidin-4-yl)(phenyl)methanone

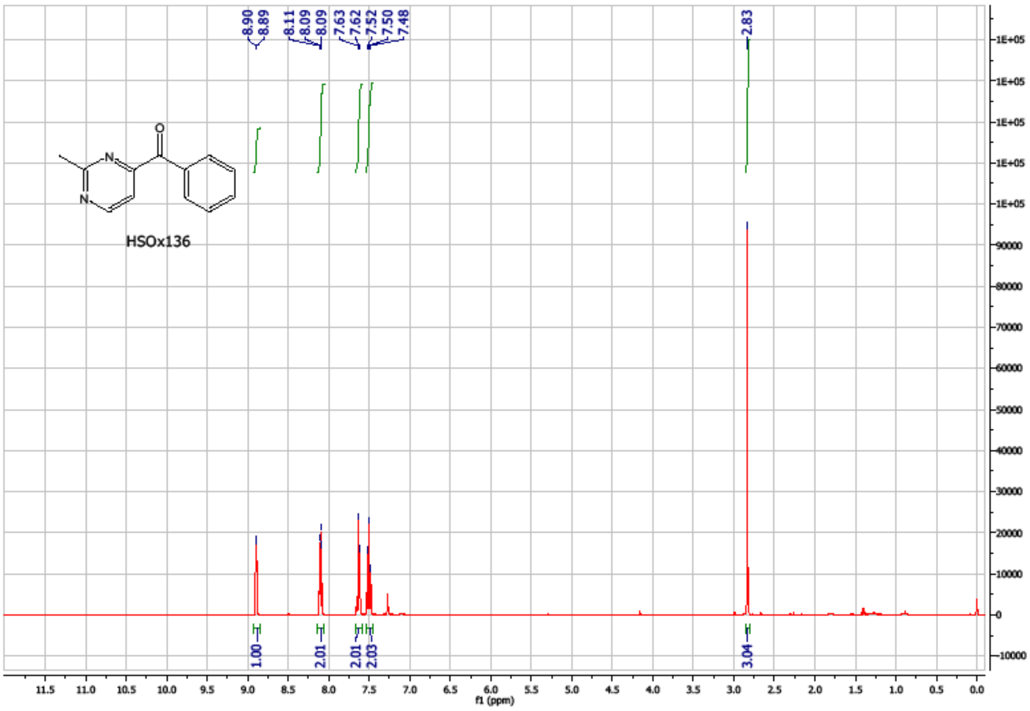


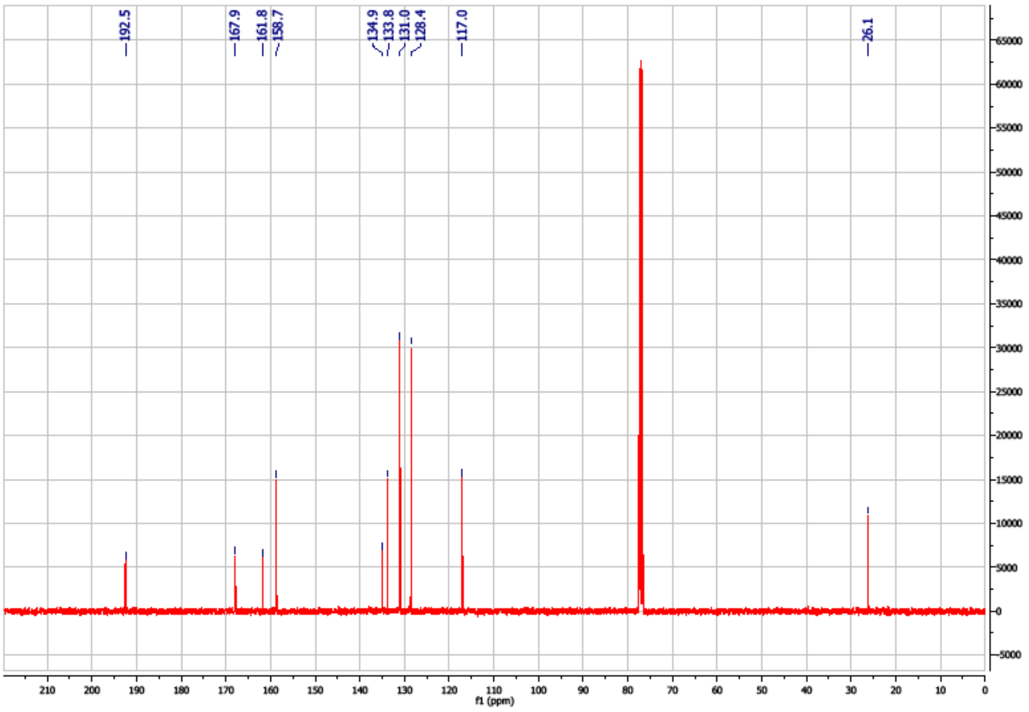

(6-Methylpyridazin-3-yl)(phenyl)methanone

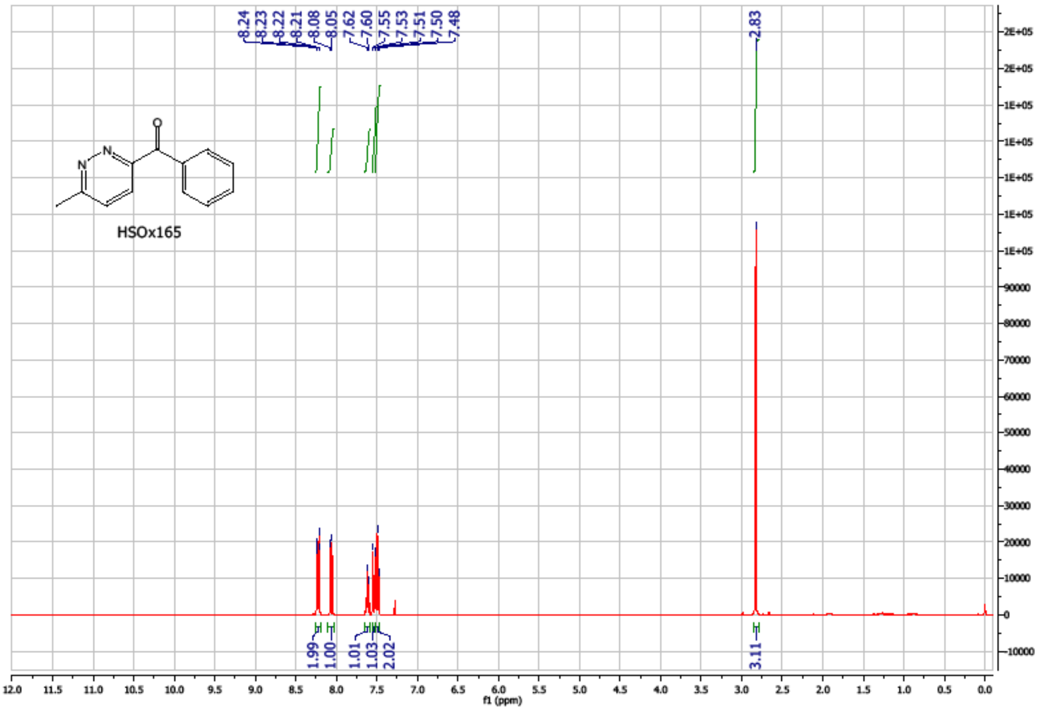


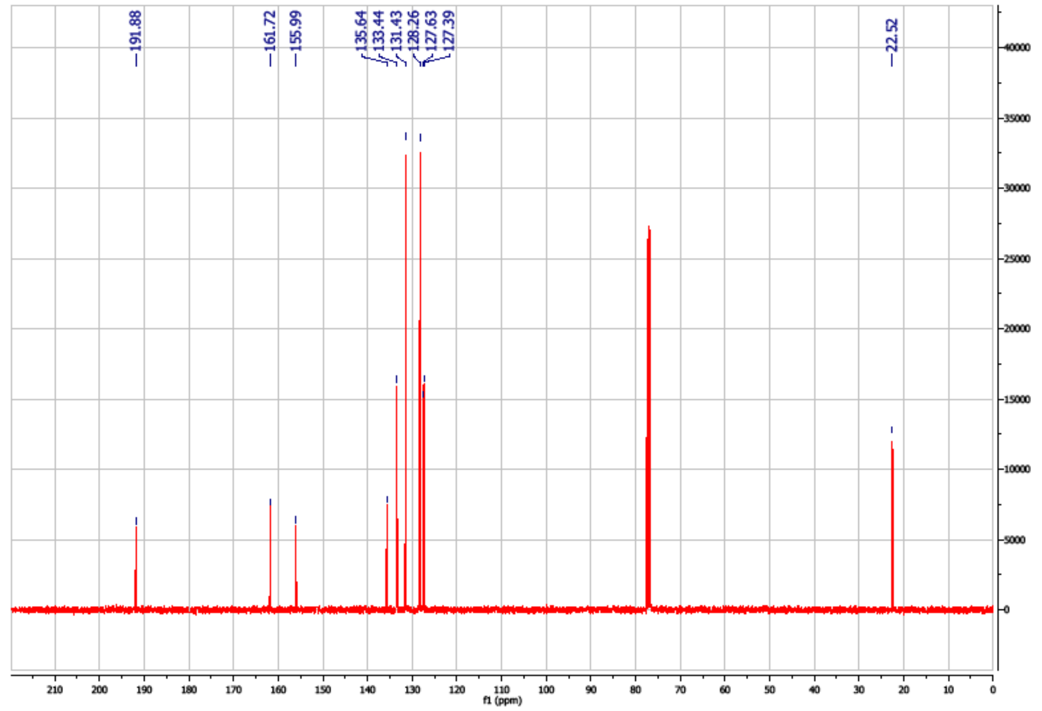

[4-(Methylthio)phenyl](pyridin-4-yl)methanone

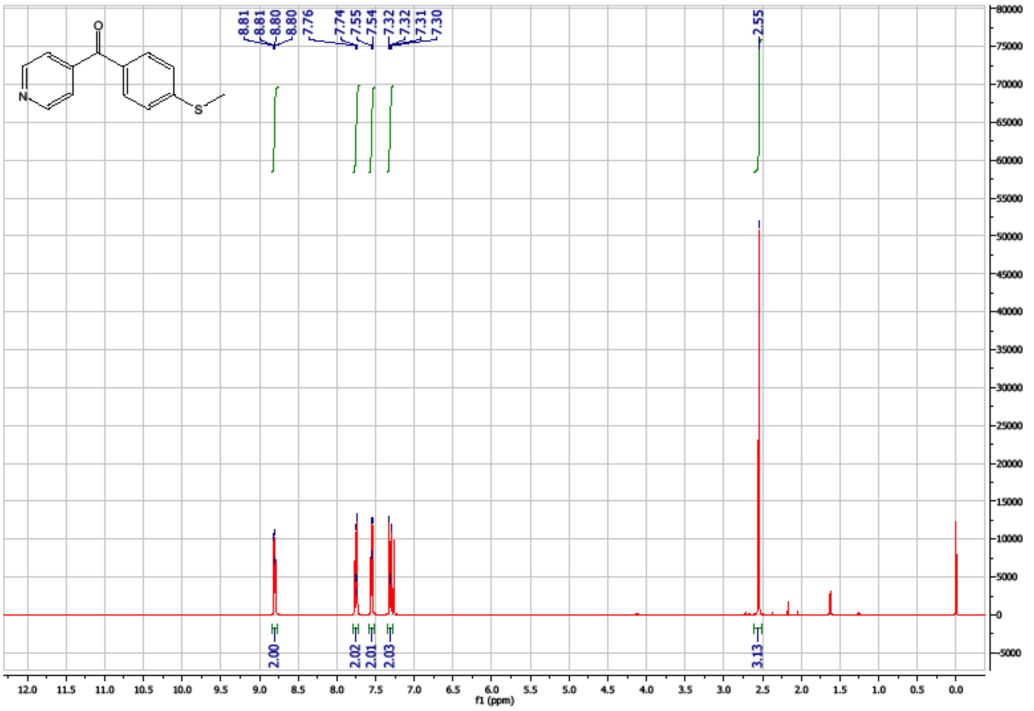


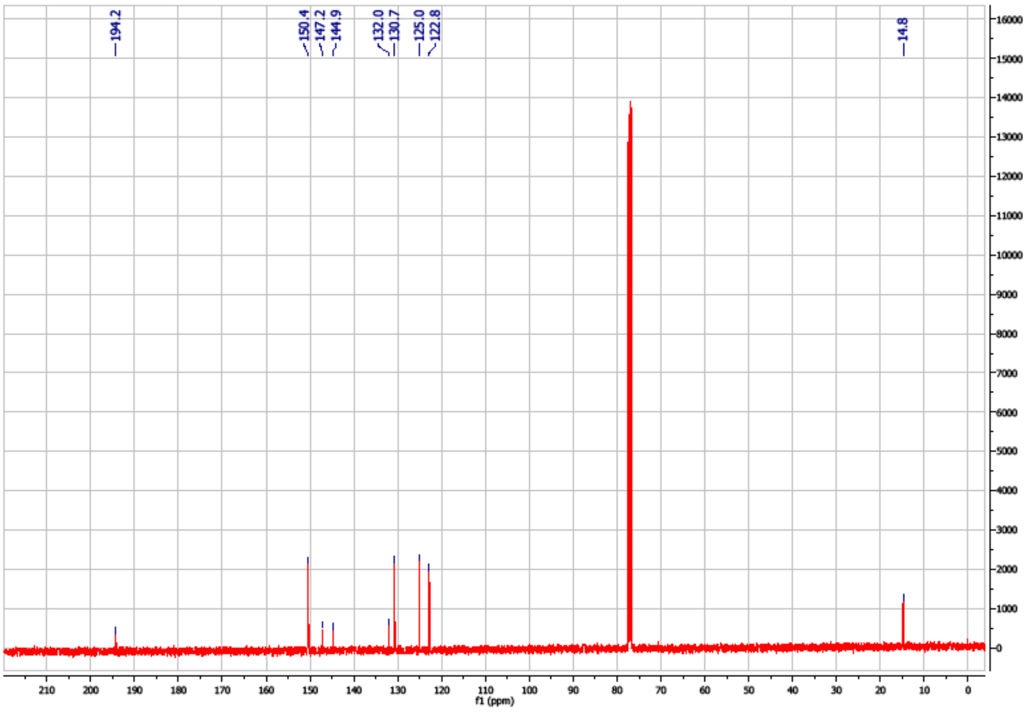

(4-Methoxyphenyl)(pyridin-4-yl)methanone

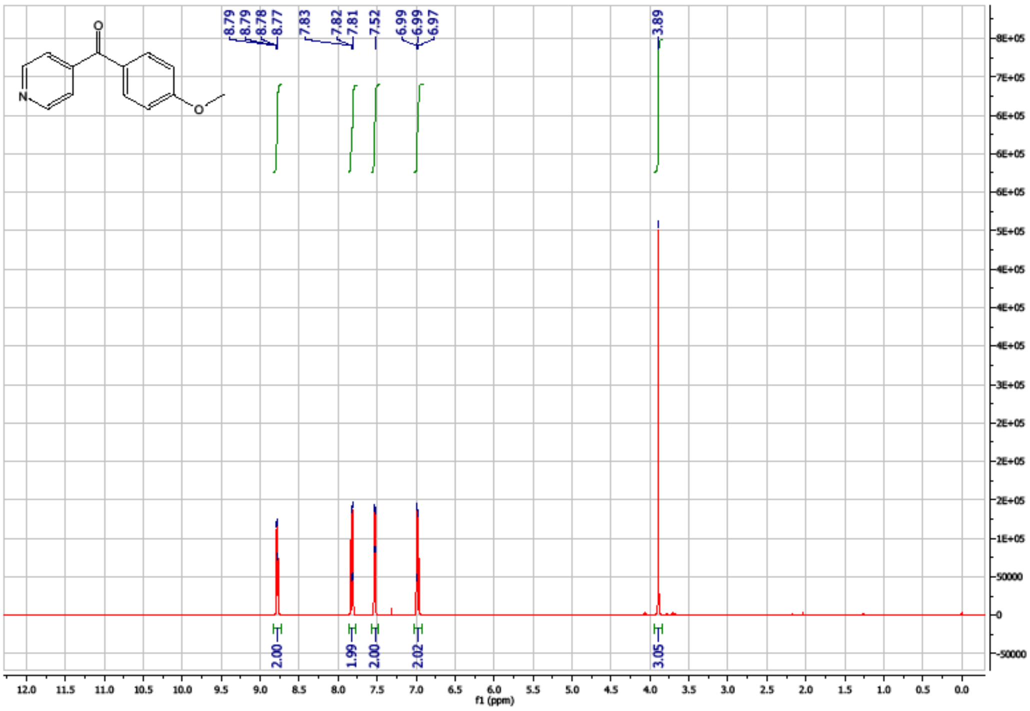


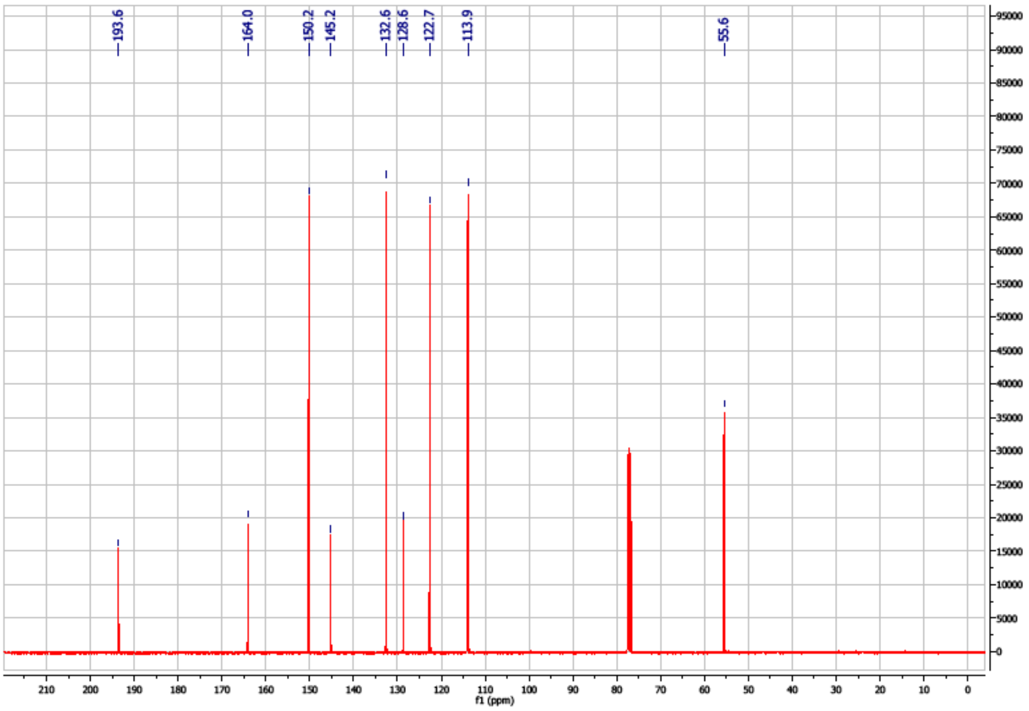

(4-Methylphenyl)(pyridin-4-yl)methanone

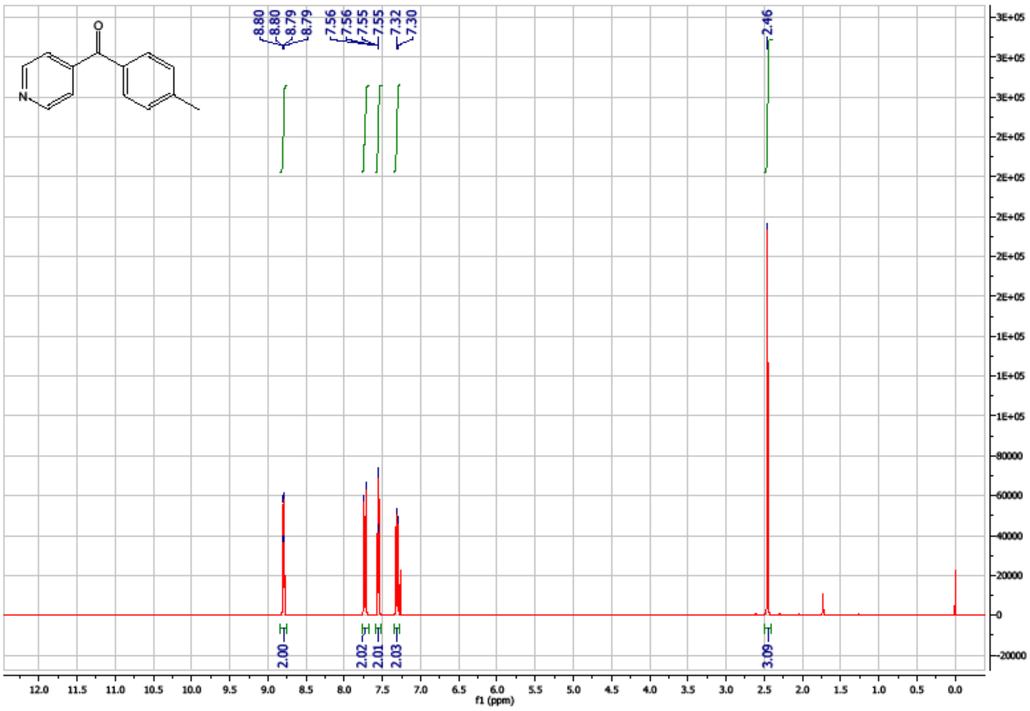

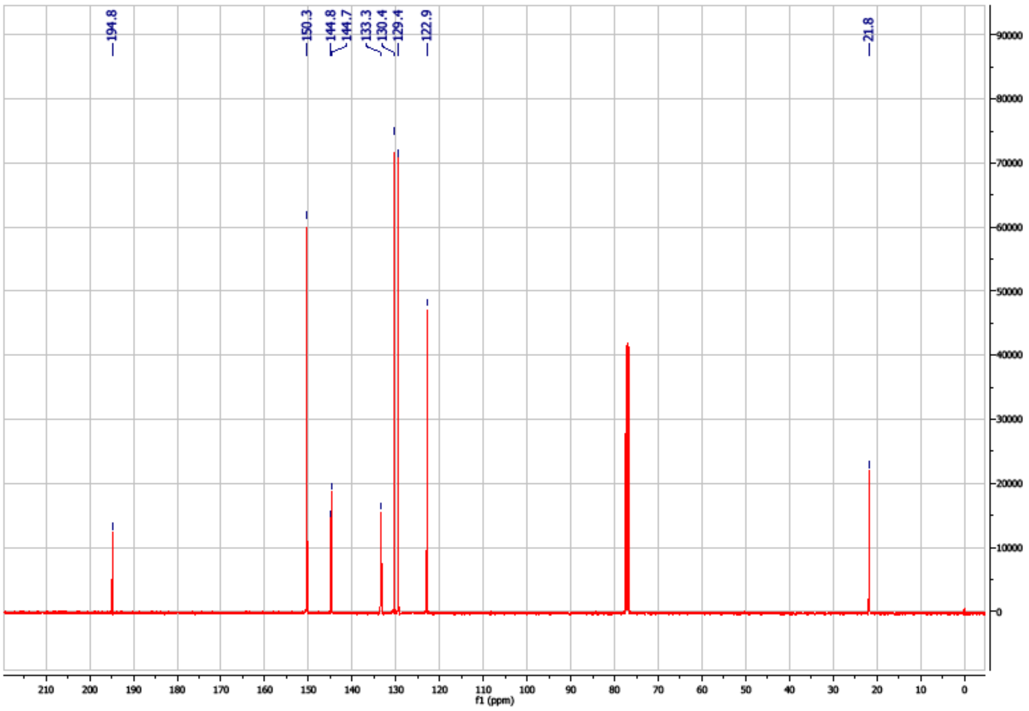

(4-Chlorophenyl)(pyridin-4-yl)methanone

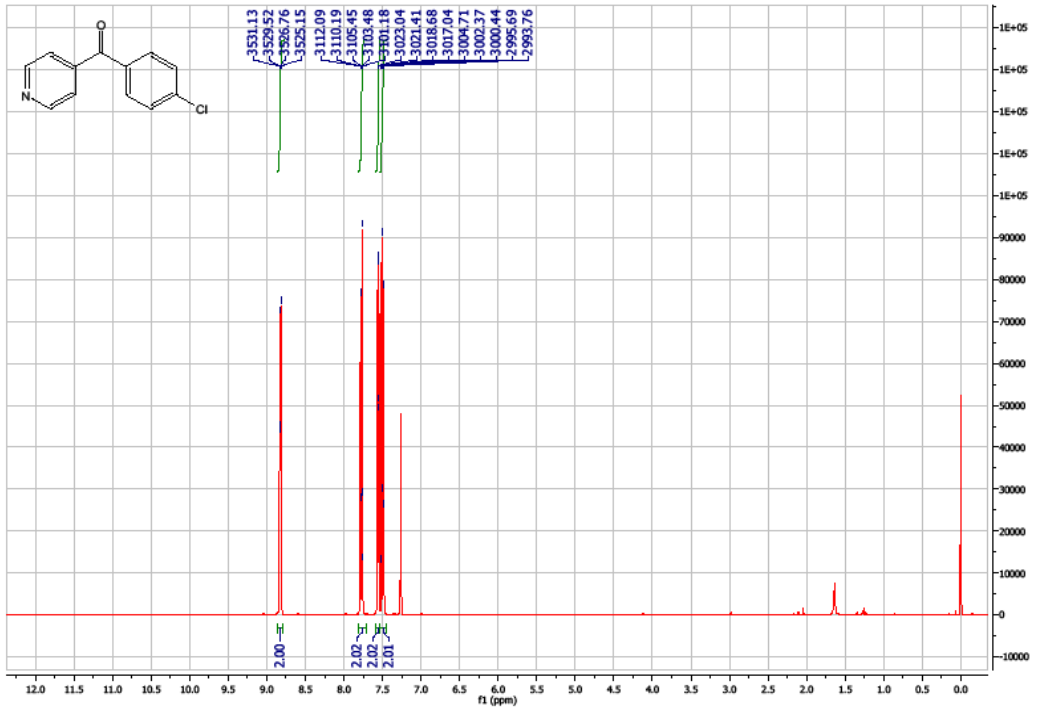

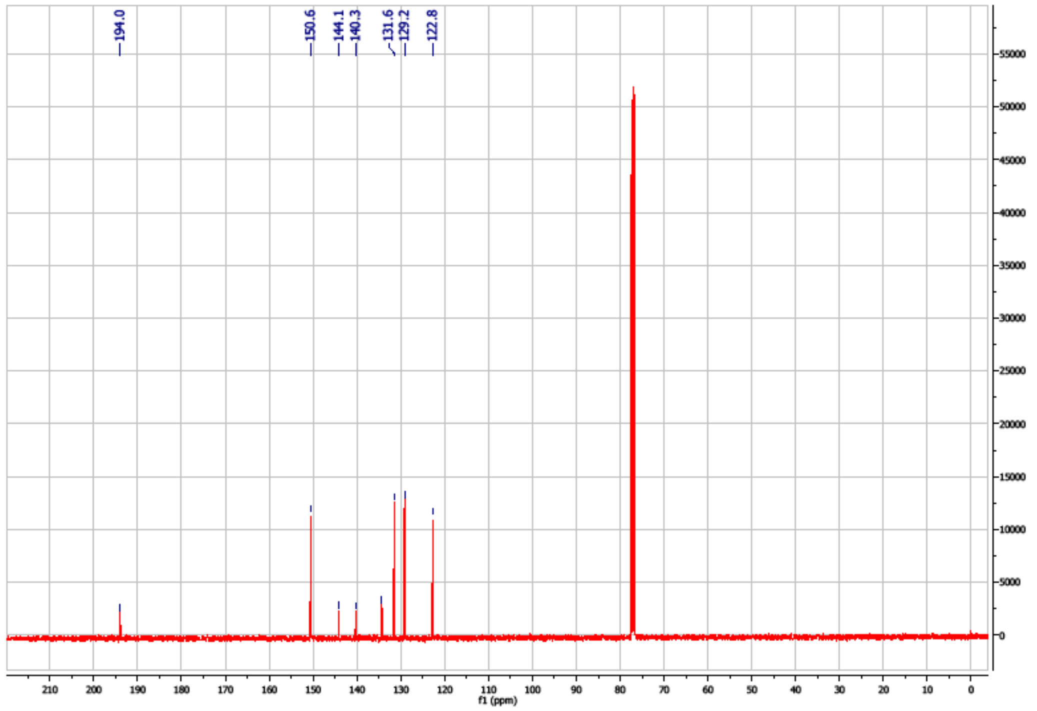

(4-Fluorophenyl)(pyridin-4-yl)methanone

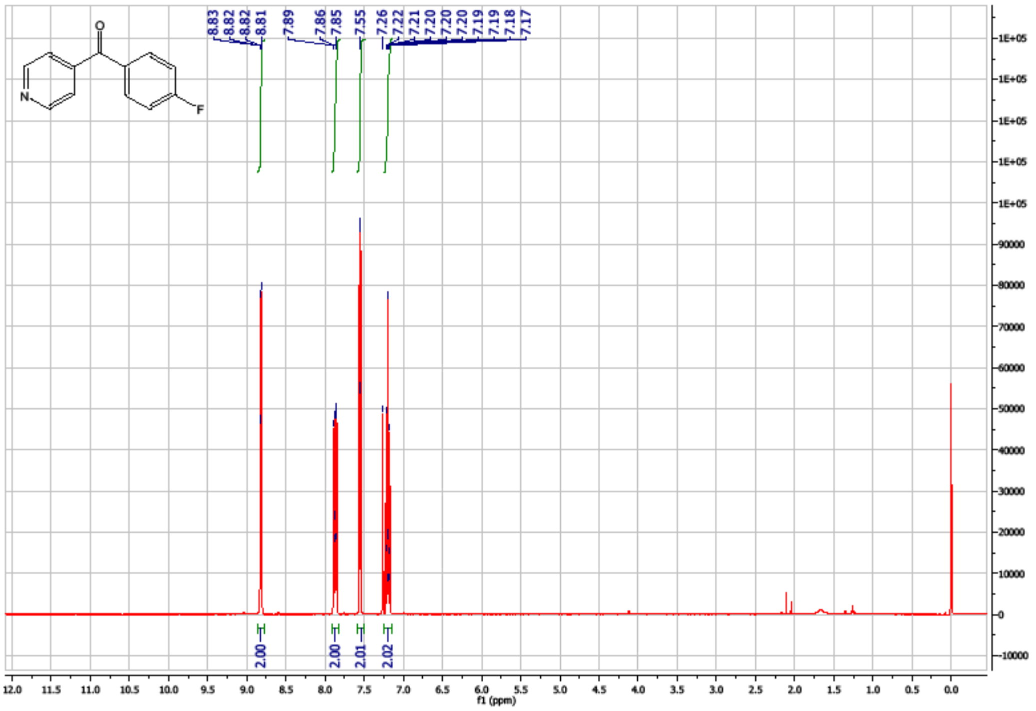


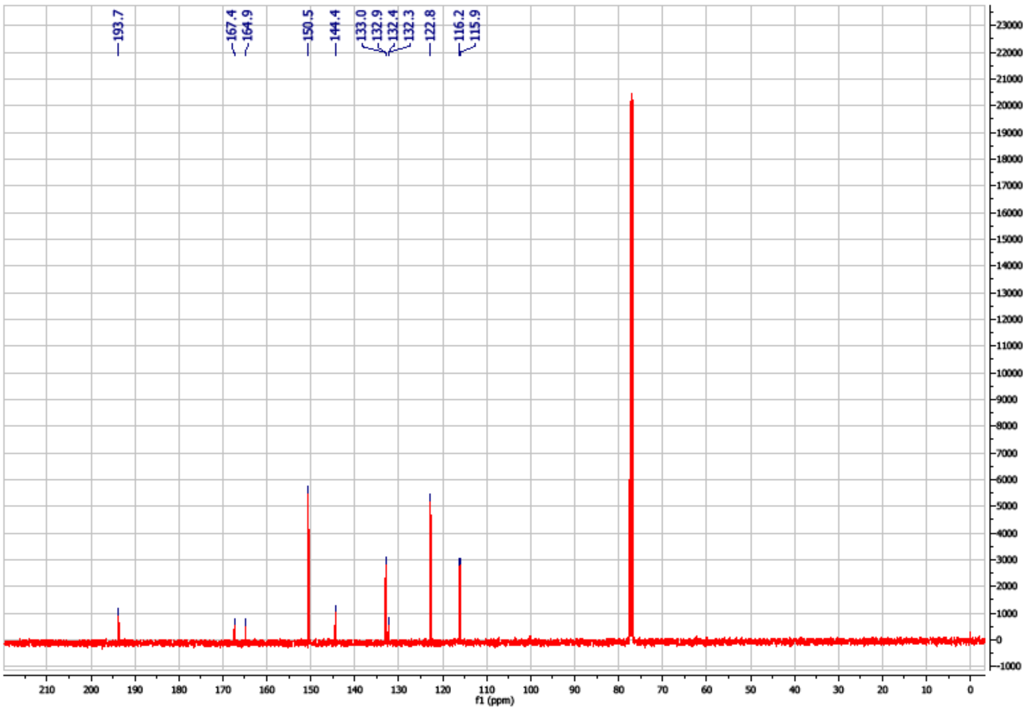

Ethyl 4-(pyridin-4-ylcarbonyl)benzoate

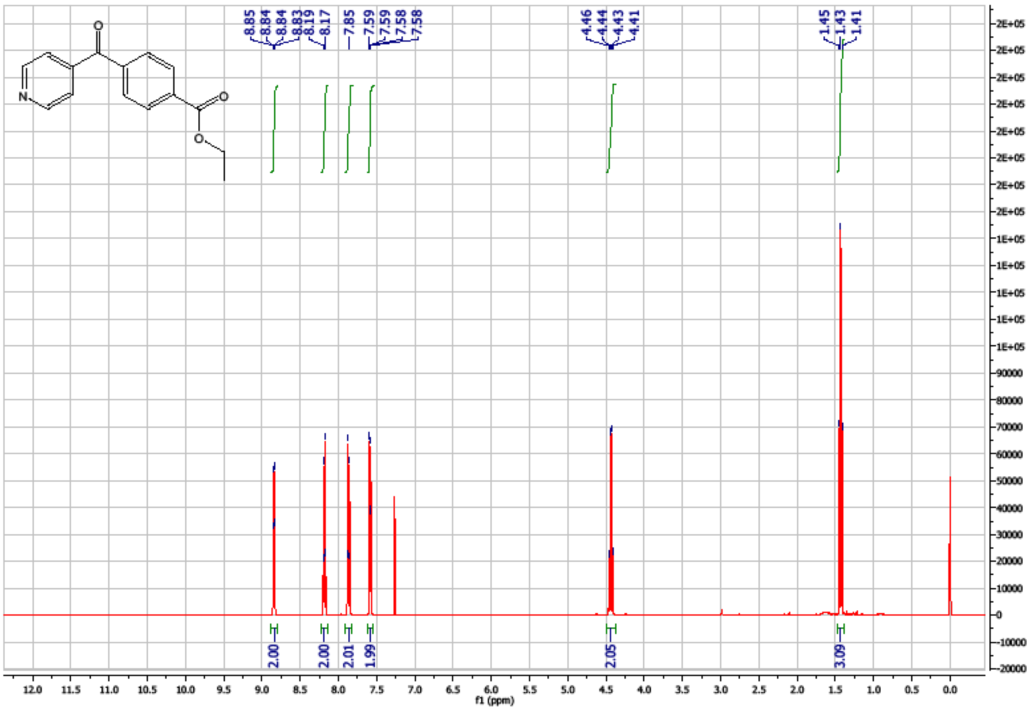

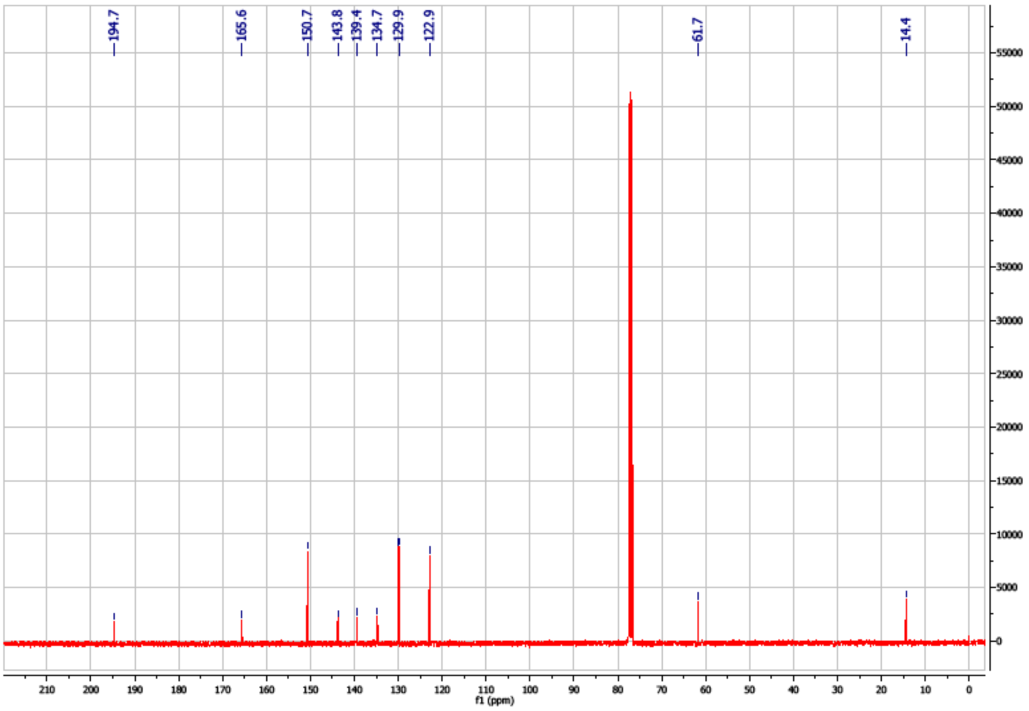

4-(Pyridin-4-ylcarbonyl)benzonitrile

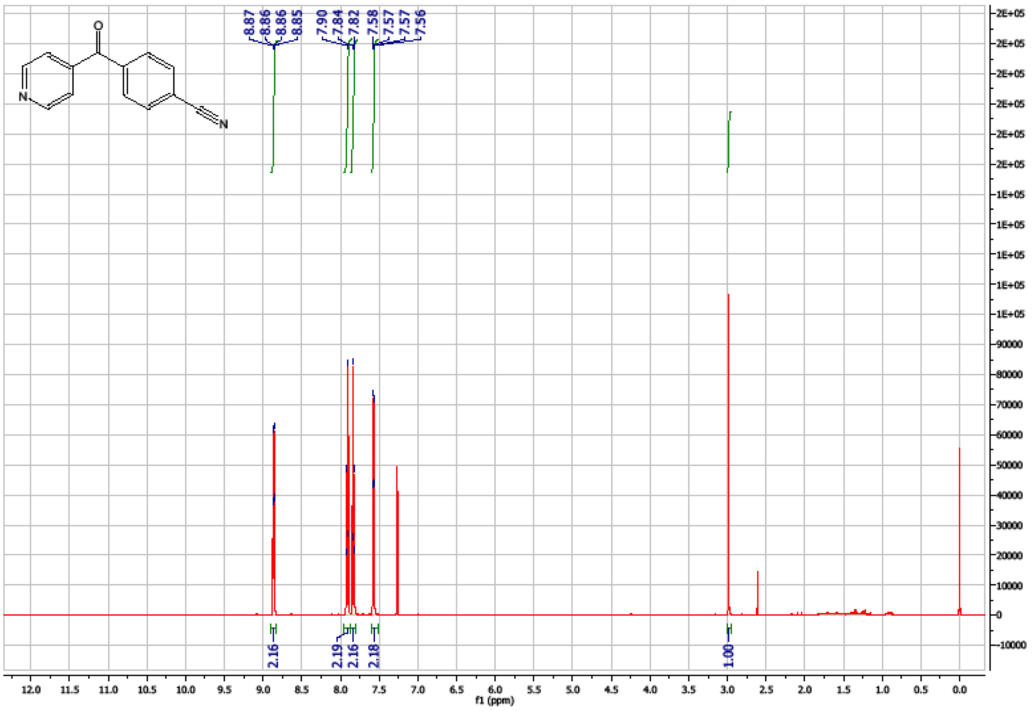


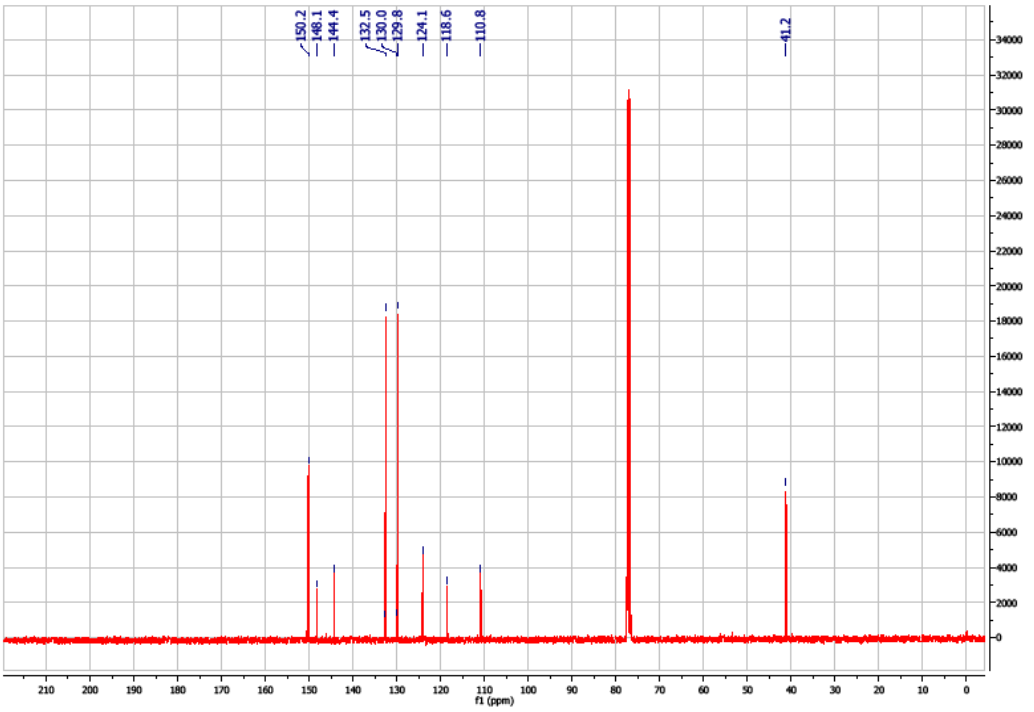

(4-Nitrophenyl)(pyridin-4-yl)methanone

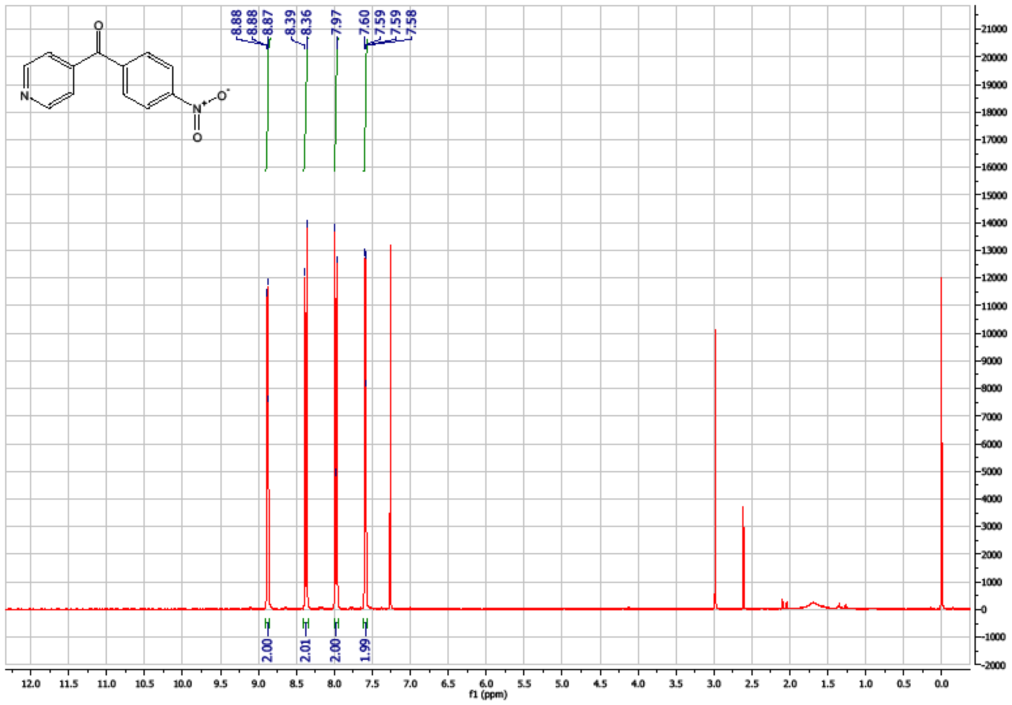

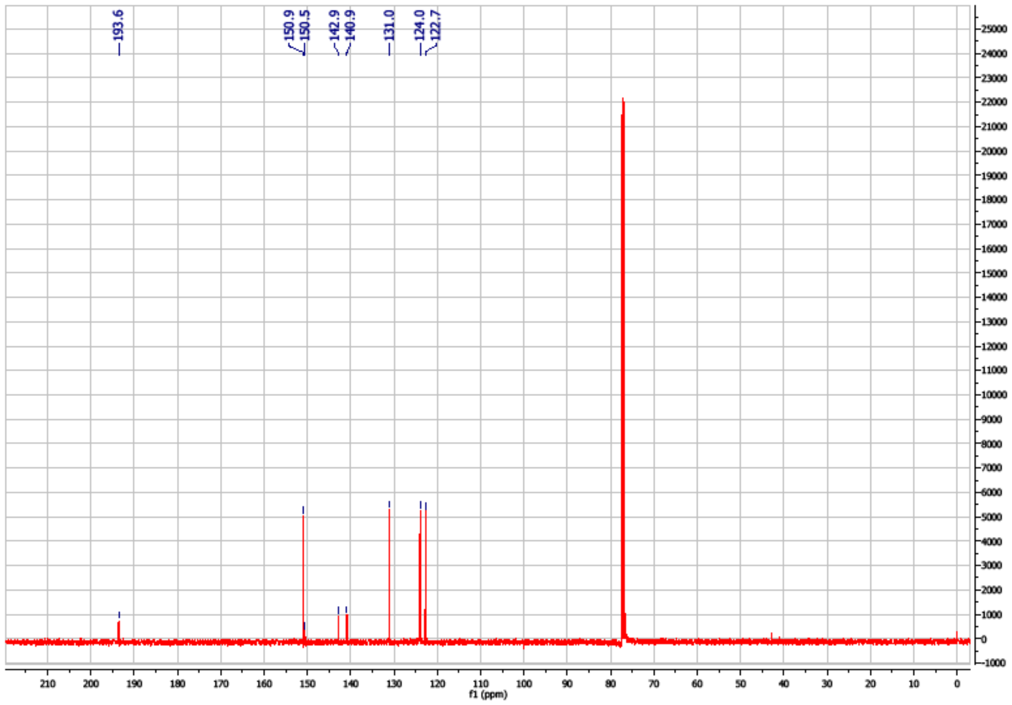

(4-Bromophenyl)(pyridin-4-yl)methanone

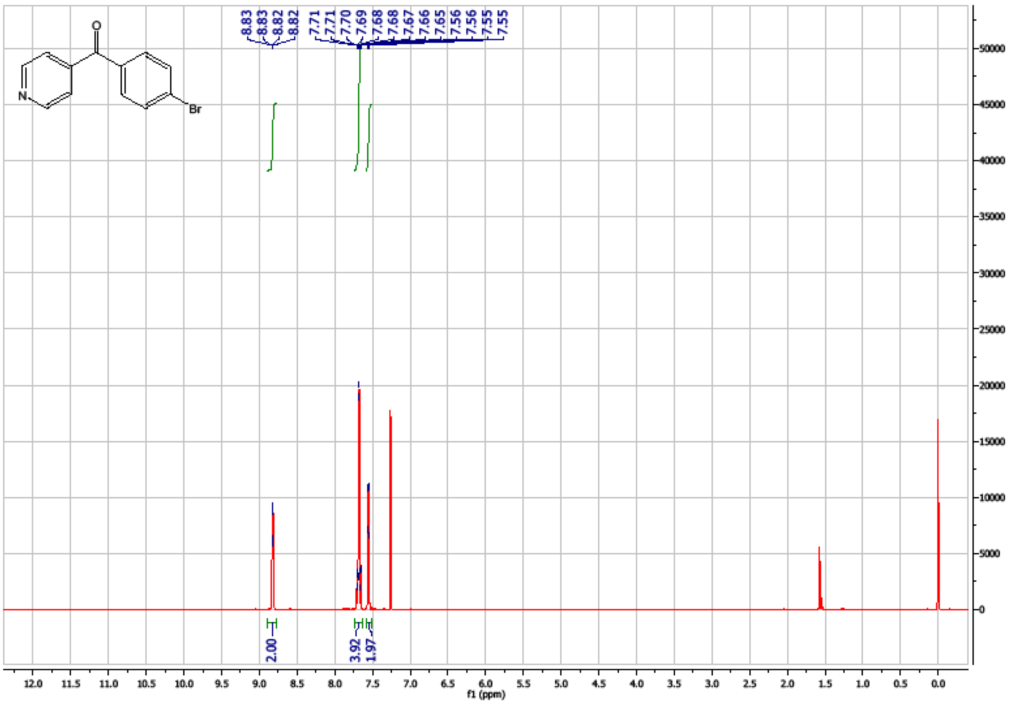

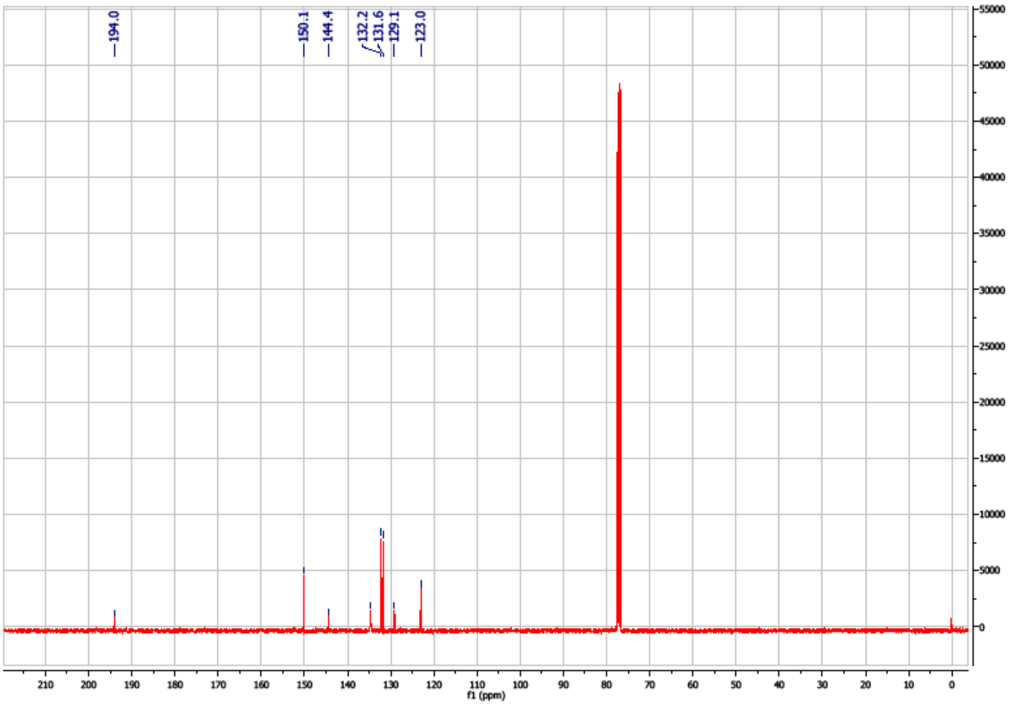

Iodophenyl)(pyridin-4-yl)methanone

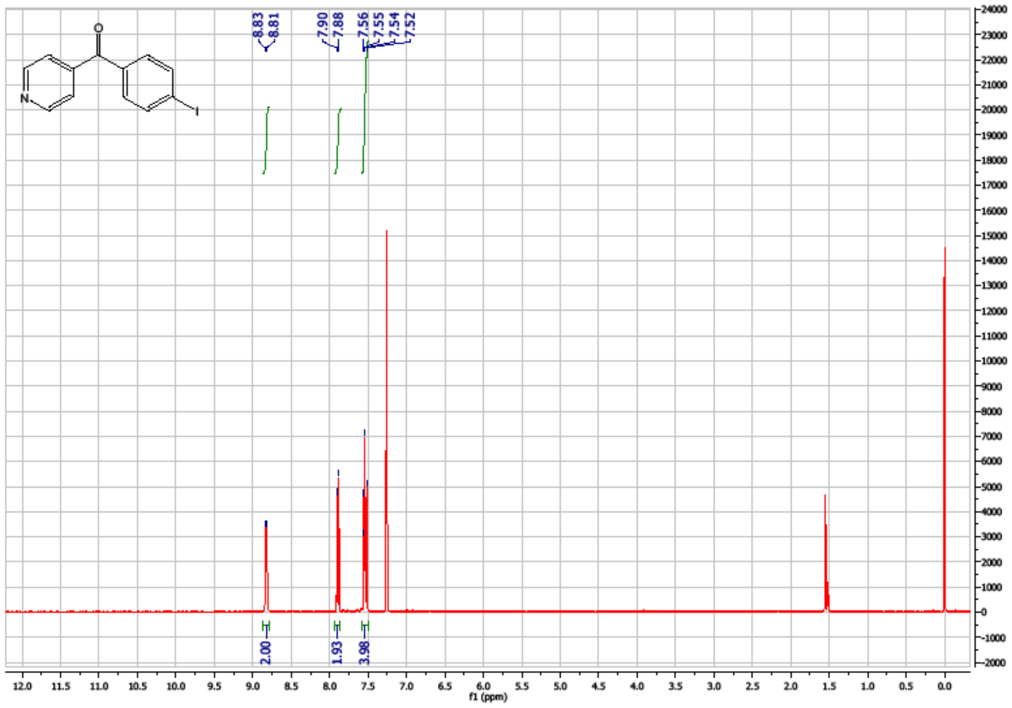


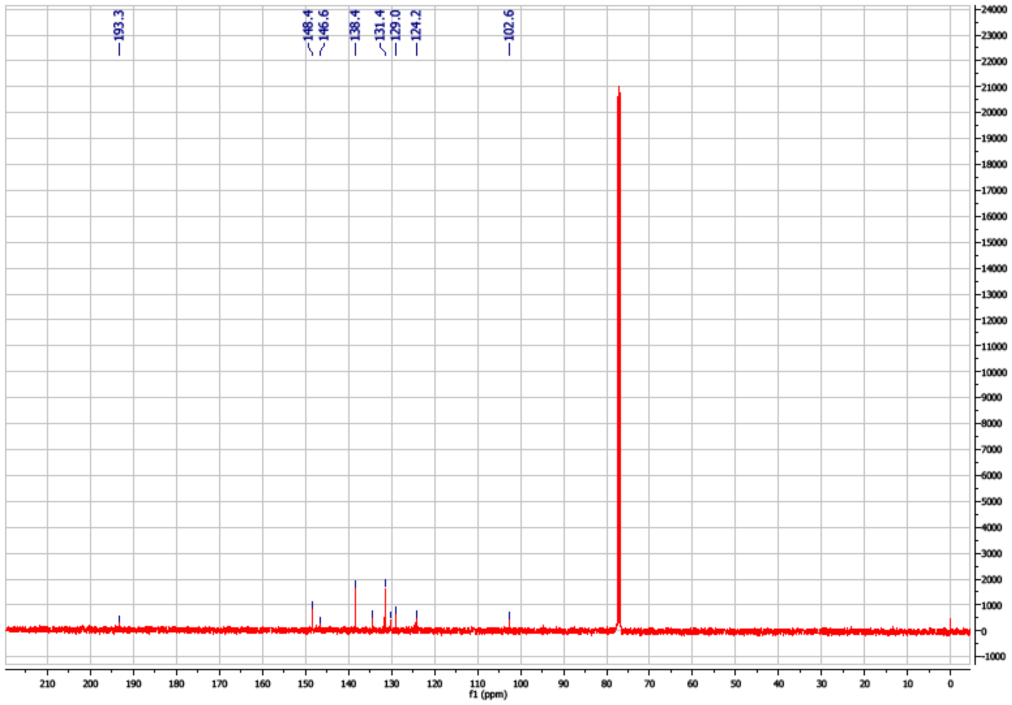

(4-Aminophenyl)(pyridin-4-yl)methanone

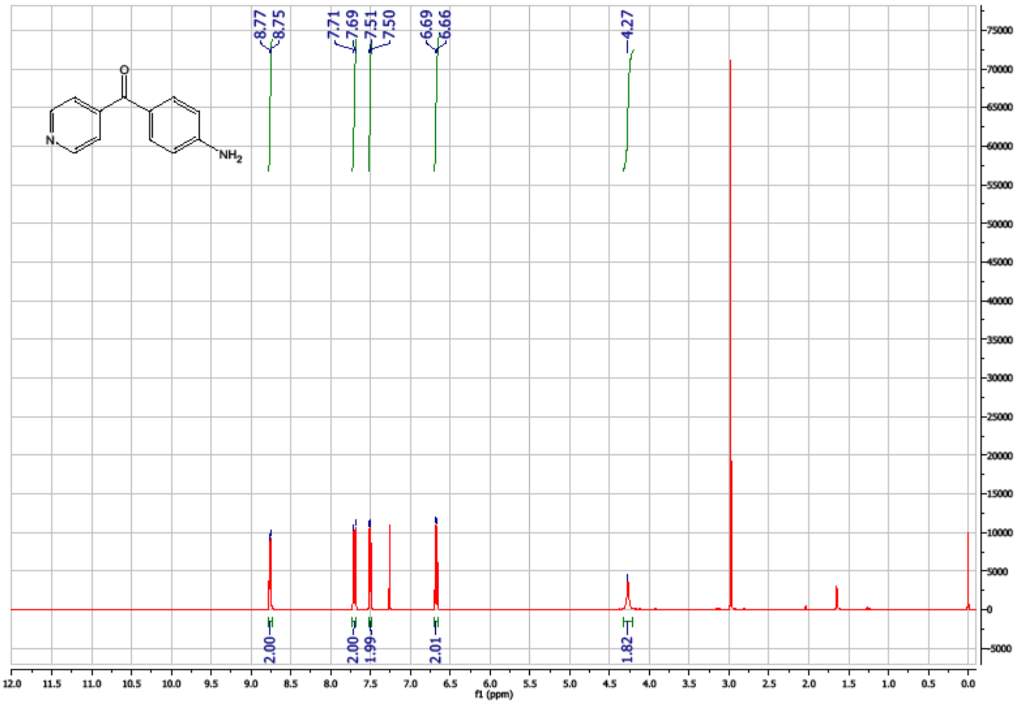


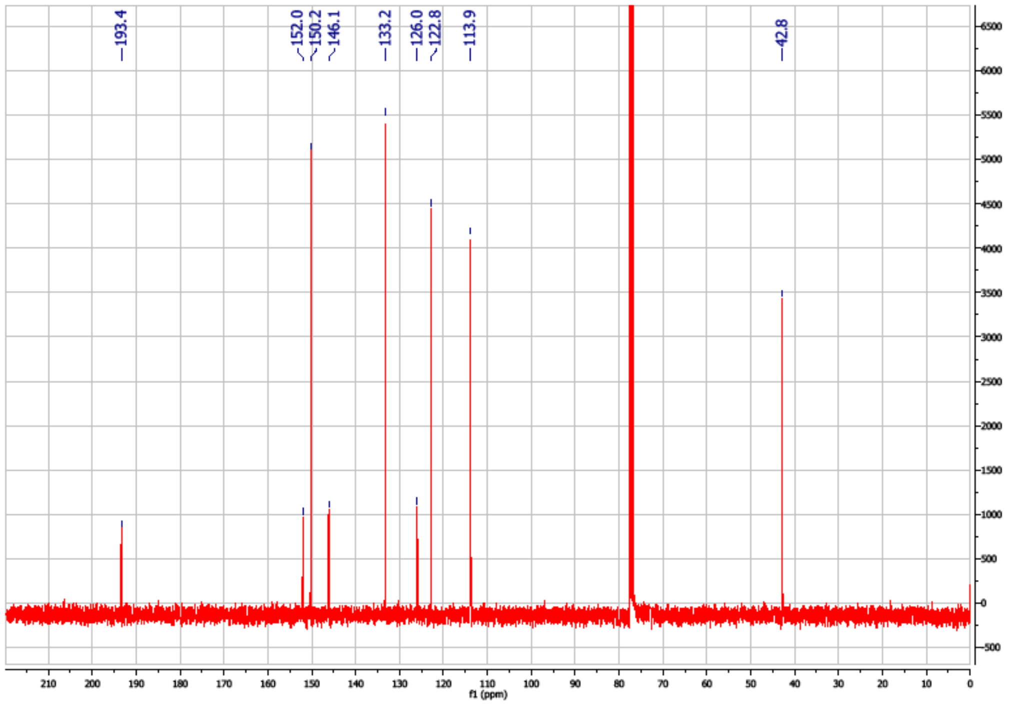

Phenyl(quinolin-2-yl)methanone

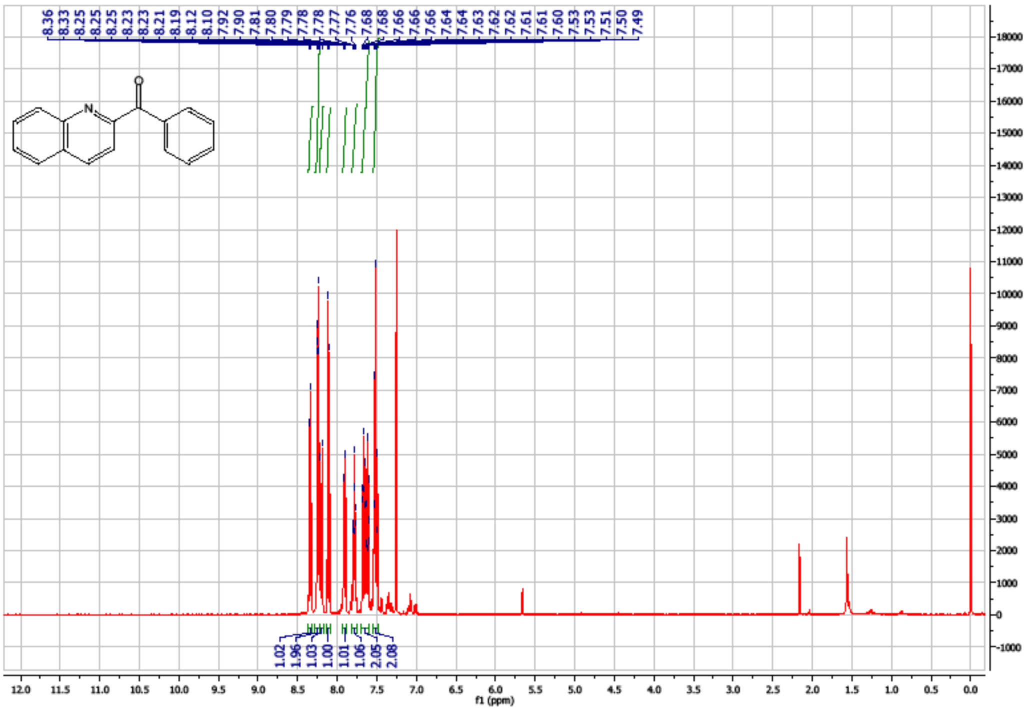


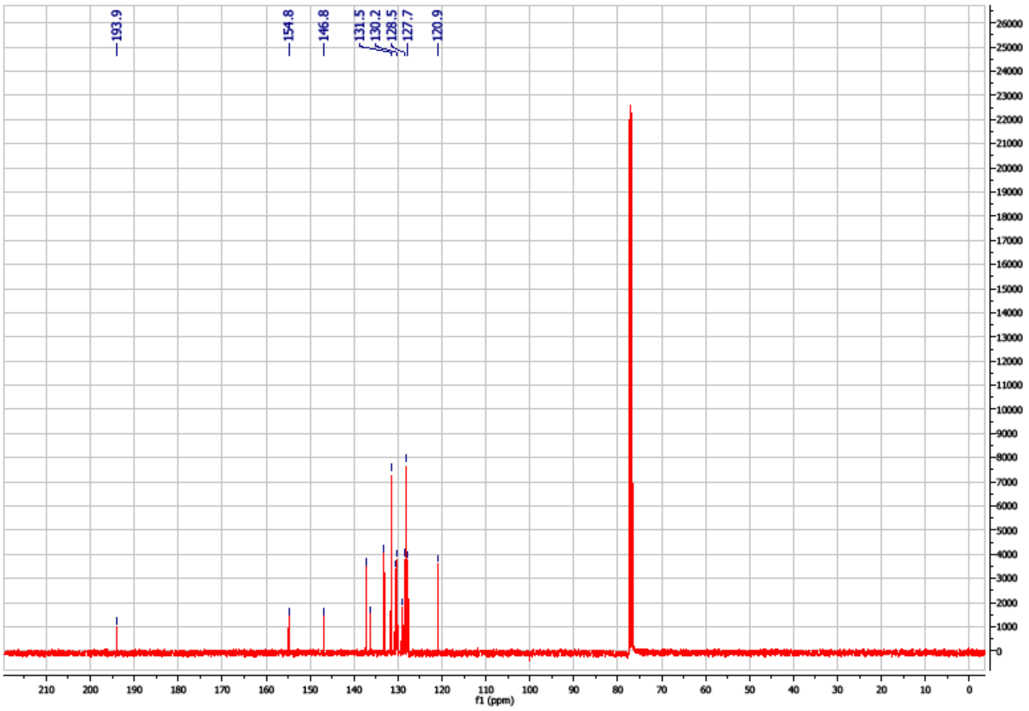

Phenyl(quinolin-4-yl)methanone

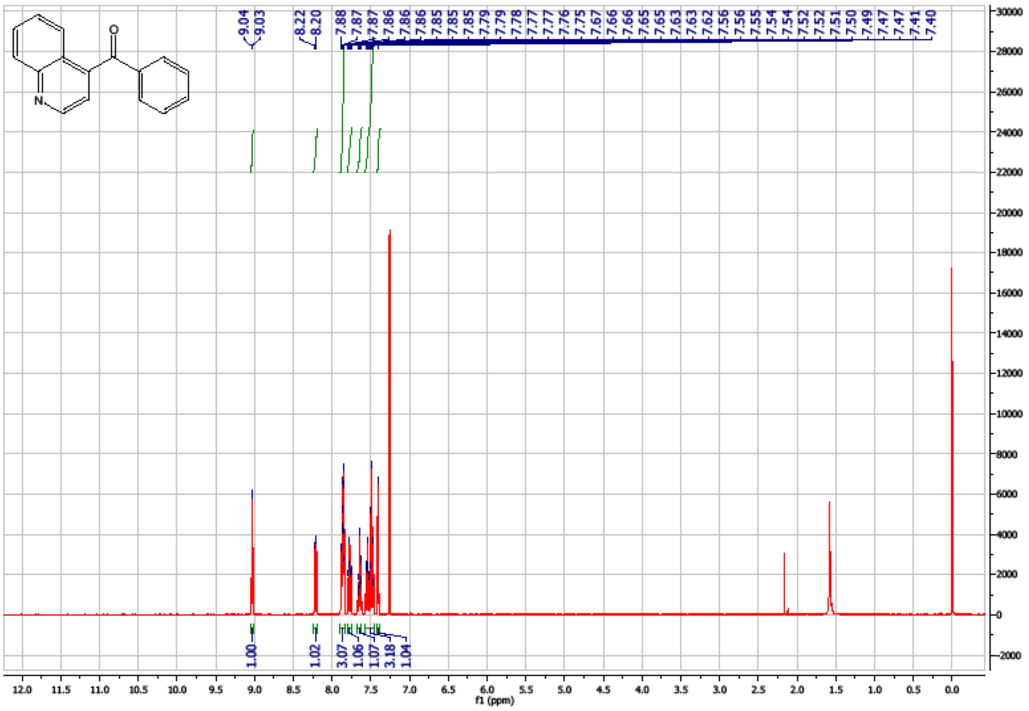

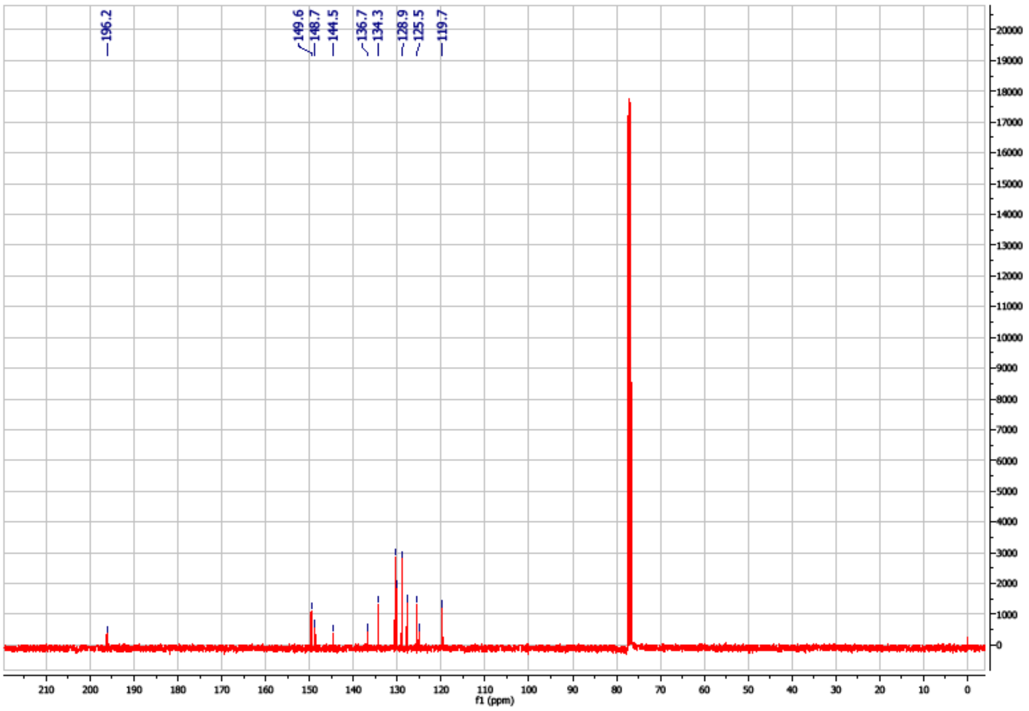

(2,8-Bis(trifluoromethyl)quinolin-4-yl)(pyridin-2-yl)methanone

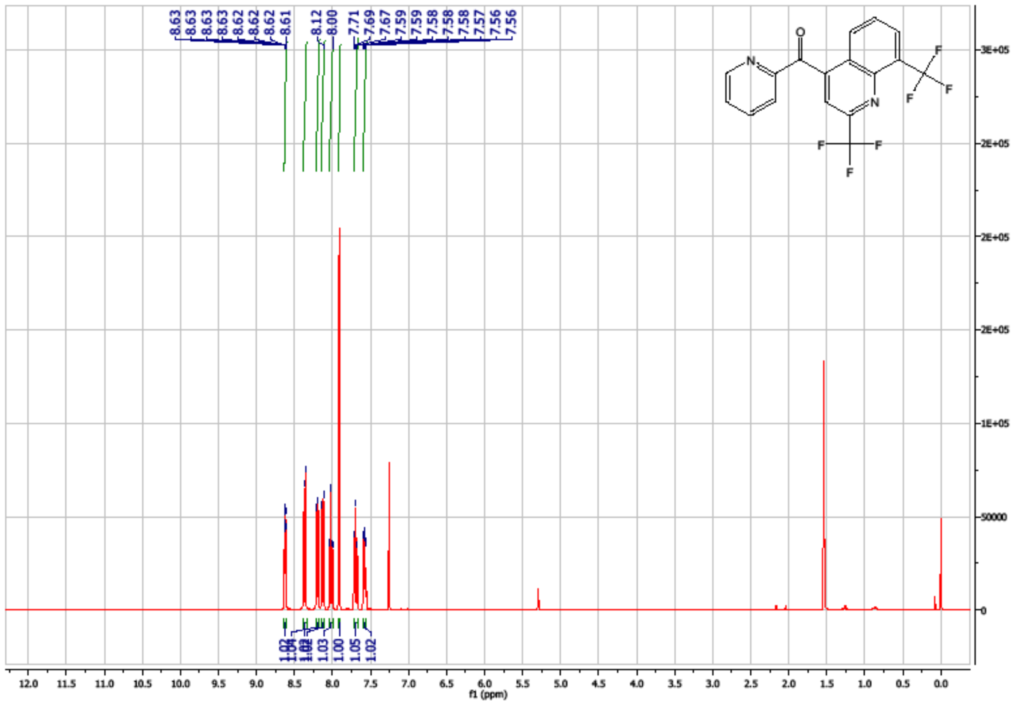

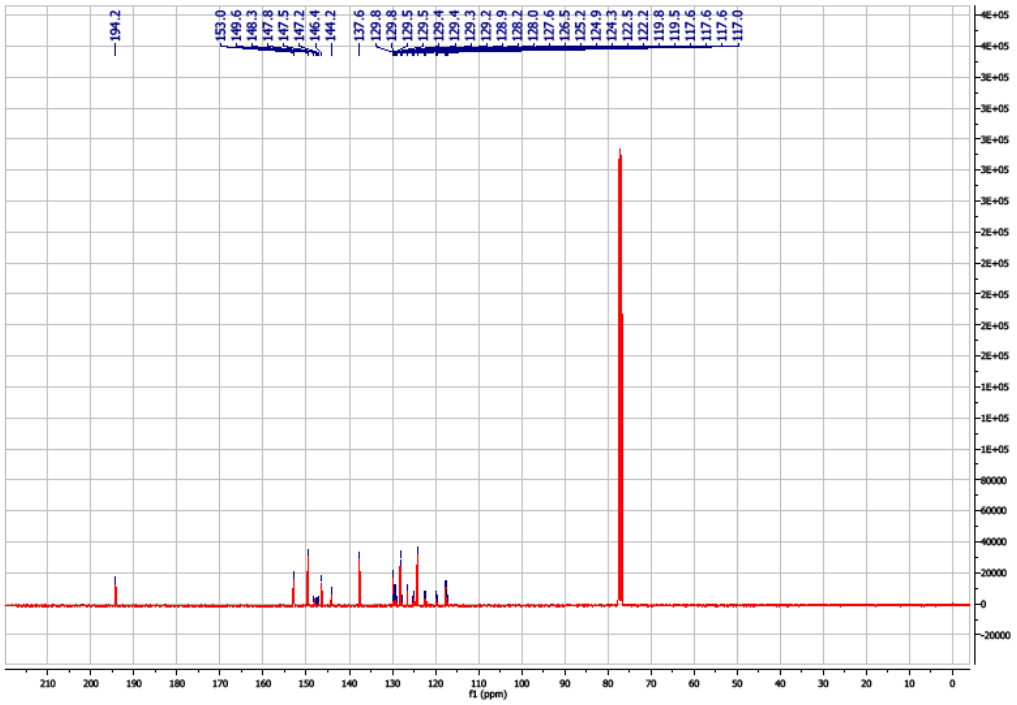

(4-Methylphenyl)(6-methylpyrazin-2-yl)methanone

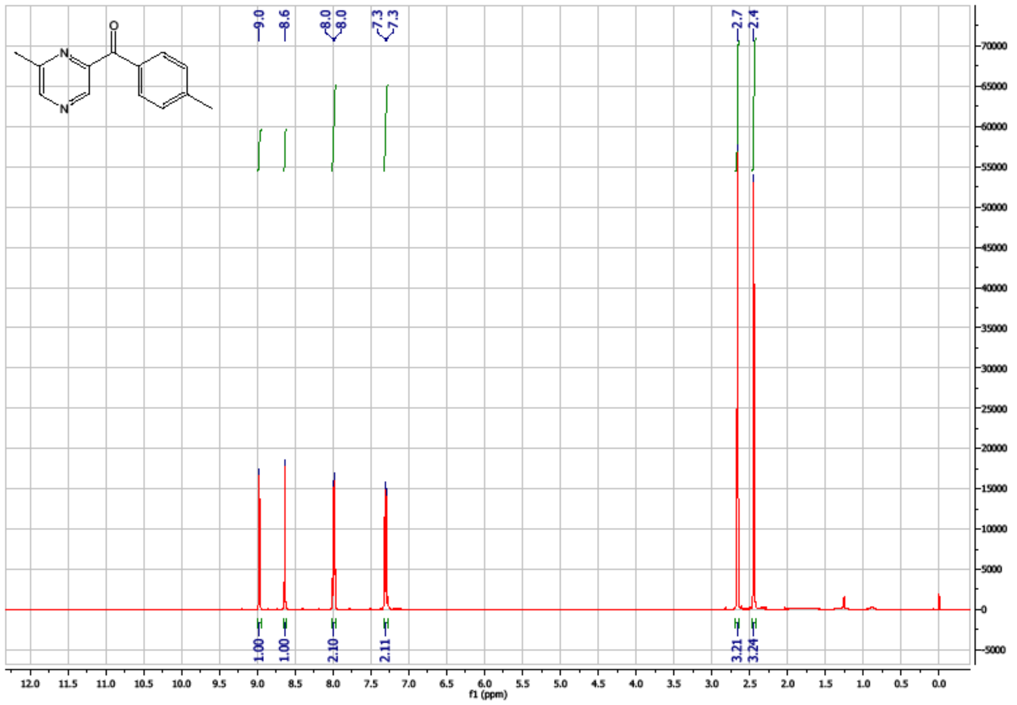


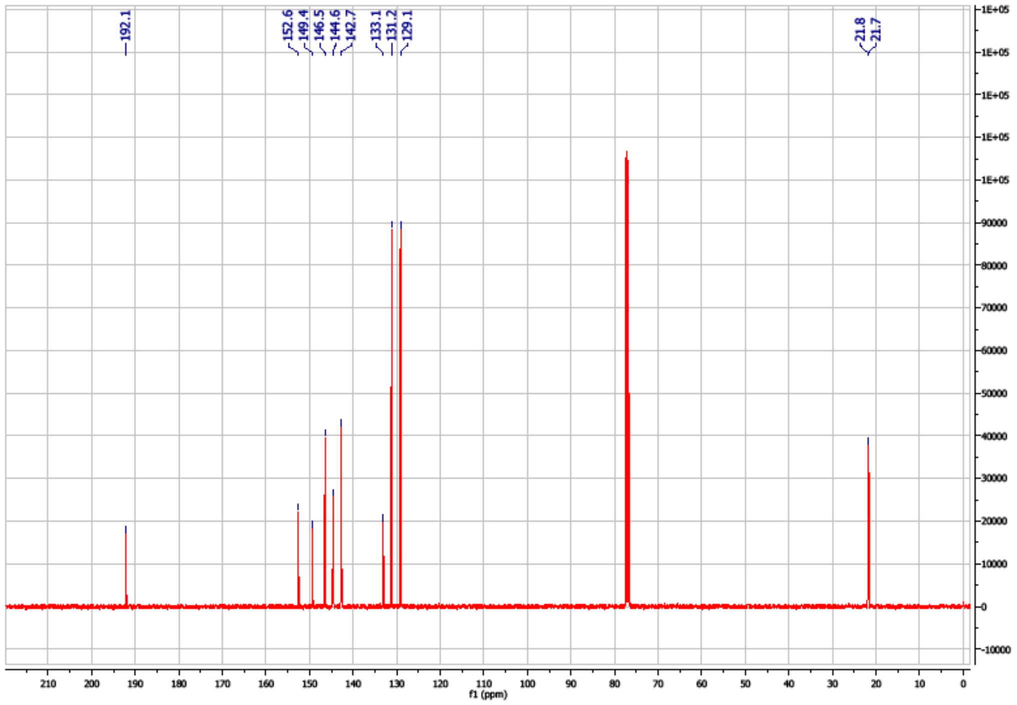

6-(4-Methylbenzoyl)pyrazine-2-carbaldehyde

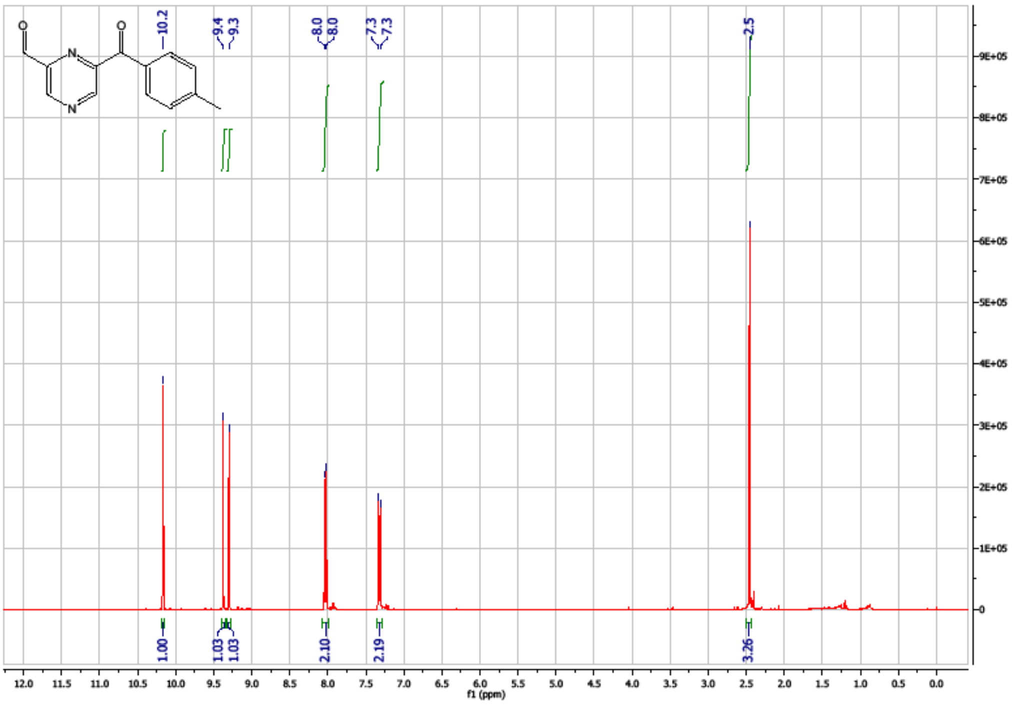


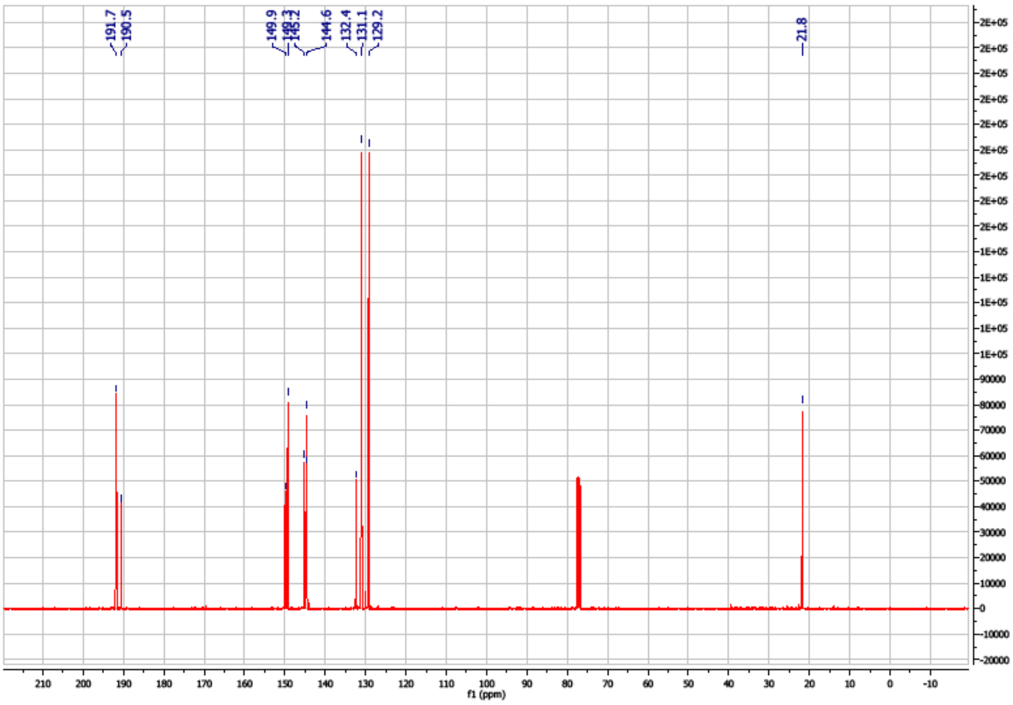

(4-Chlorophenyl)(pyrazin-2-yl)methanone

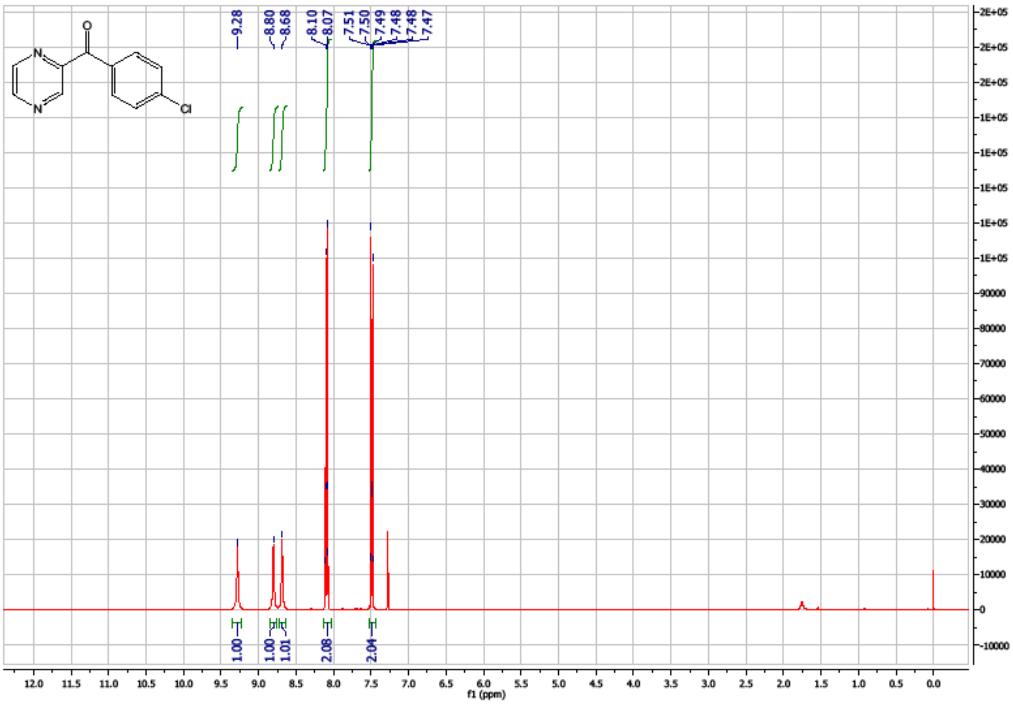


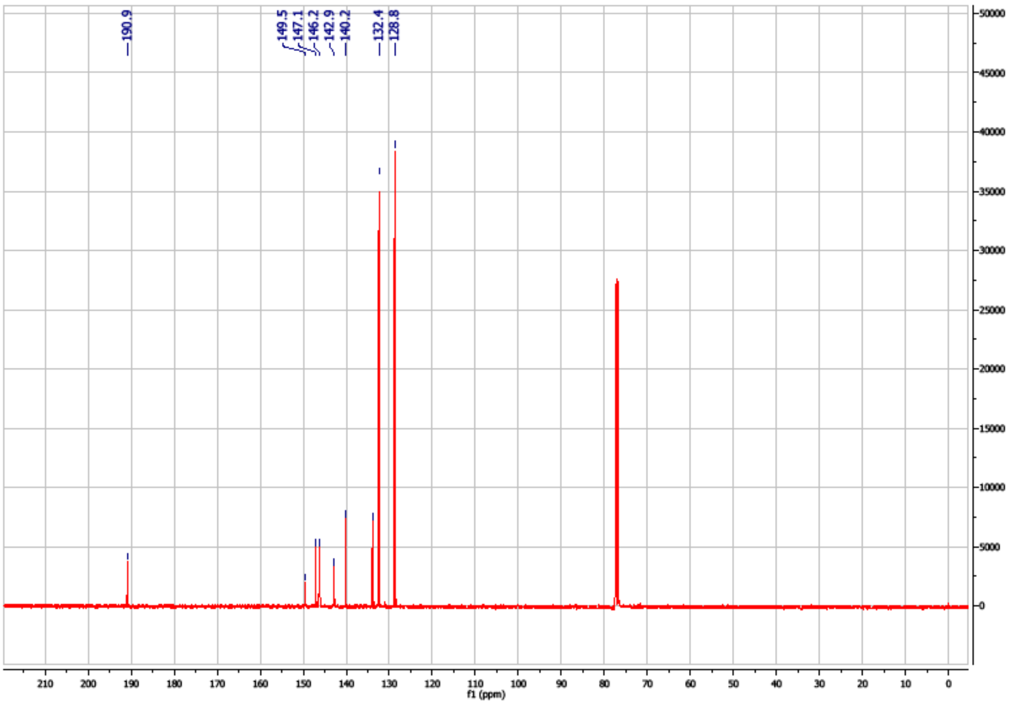

(4-Chlorophenyl)(pyrimidin-2-yl)methanone

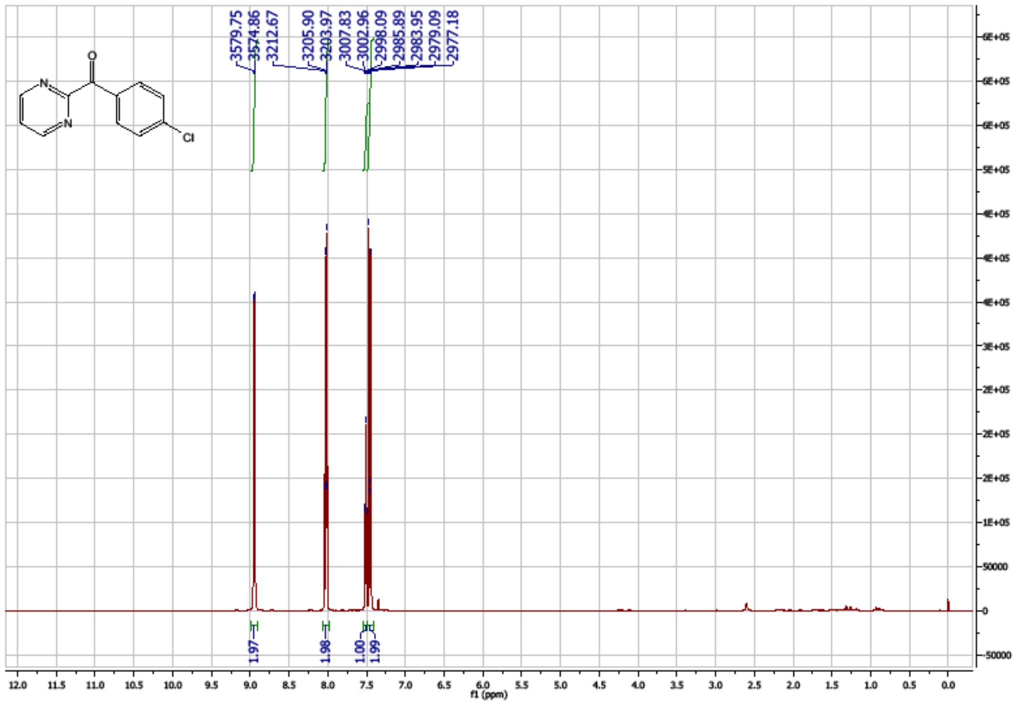


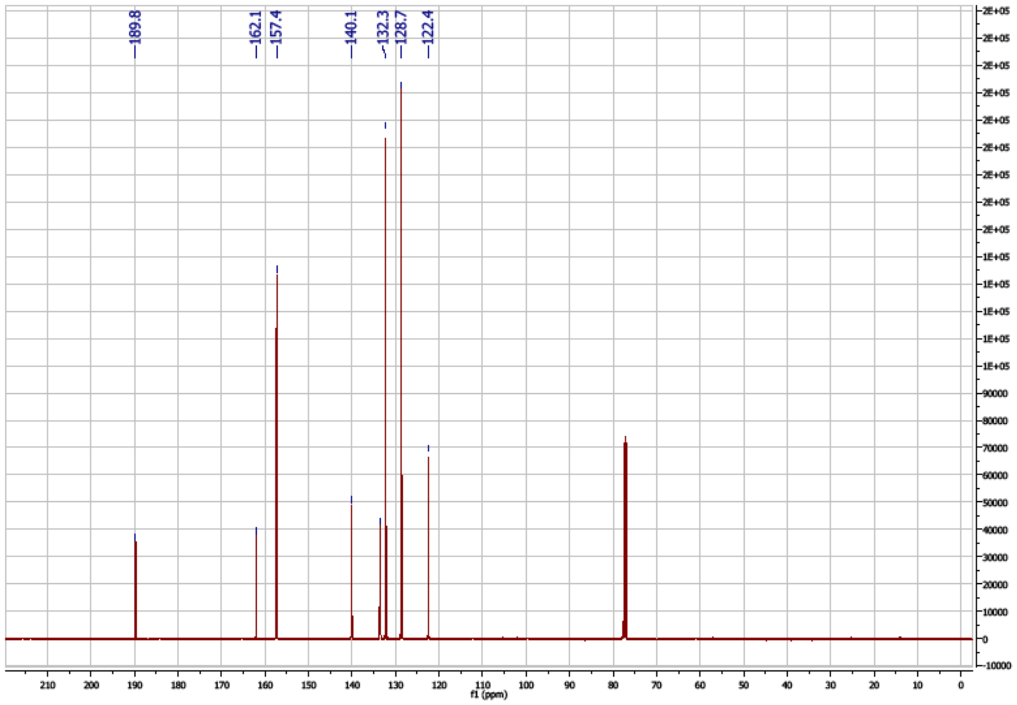

6-Benzylnicotinonitrile

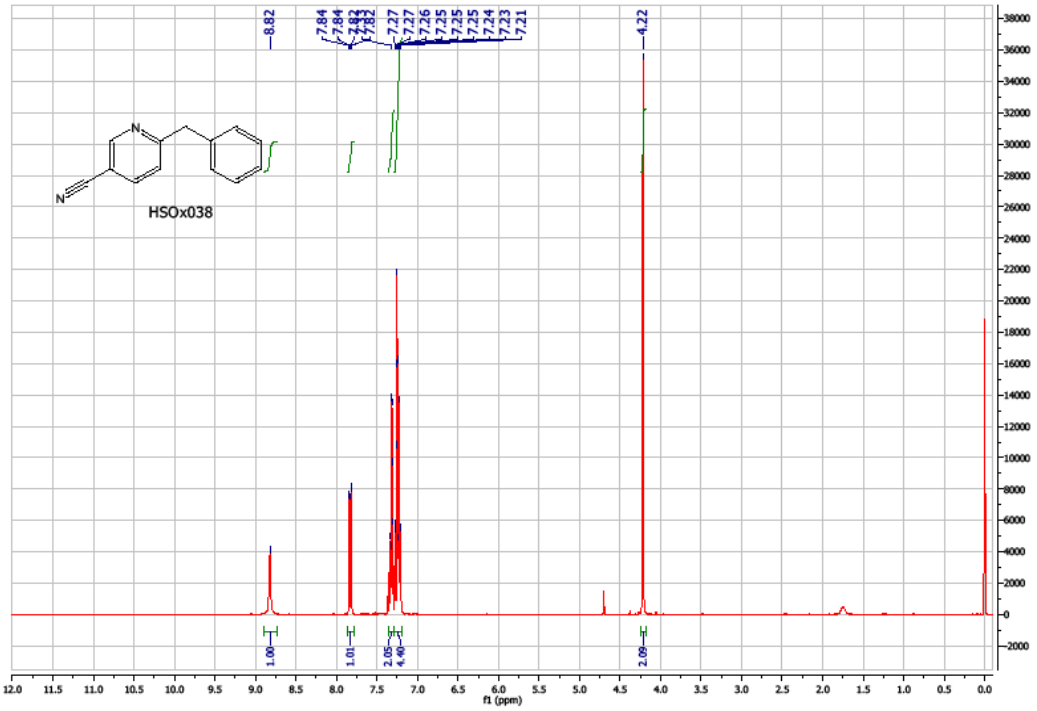


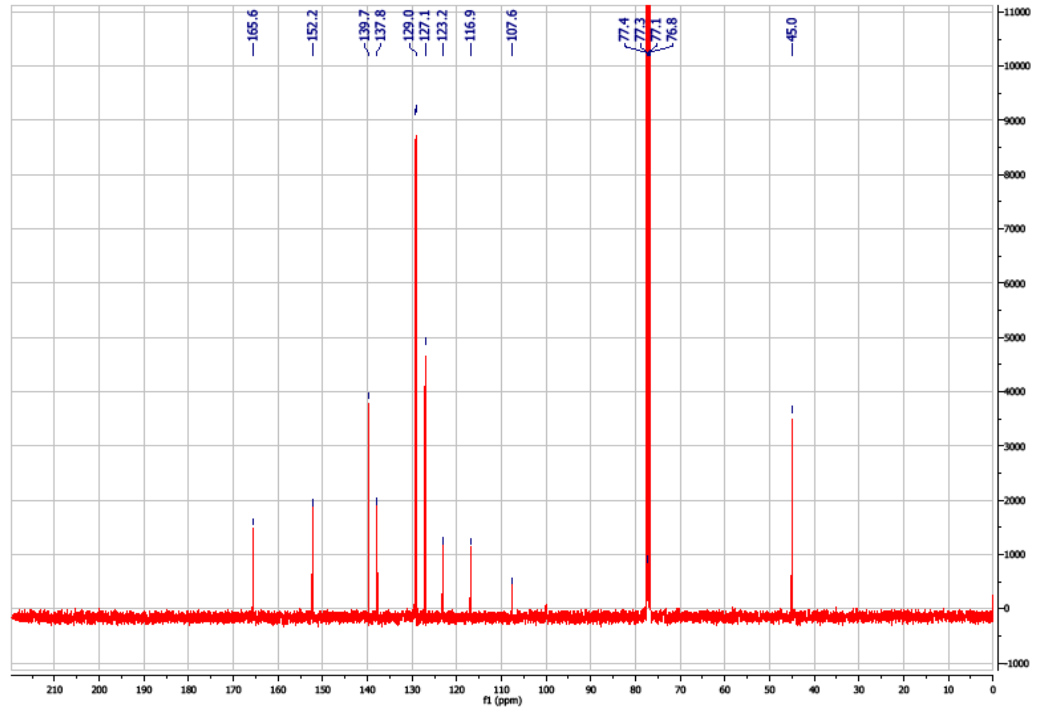

2-Benzyl-5-methylpyridine

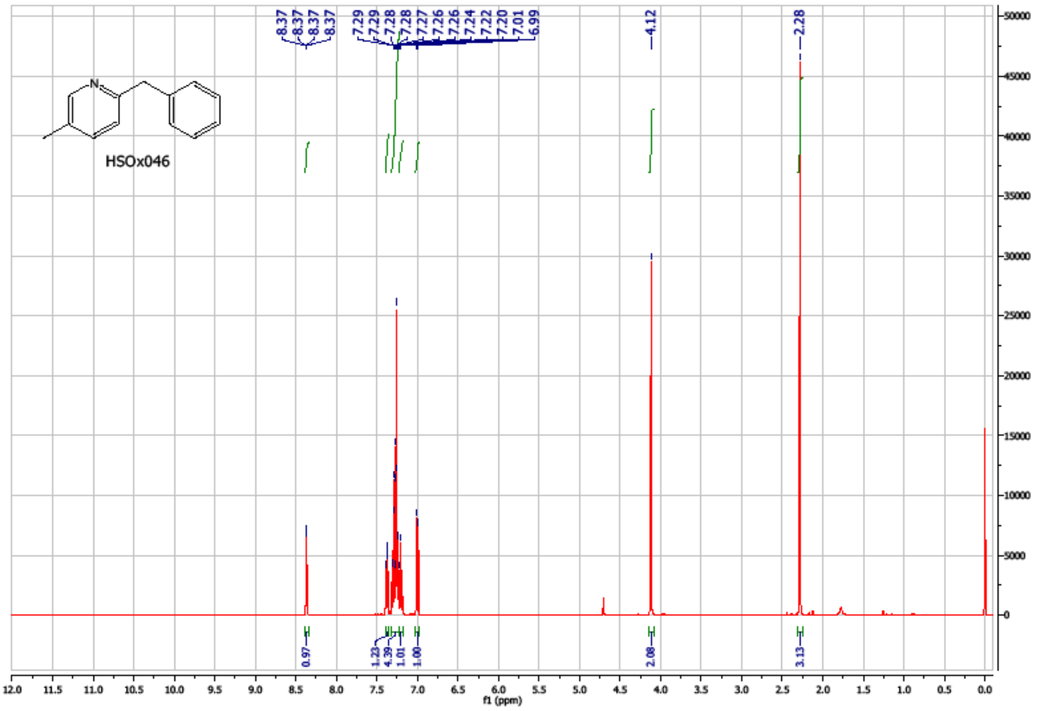


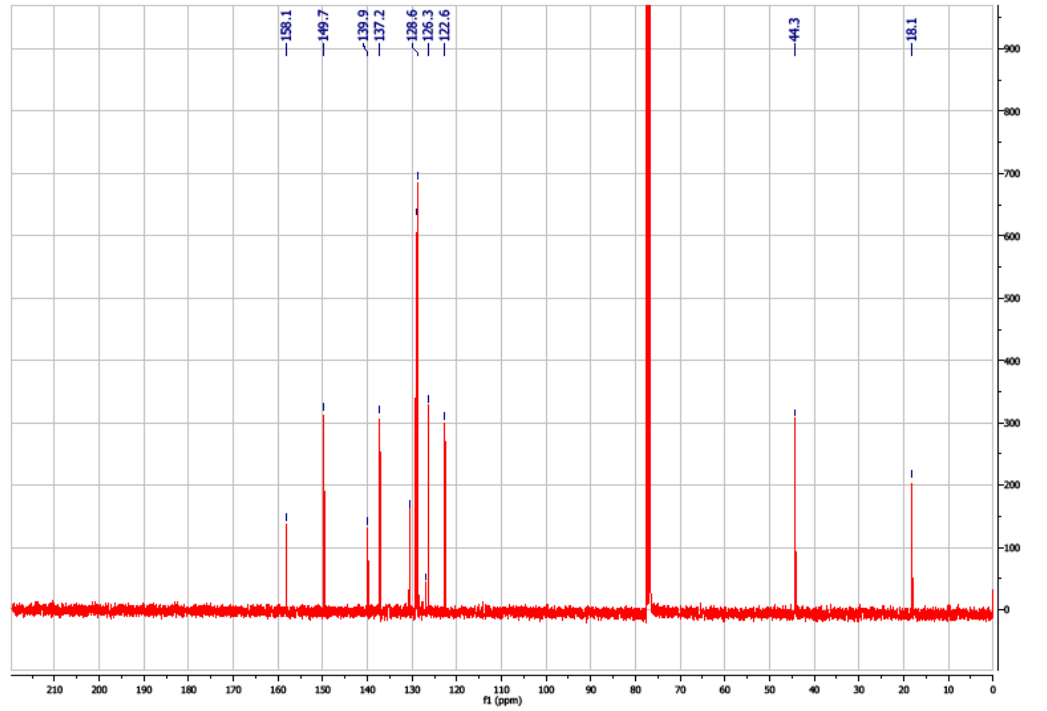

2-Benzyl-5-methoxypyridine

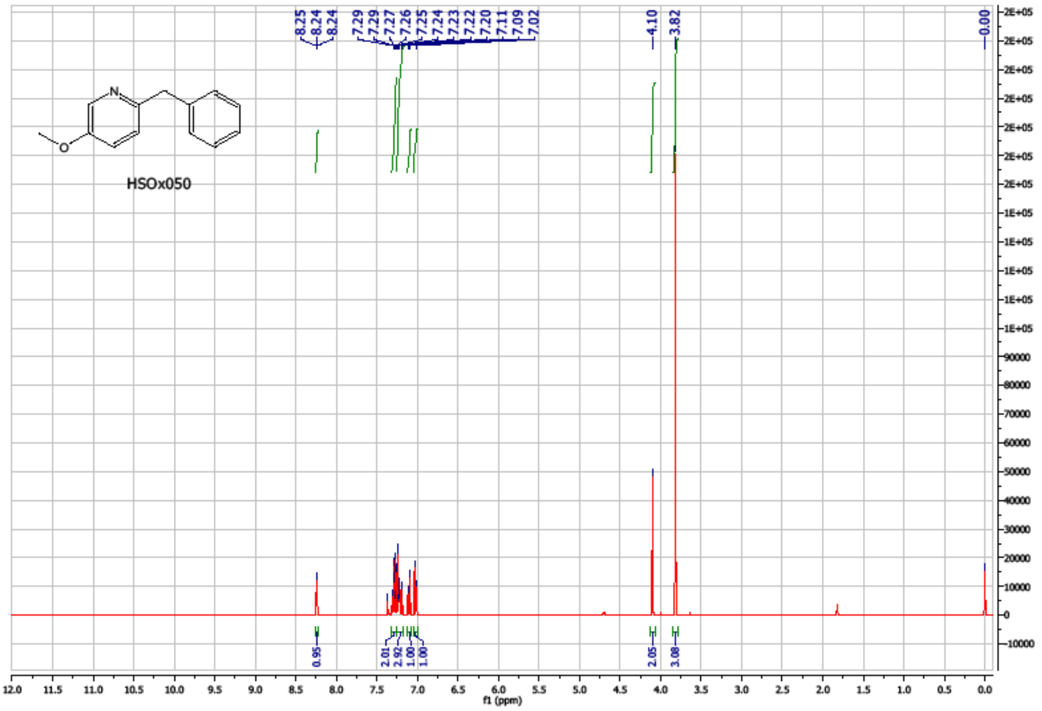


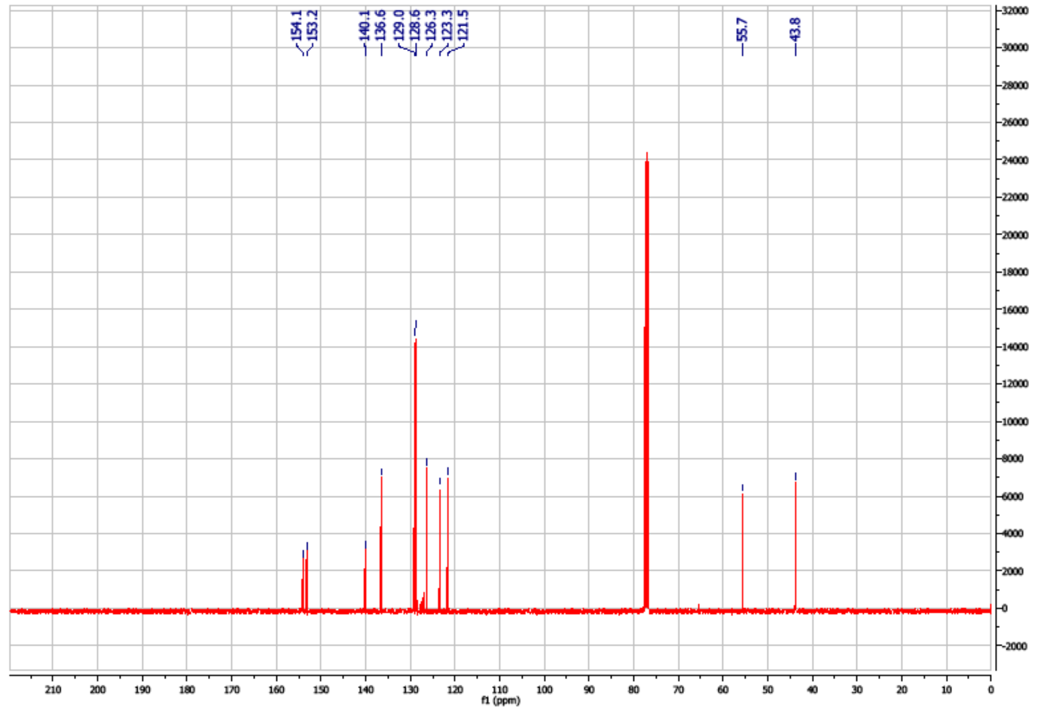

Methyl 6-benzylnicotinate

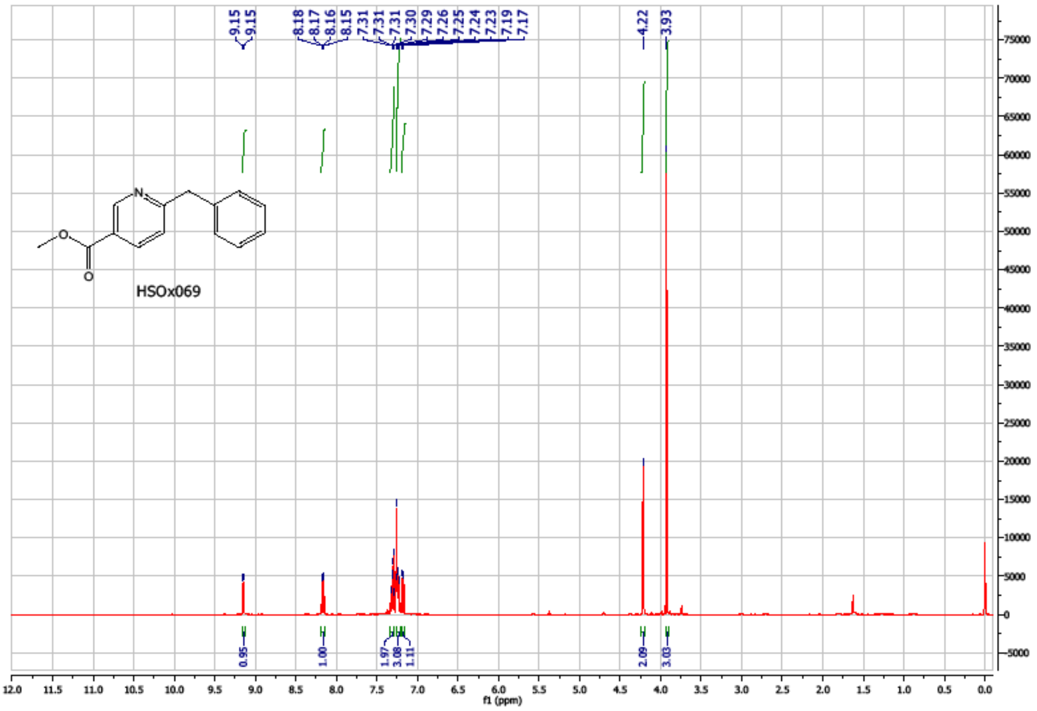


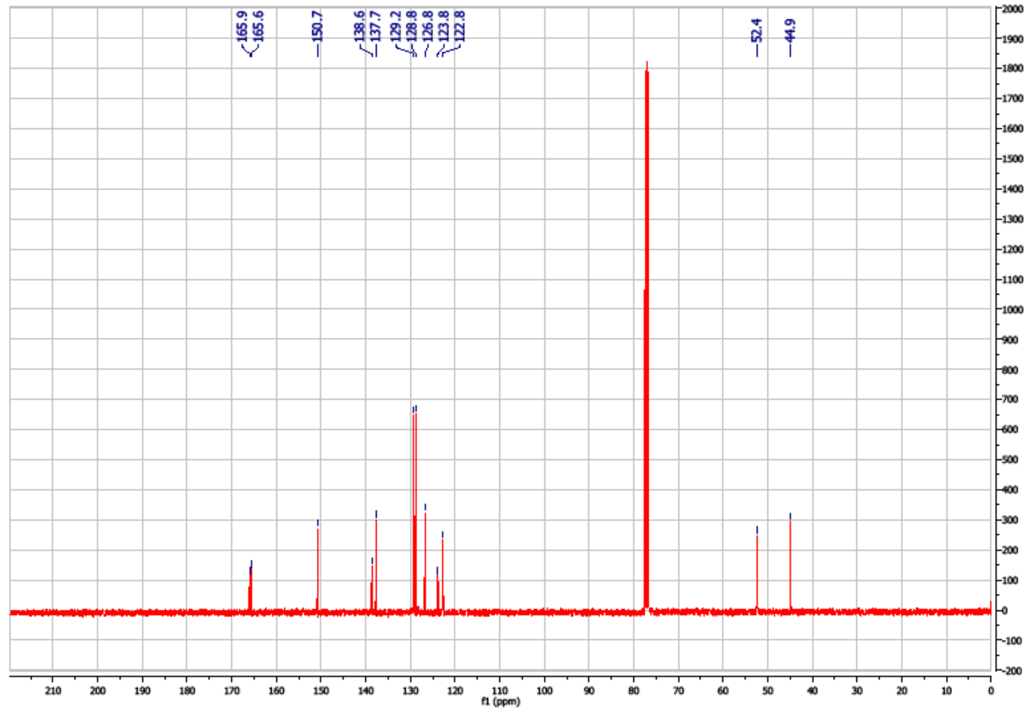

2-Benzyl-4-chloropyridine

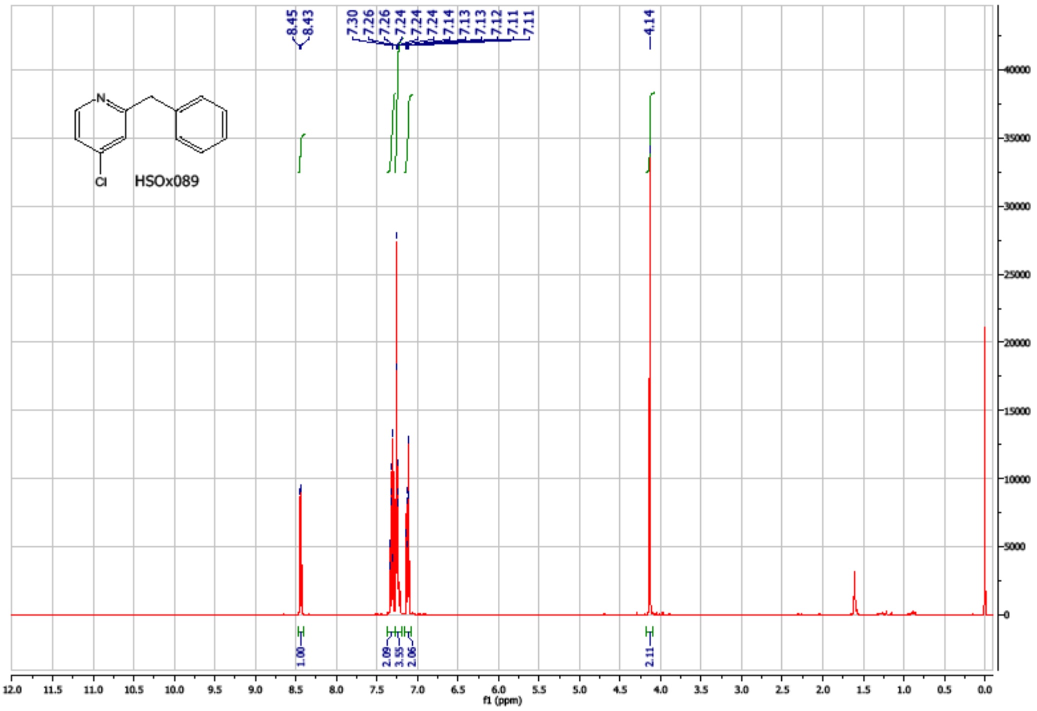


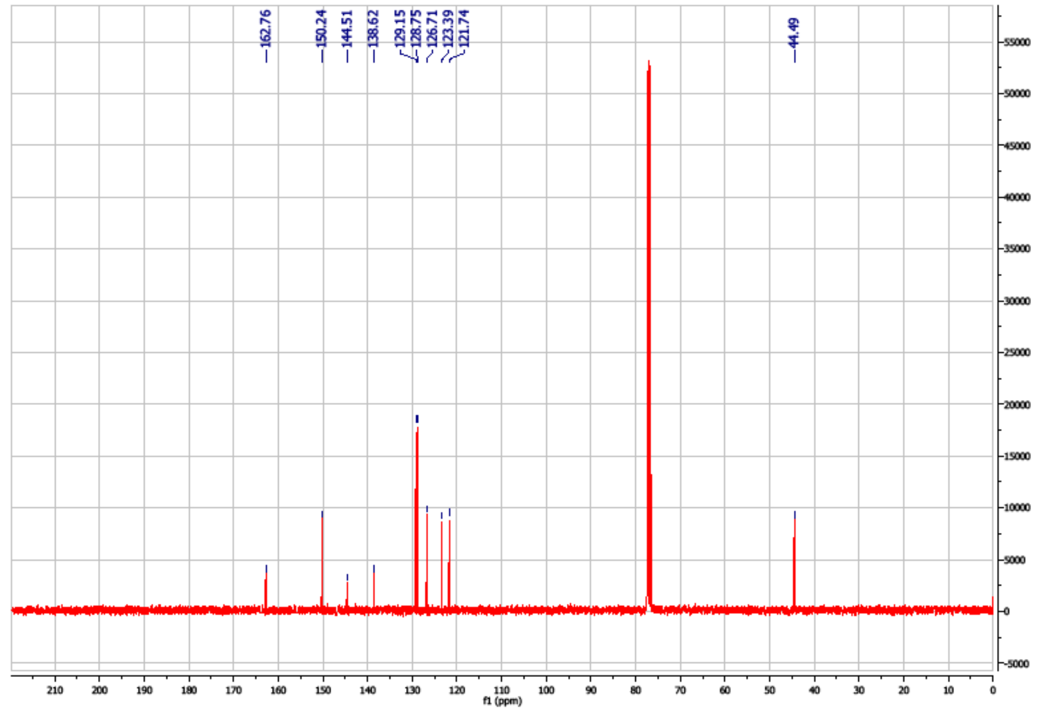

2-Benzyl-3-chloropyridine

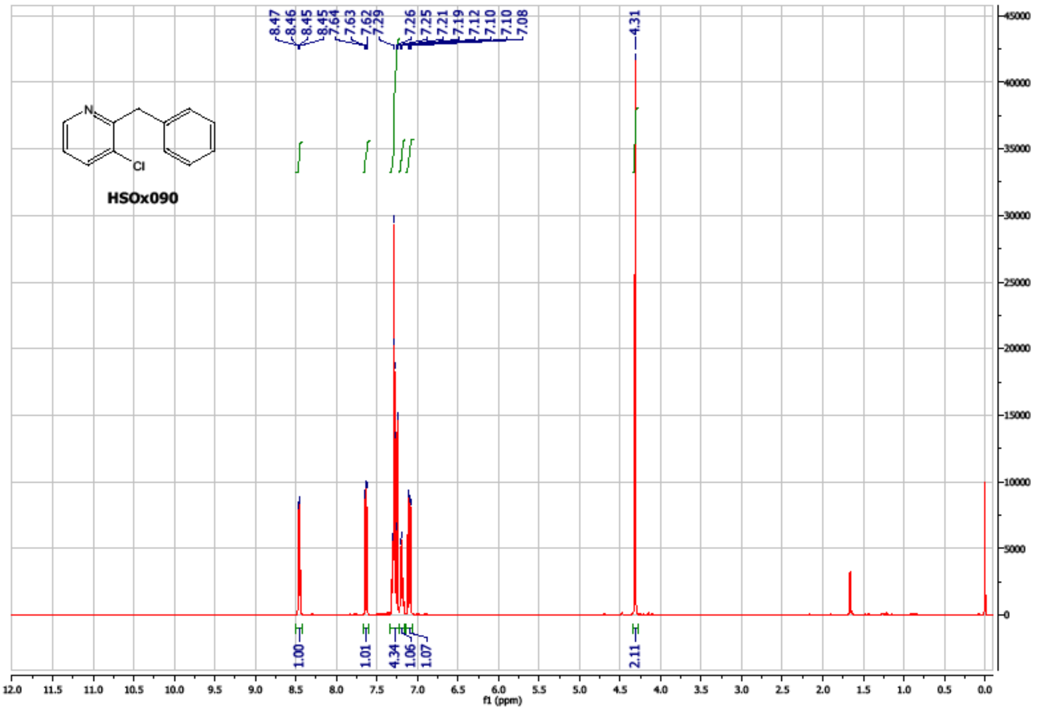


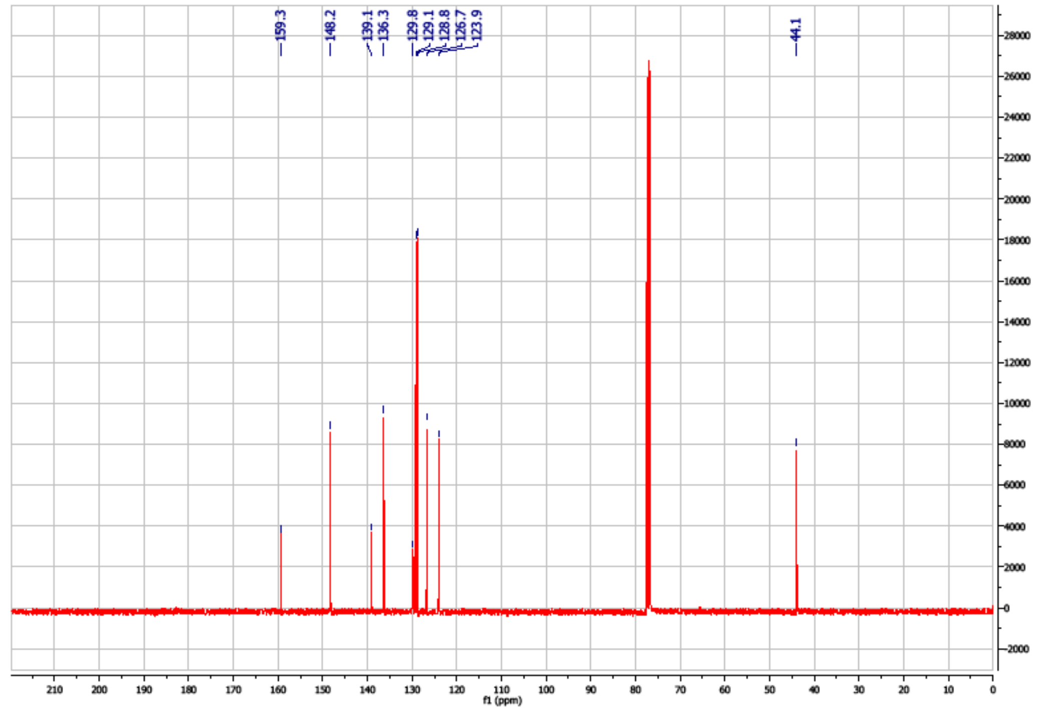

2-Benzyl-5-chloropyridine

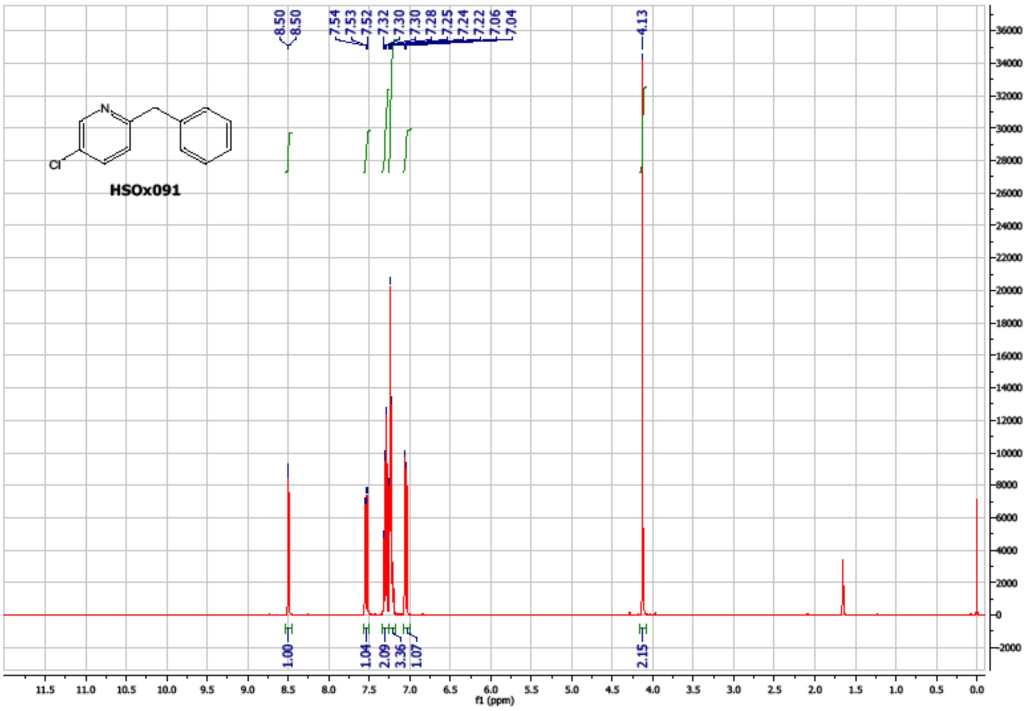


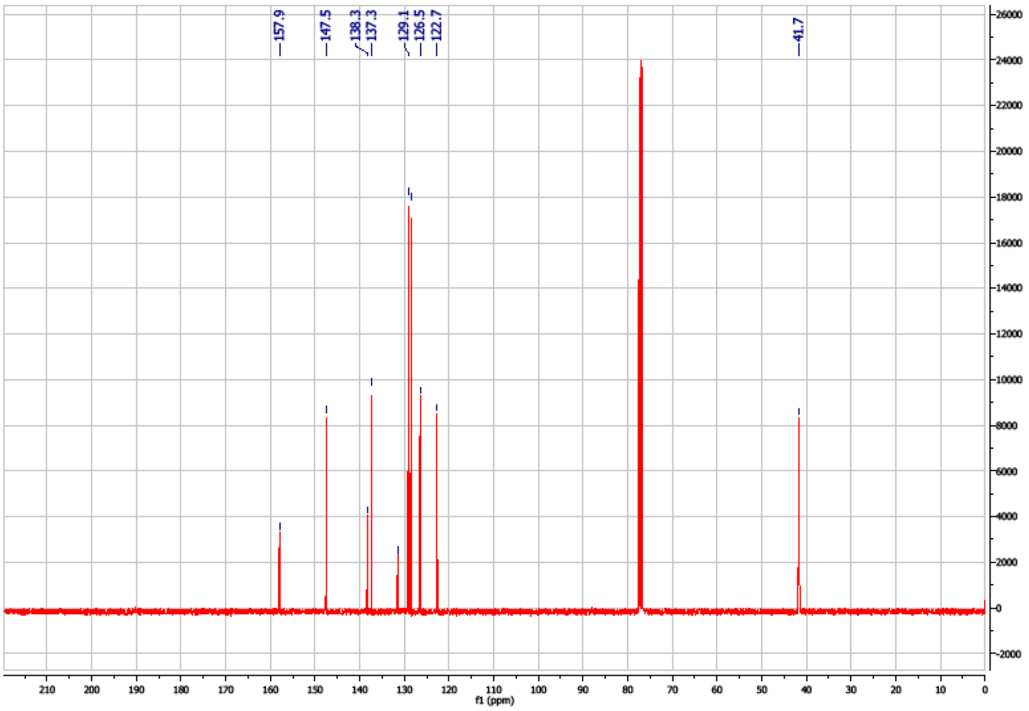

2-Benzyl-6-chloropyridine

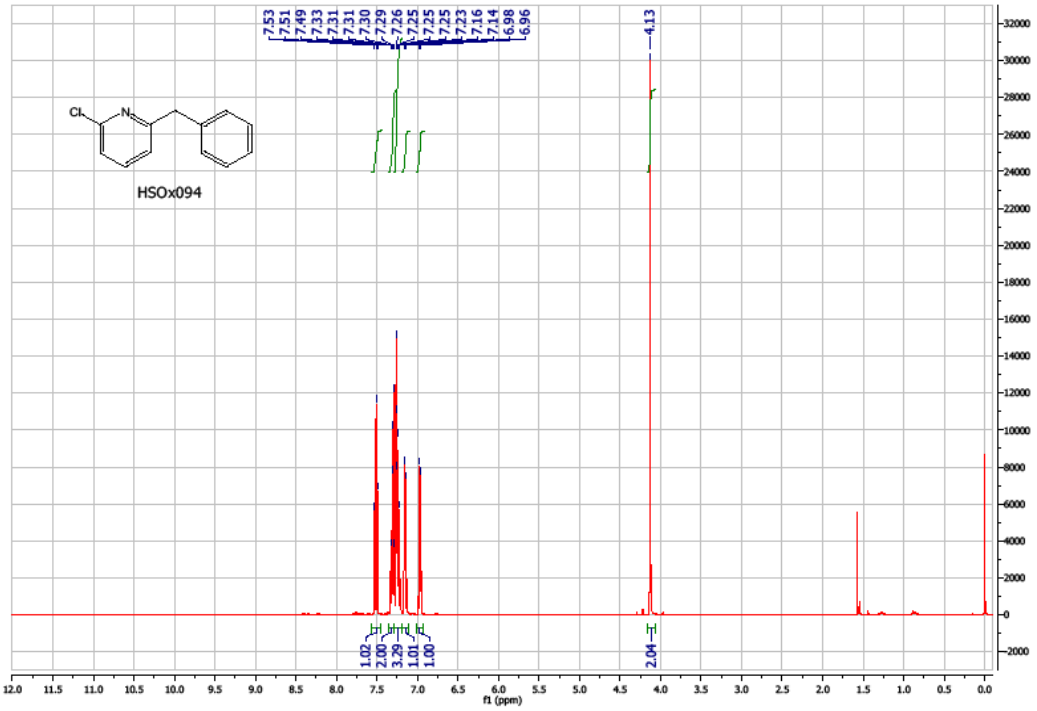


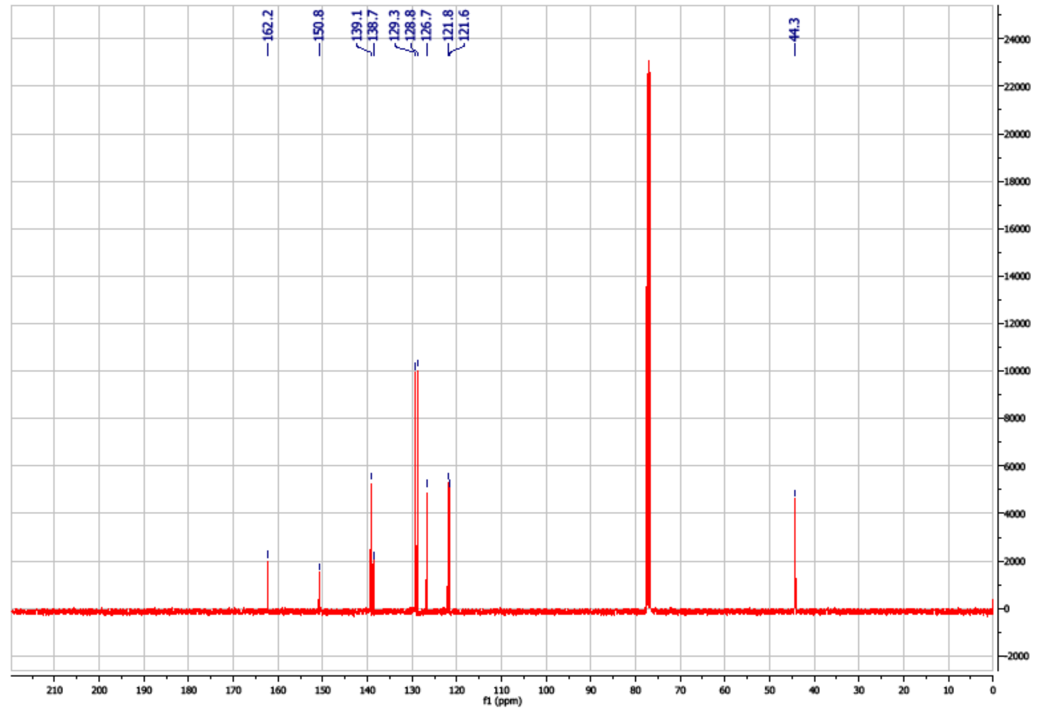

4-Benzyl-2-chloropyrimidine

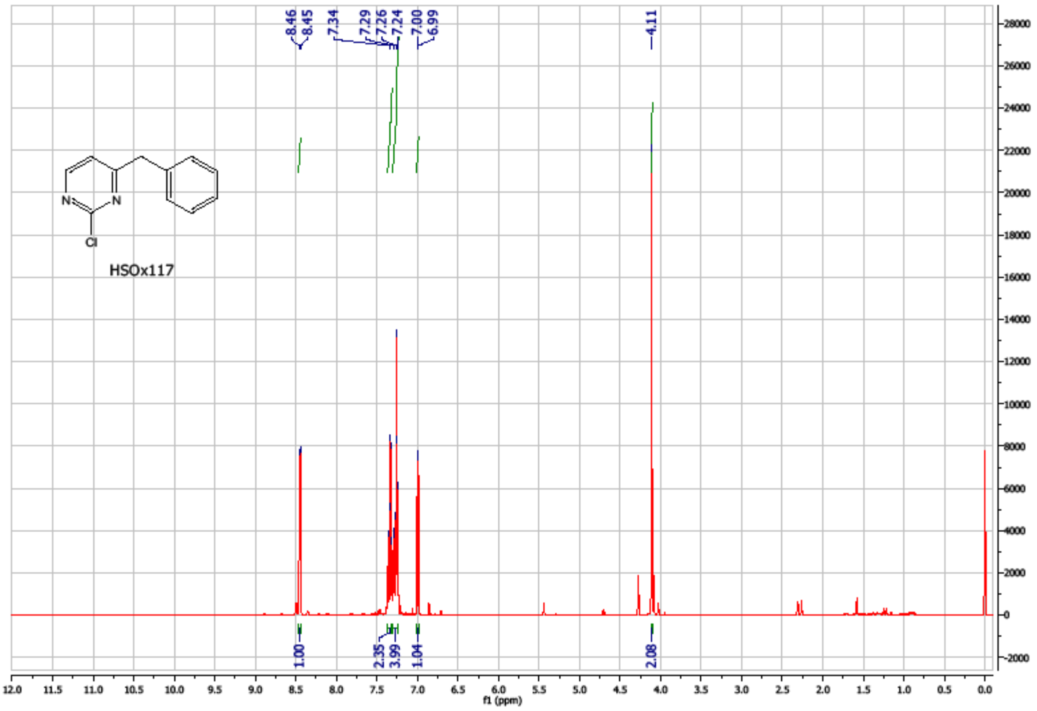


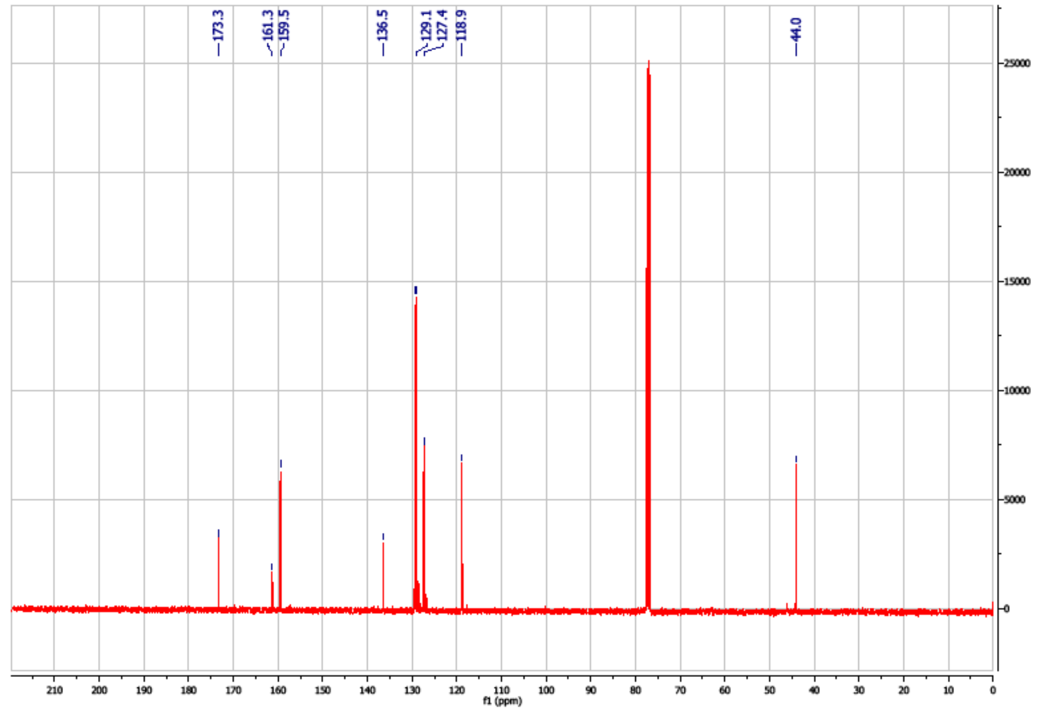

3-Benzyl-6-chloropyridazine

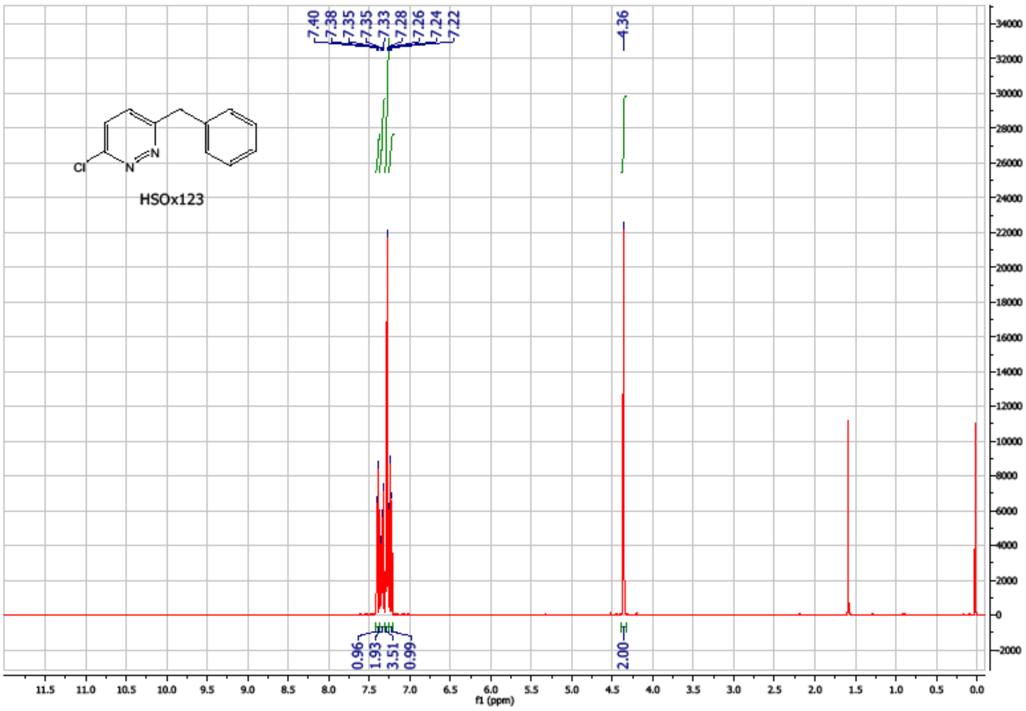


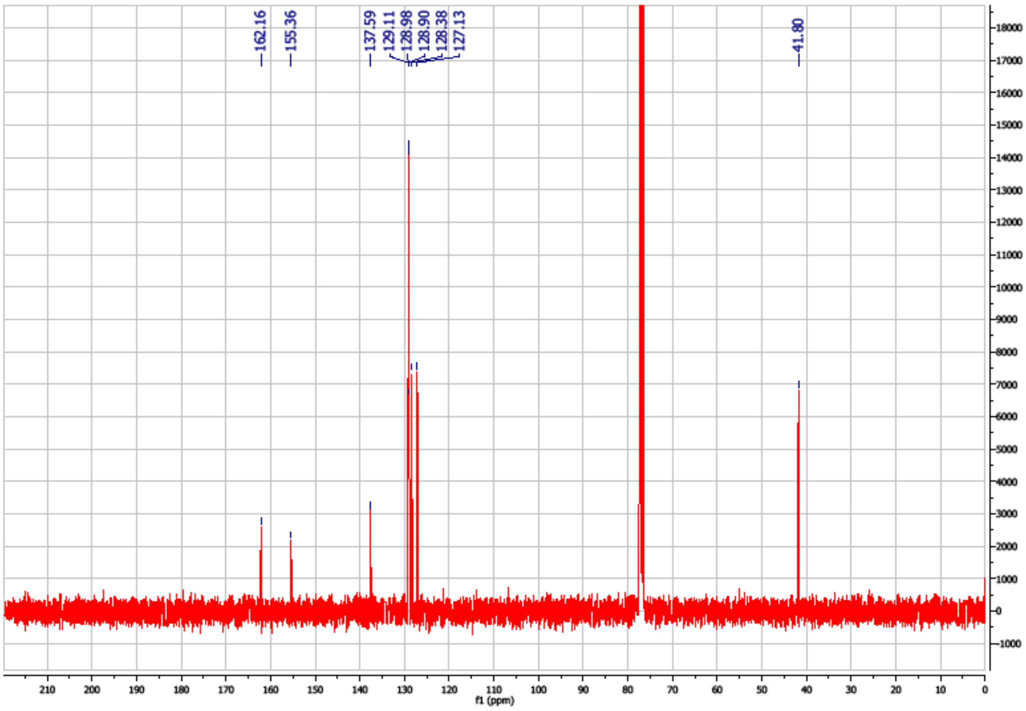

[4-(Methylthio)benzyl]pyridine

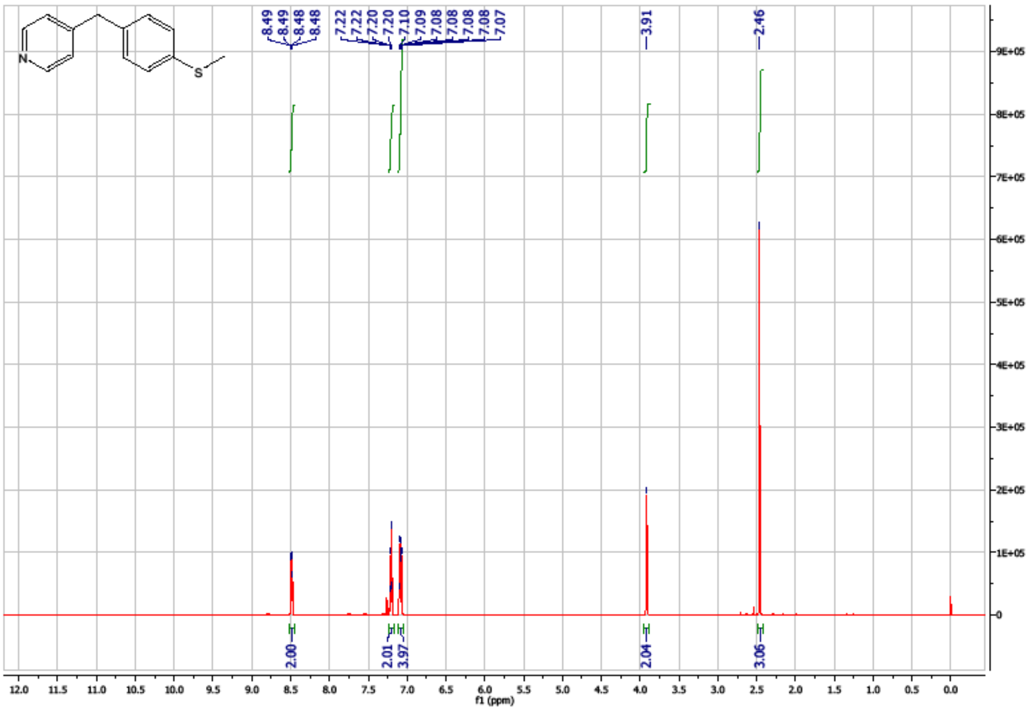


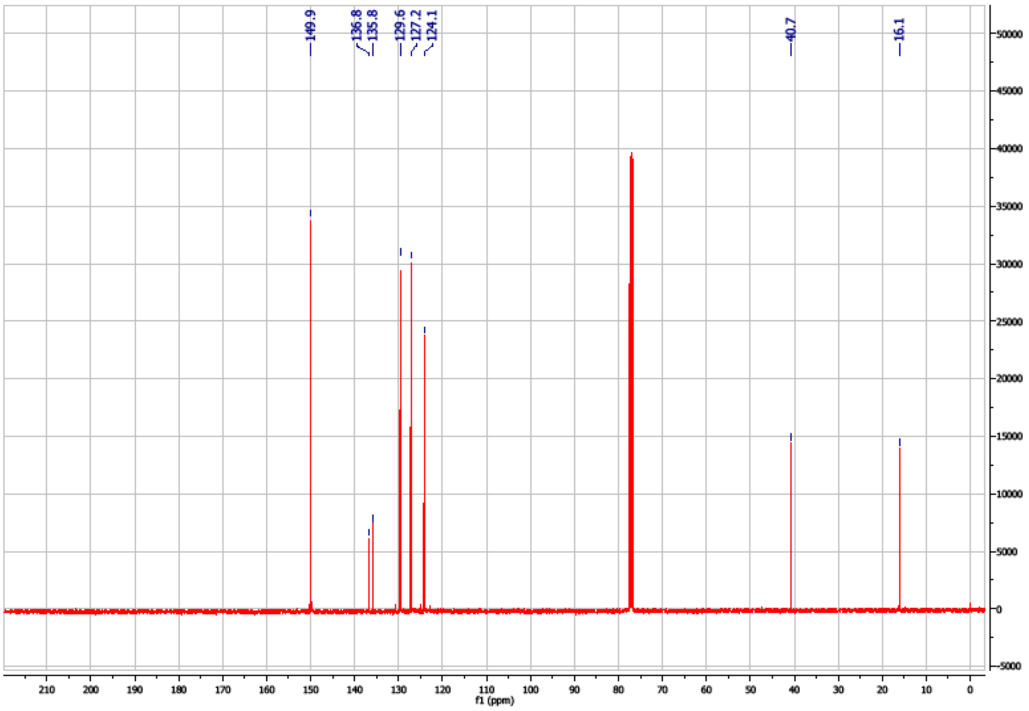

4-(4-Methoxybenzyl)pyridine

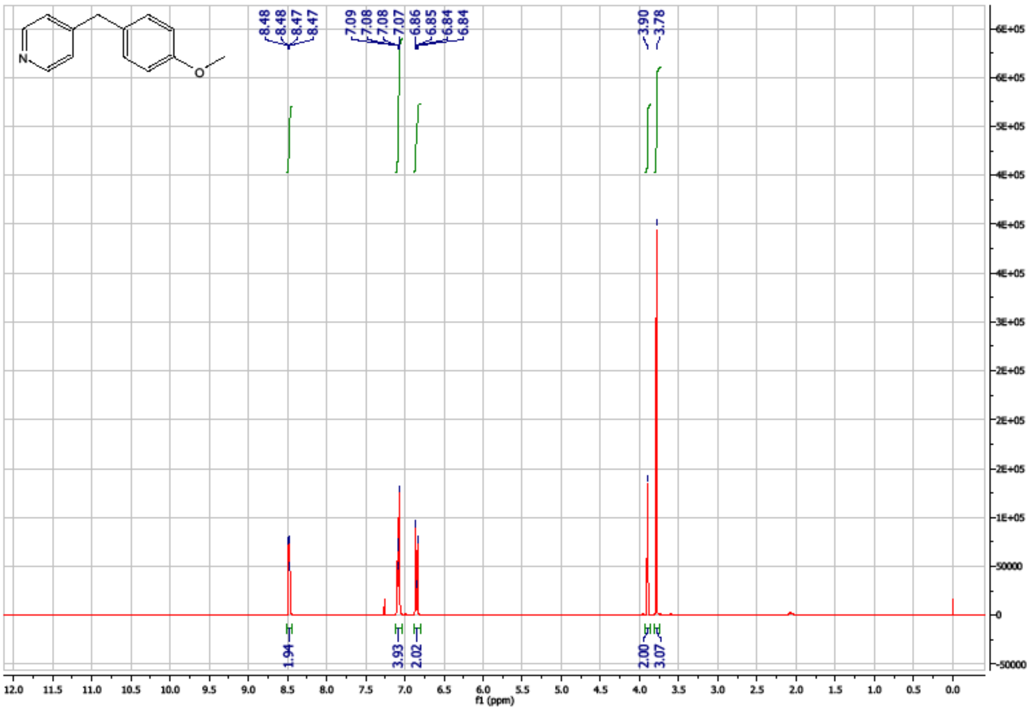


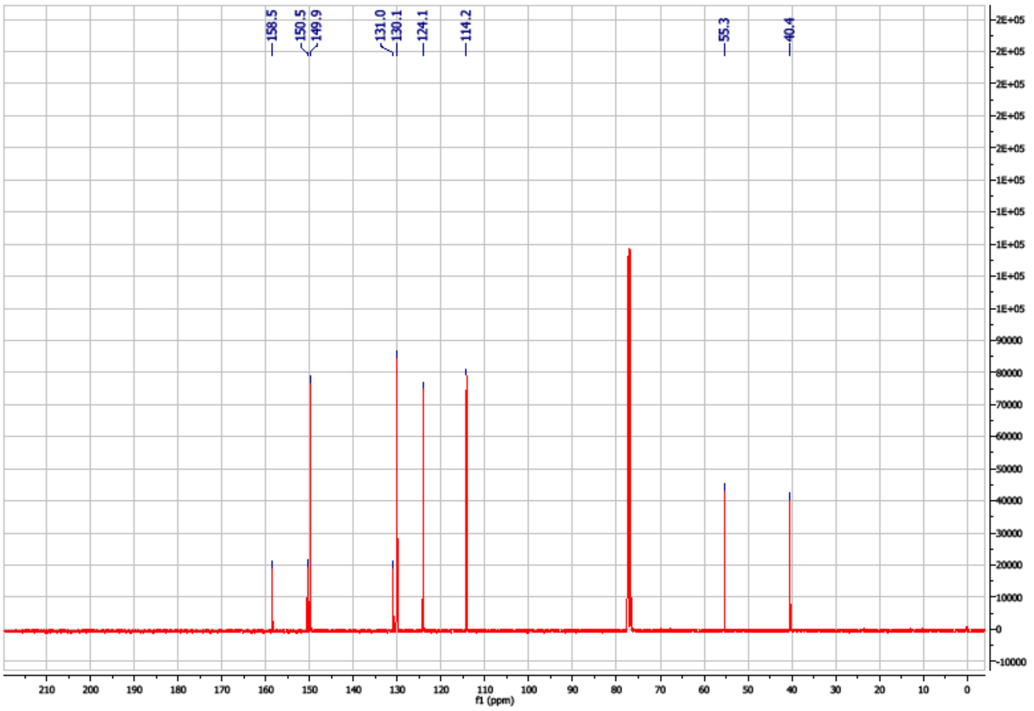

4-(4-Methylbenzyl)pyridine

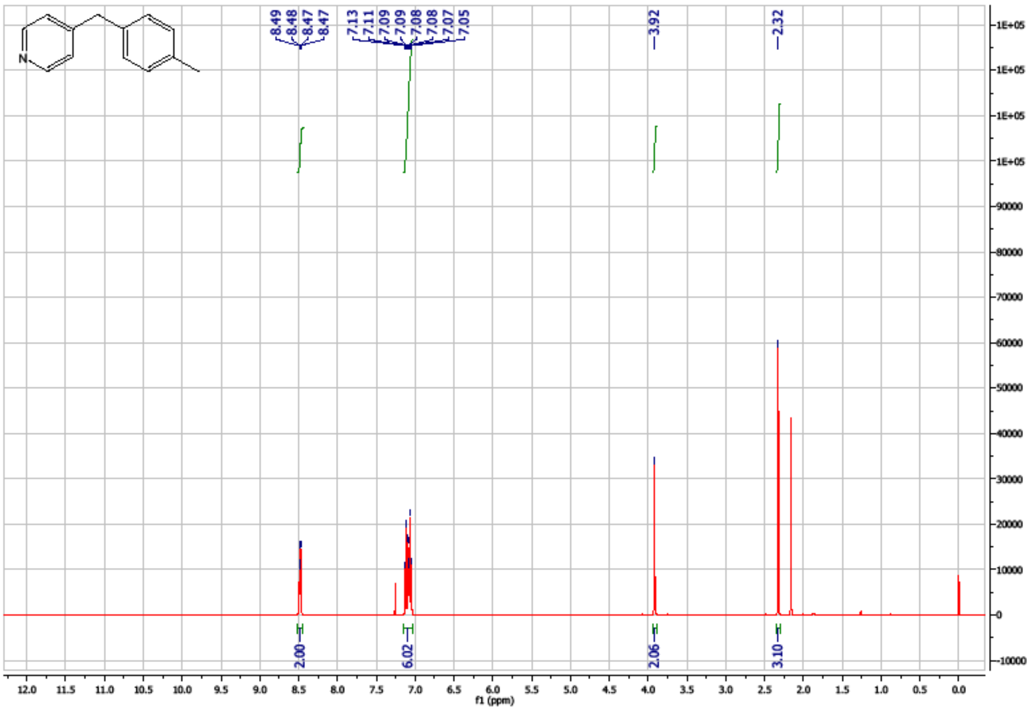


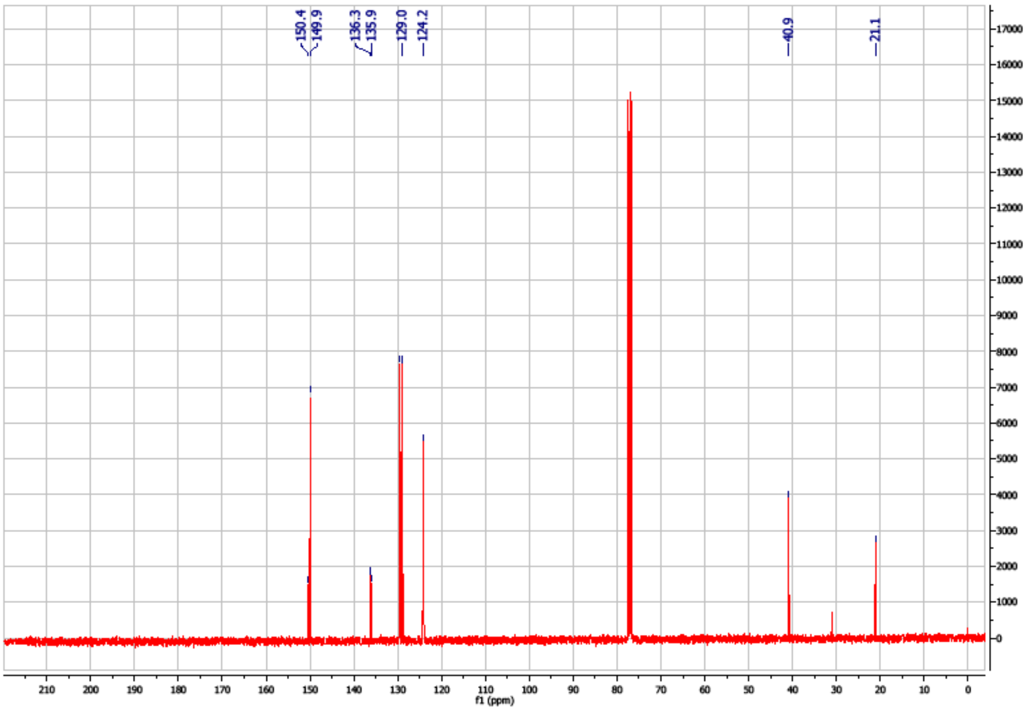

4-(4-Fluorobenzyl)pyridine

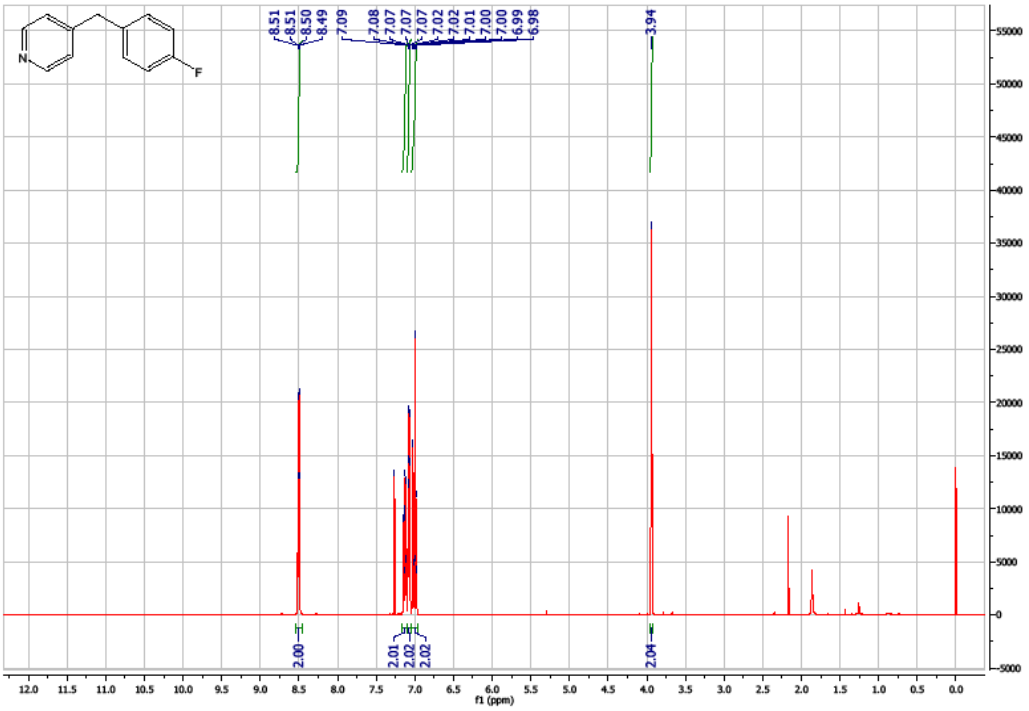

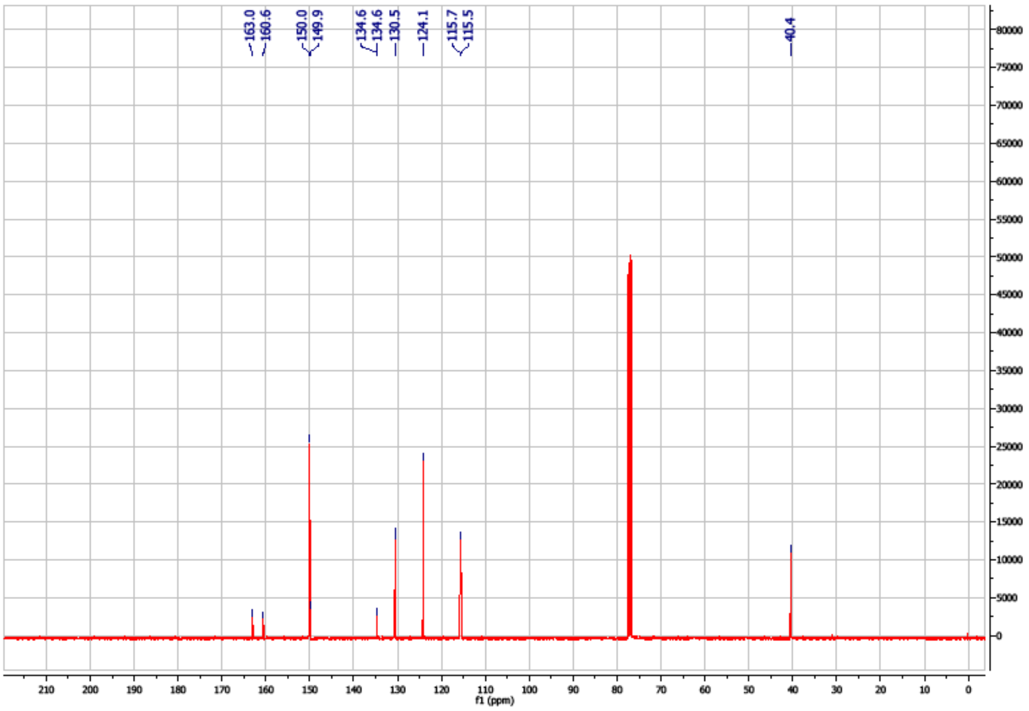

4-(4-Chlorobenzyl)pyridine

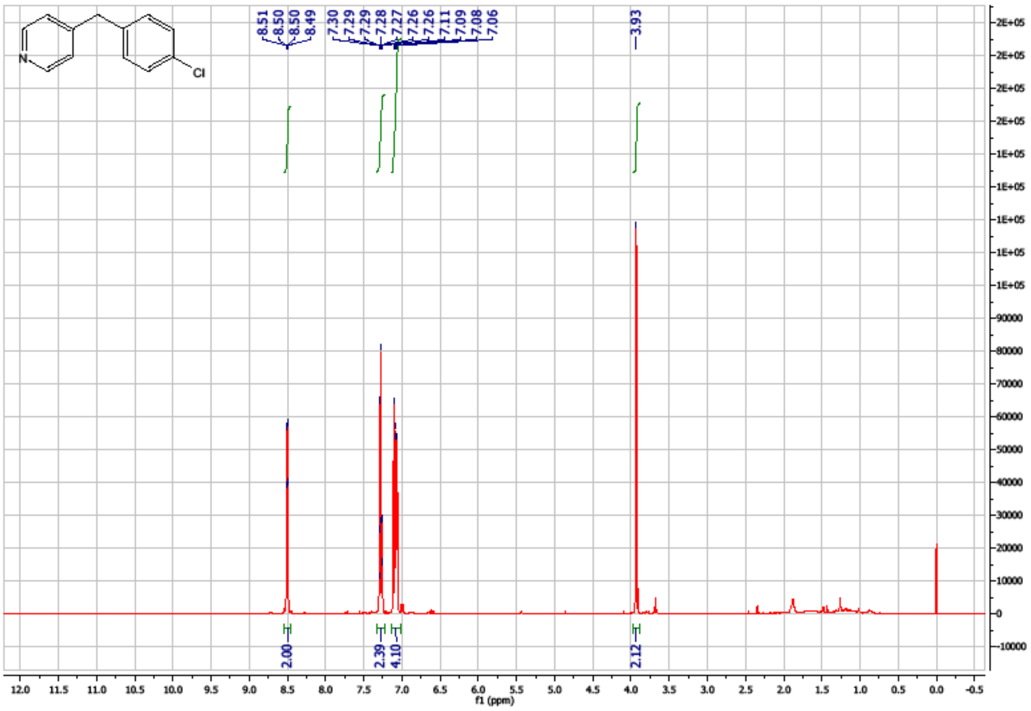


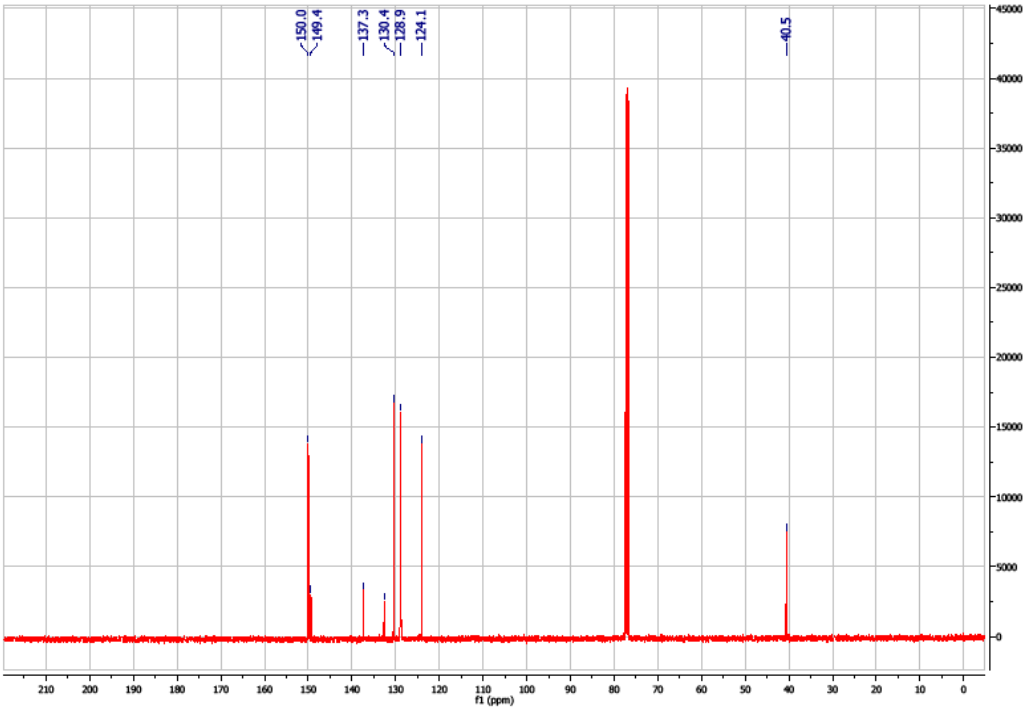

Ethyl 4-(pyridin-4-ylmethyl)benzoate

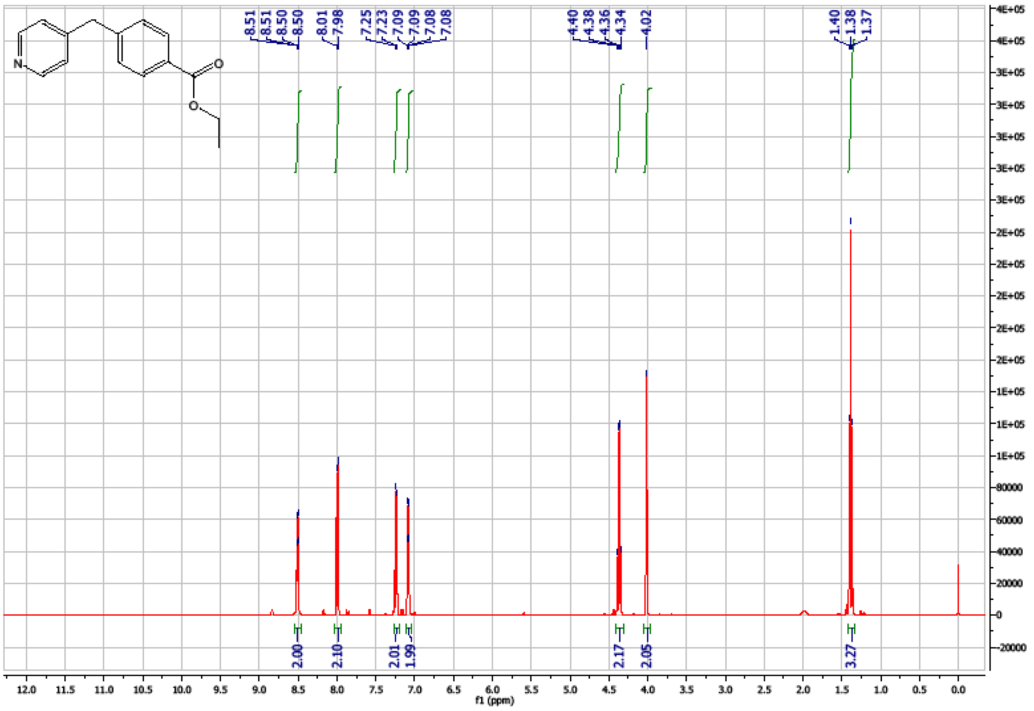


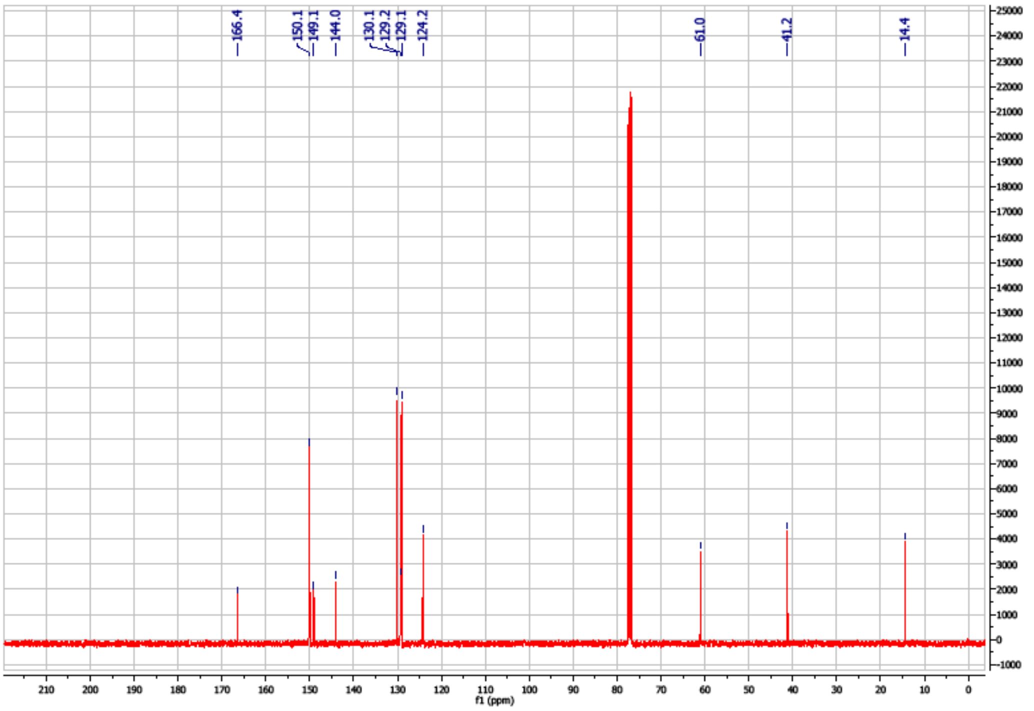

4-(Pyridin-4-ylmethyl)benzonitrile

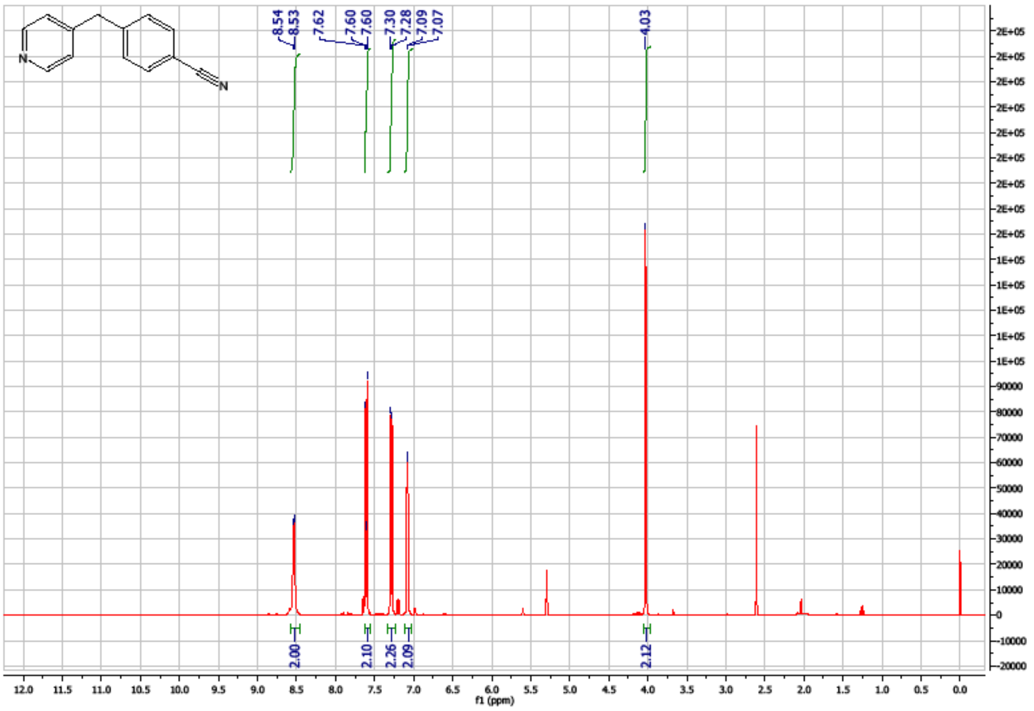

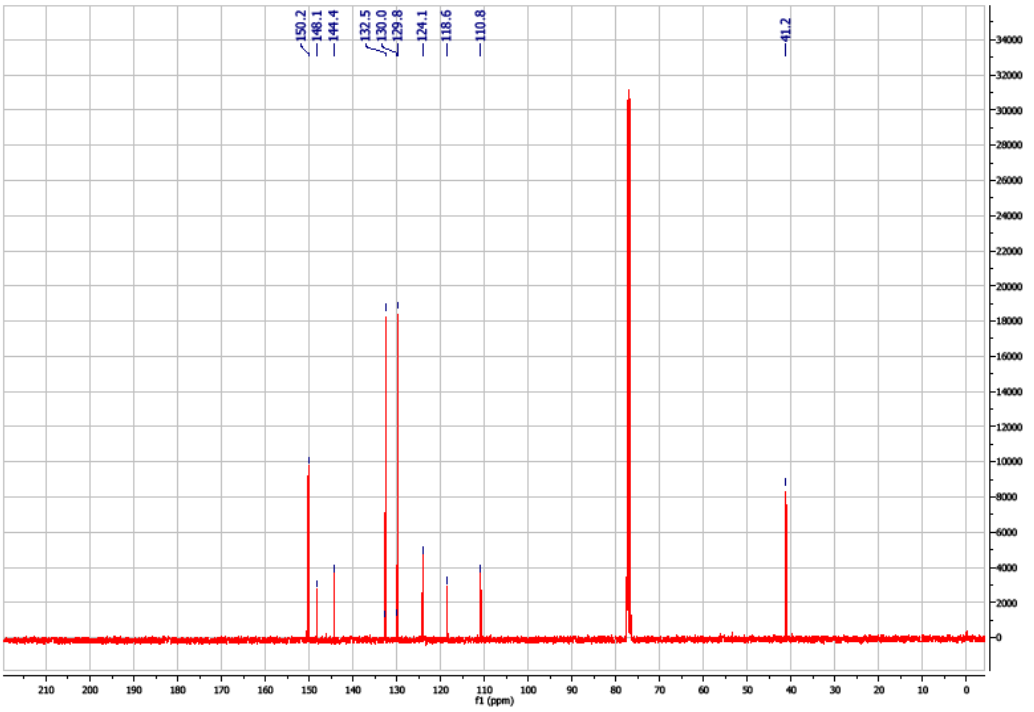

*N*-(6-Benzylpyridin-3-yl)acetamide

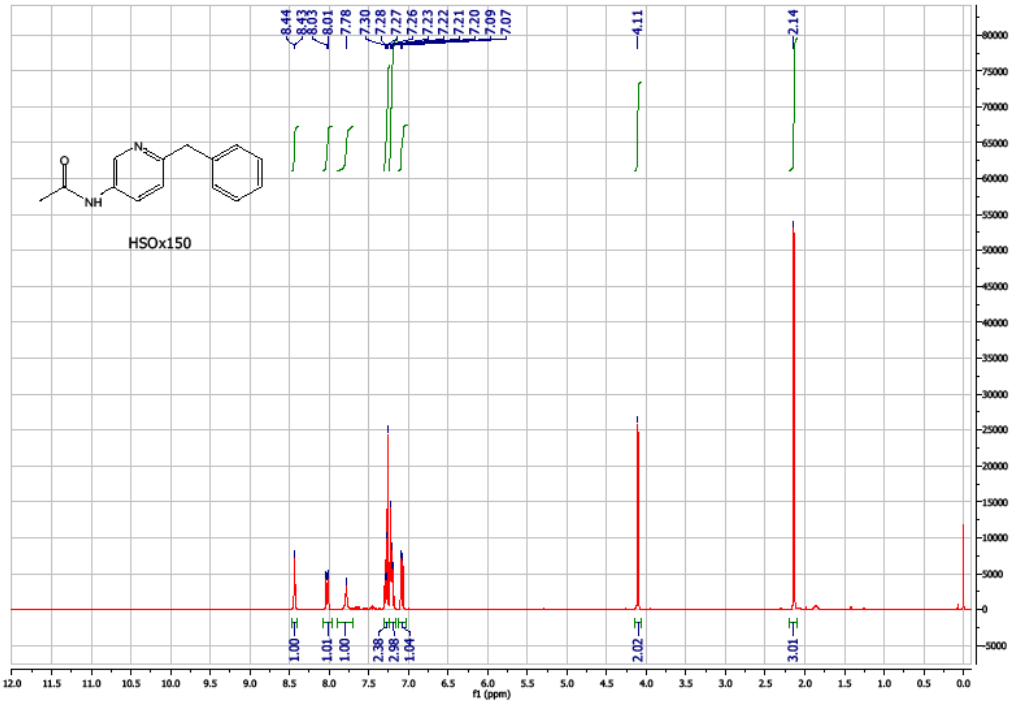


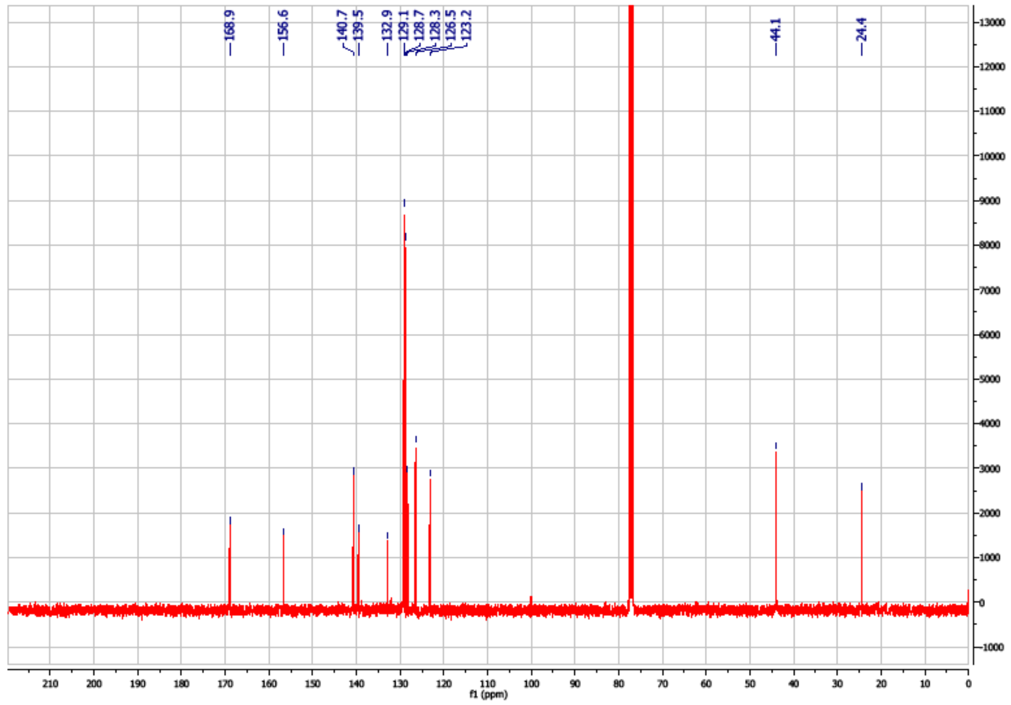

4-Benzyl-2-methylpyrimidine

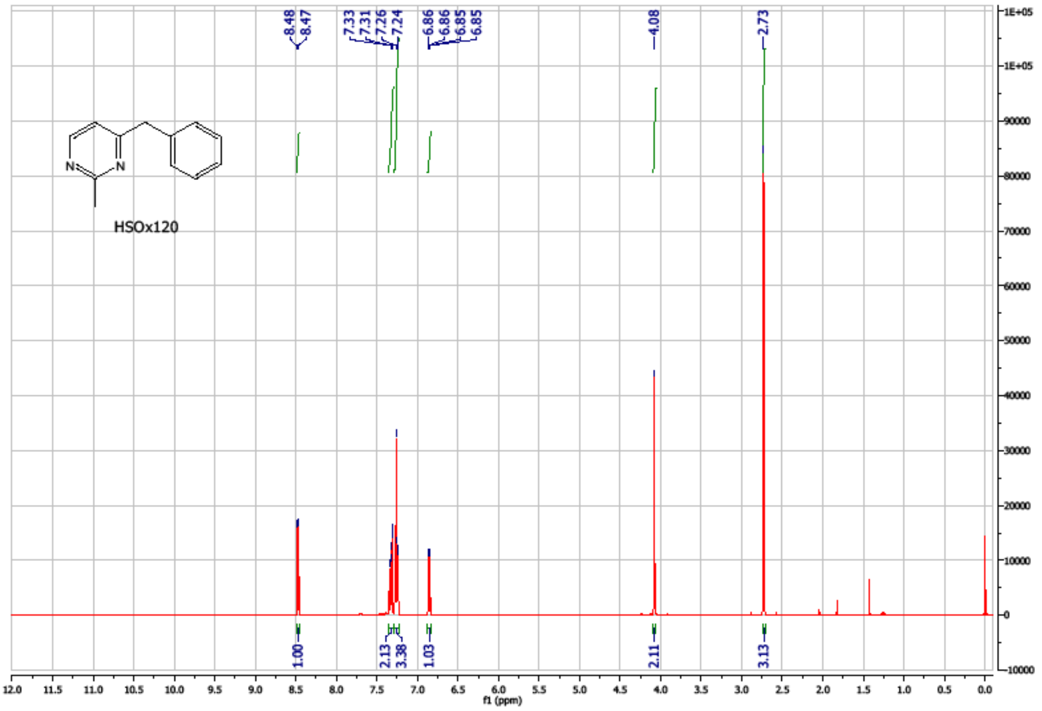


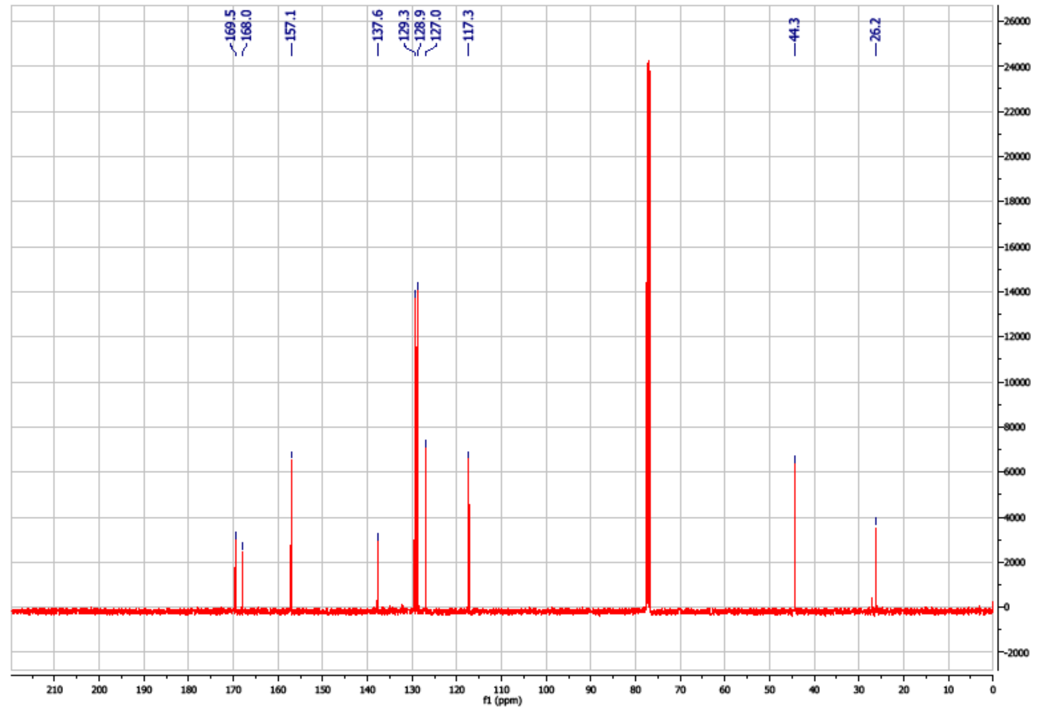

3-Benzyl-6-methylpyridazine

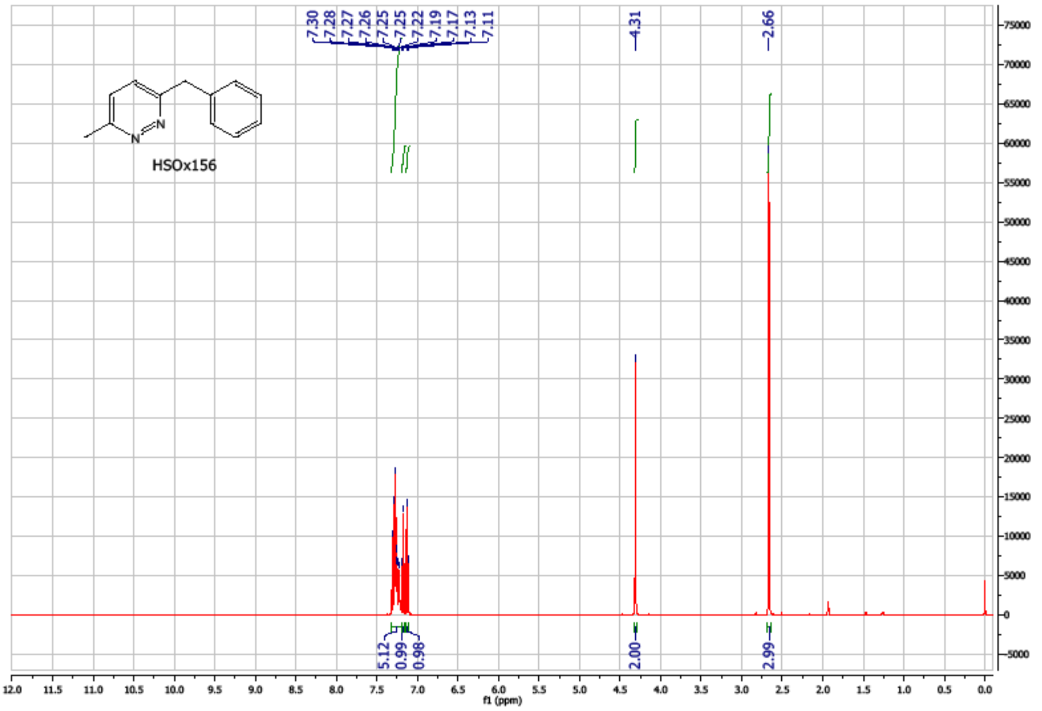


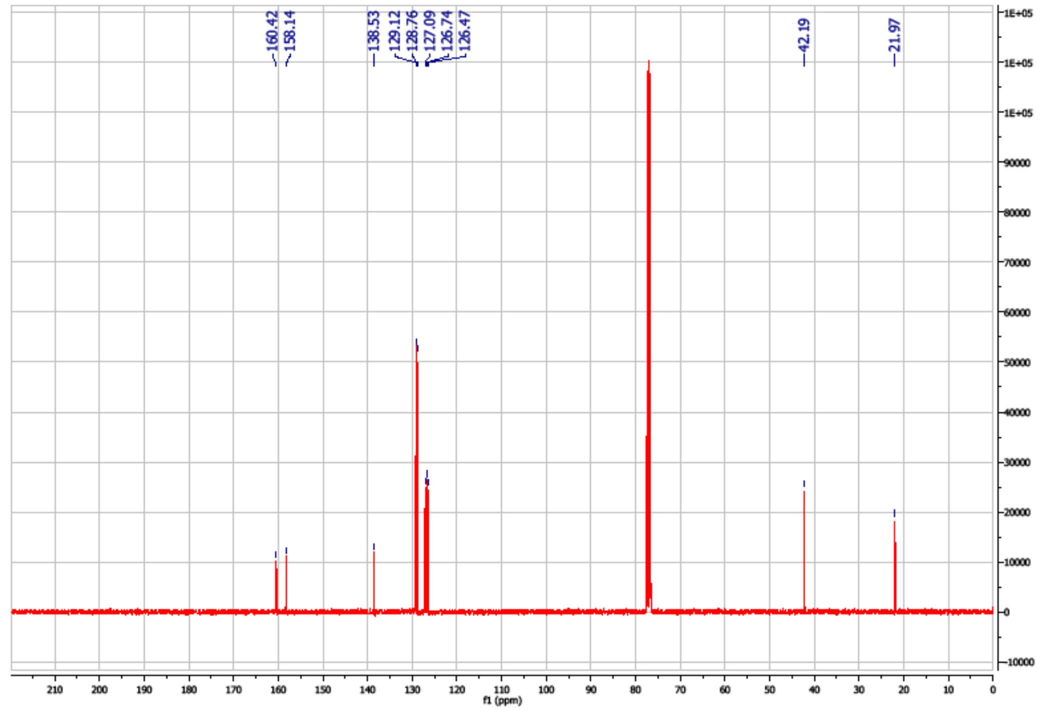

4-(4-Iodobenzyl)pyridine

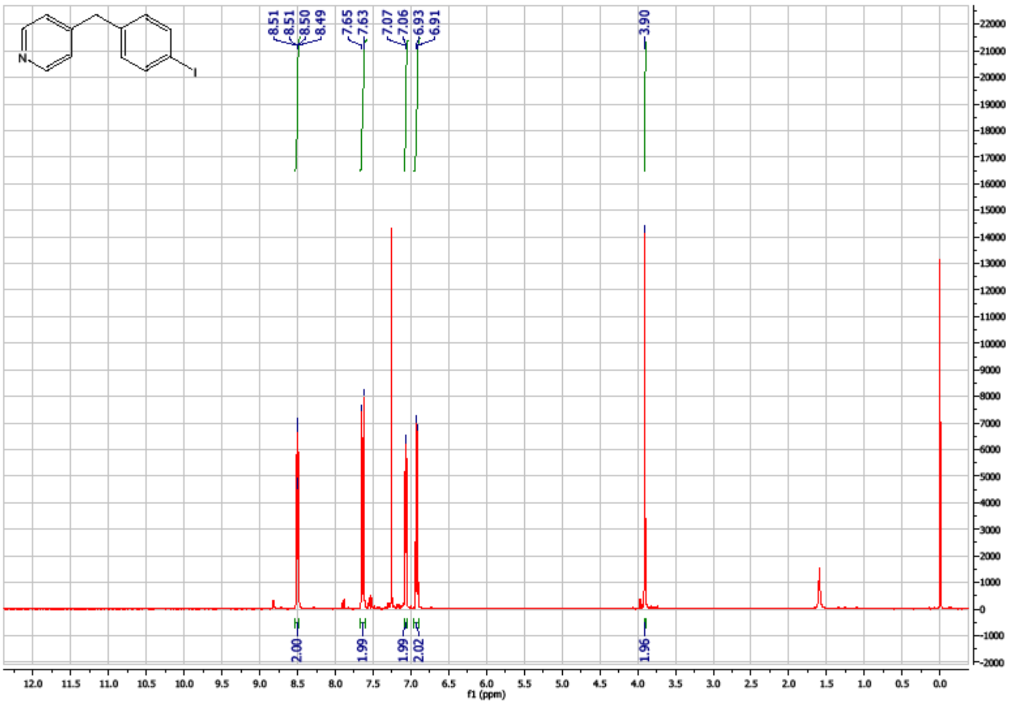

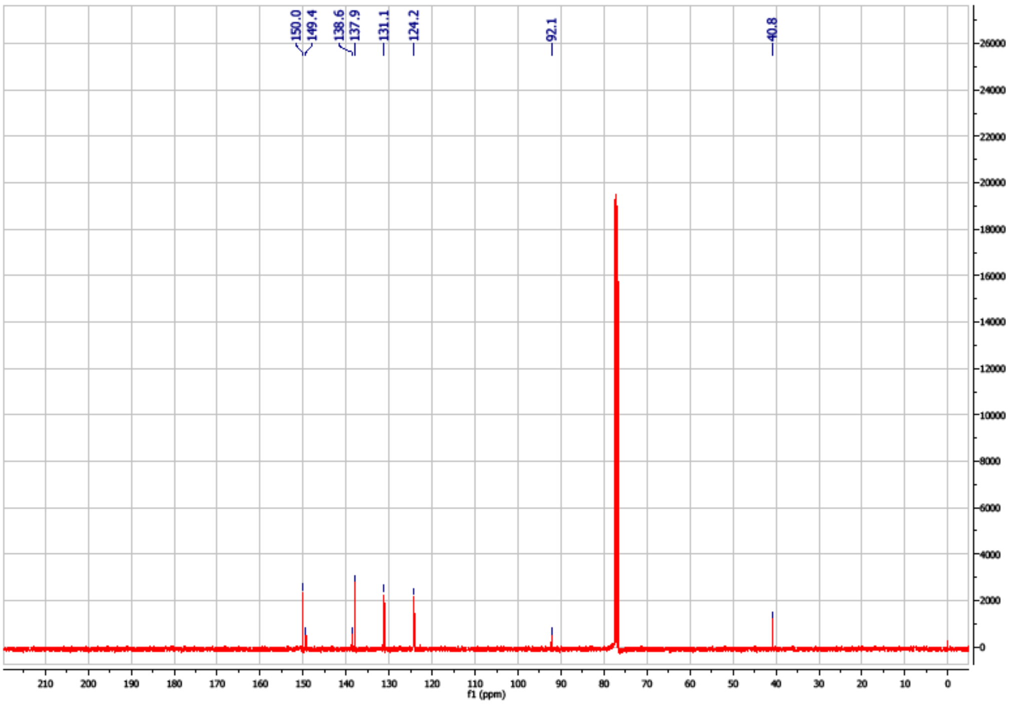

4-(4-Bromobenzyl)pyridine

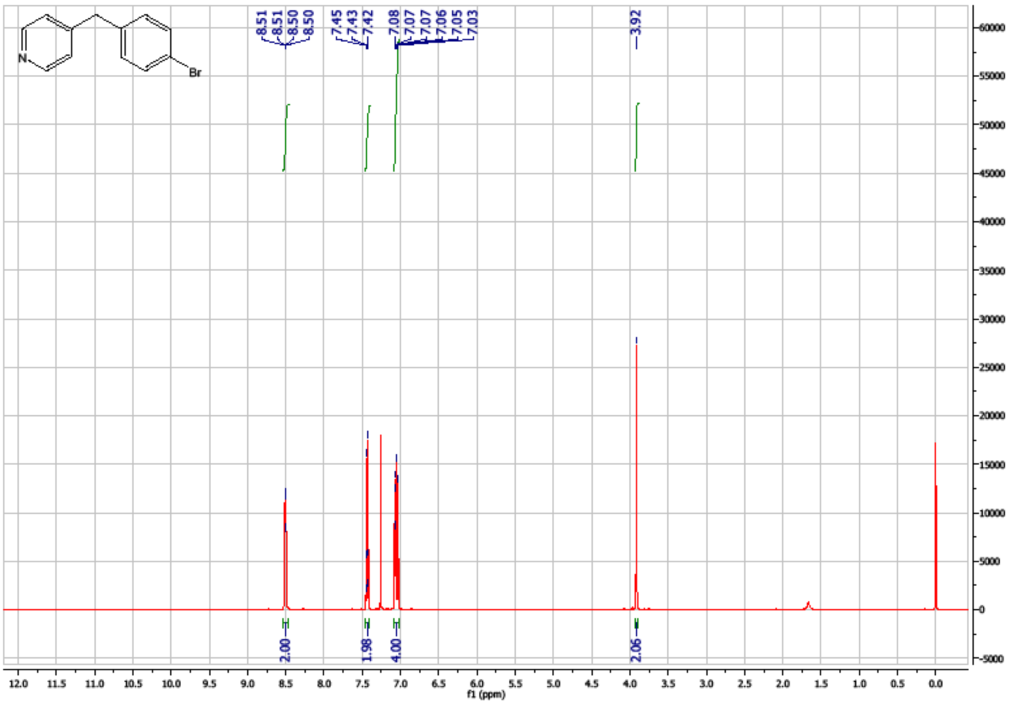


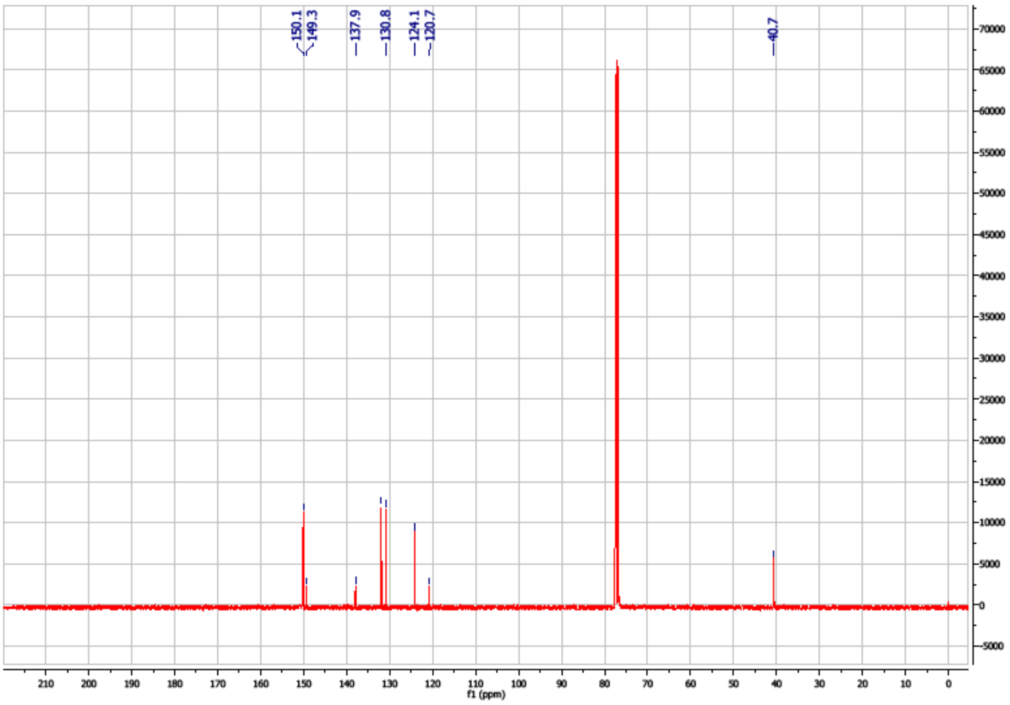

4-(Pyridin-4-ylmethyl)aniline

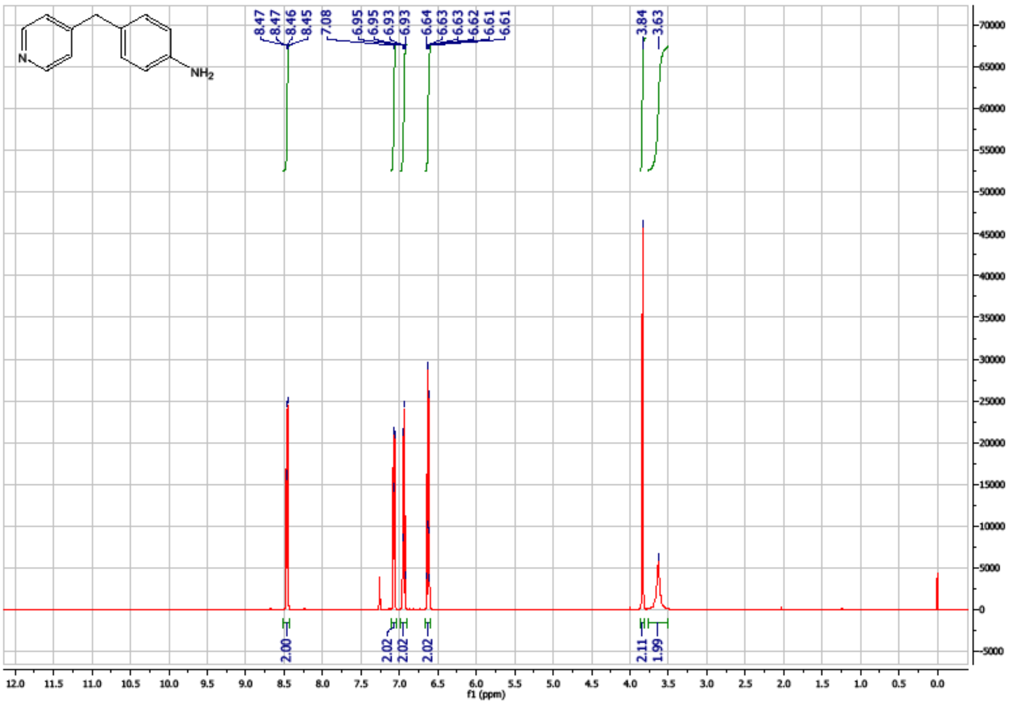


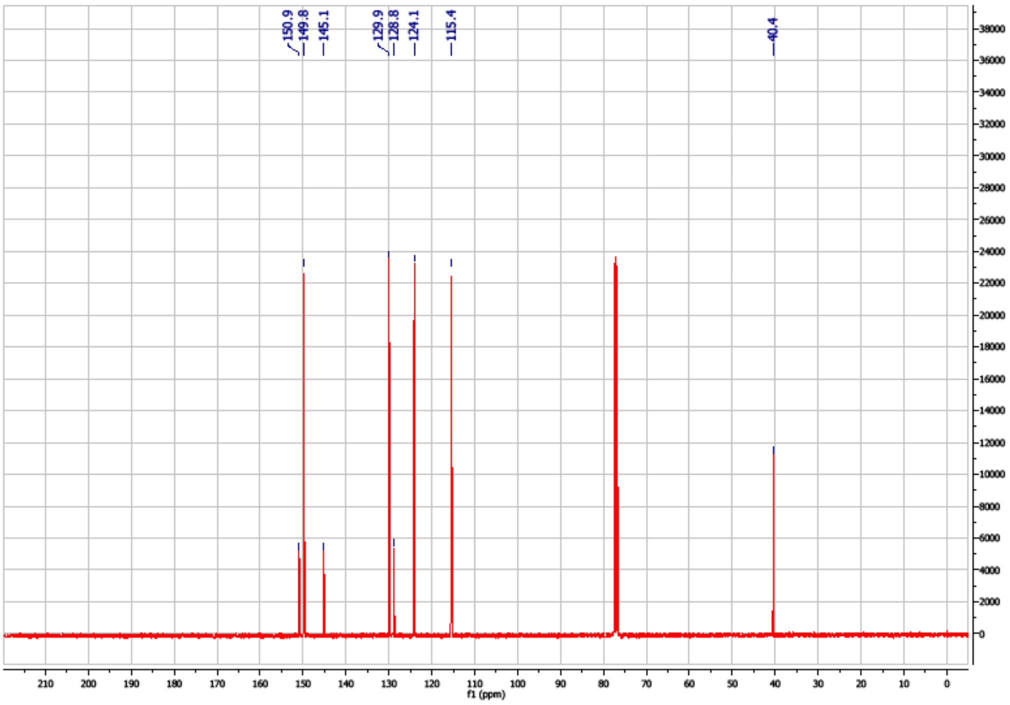

2-Benzylquinoline

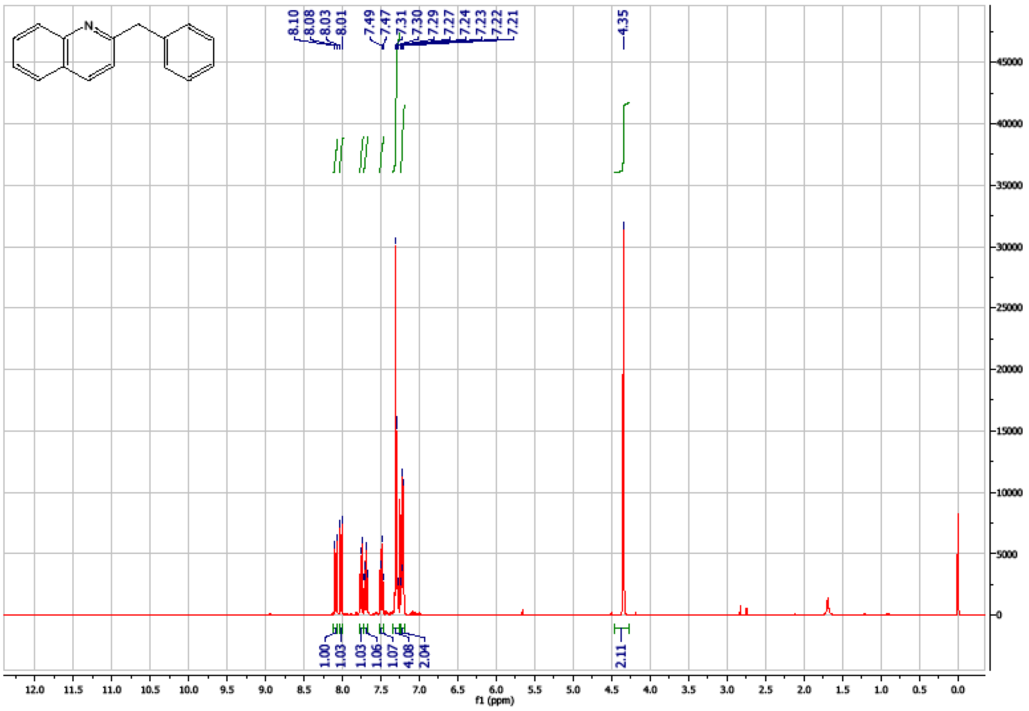


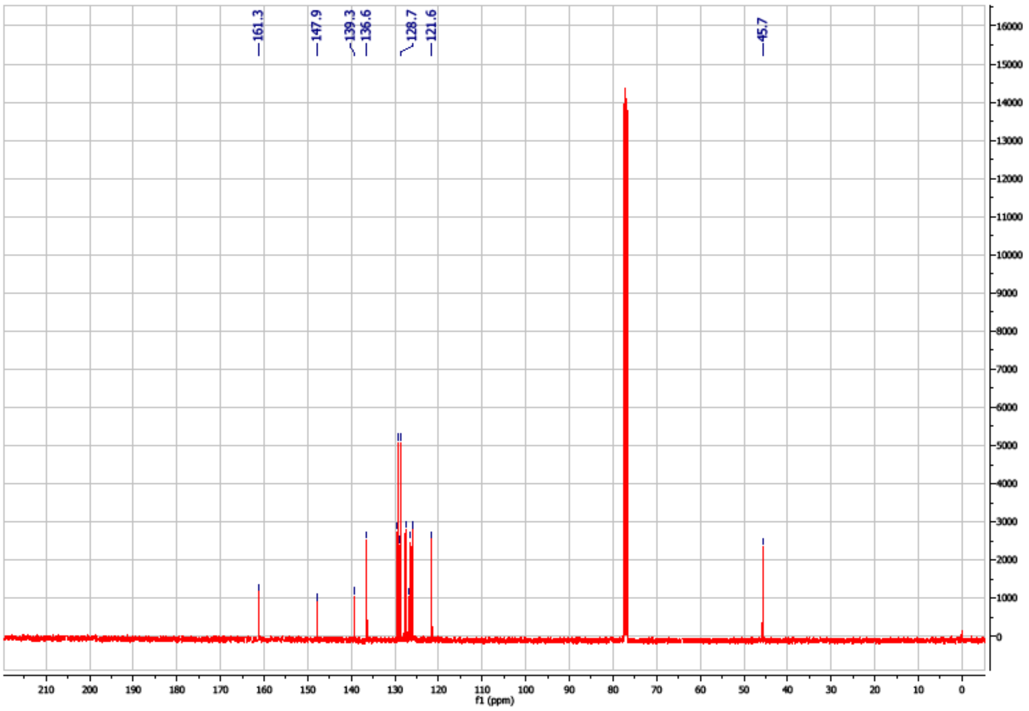

4-Benzylquinoline

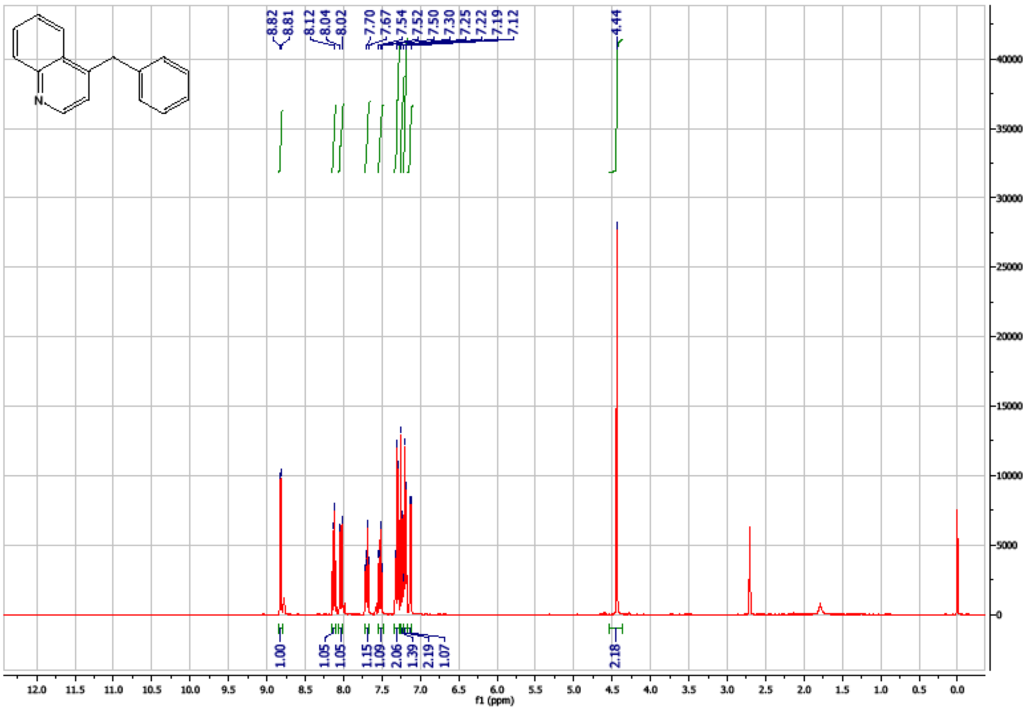

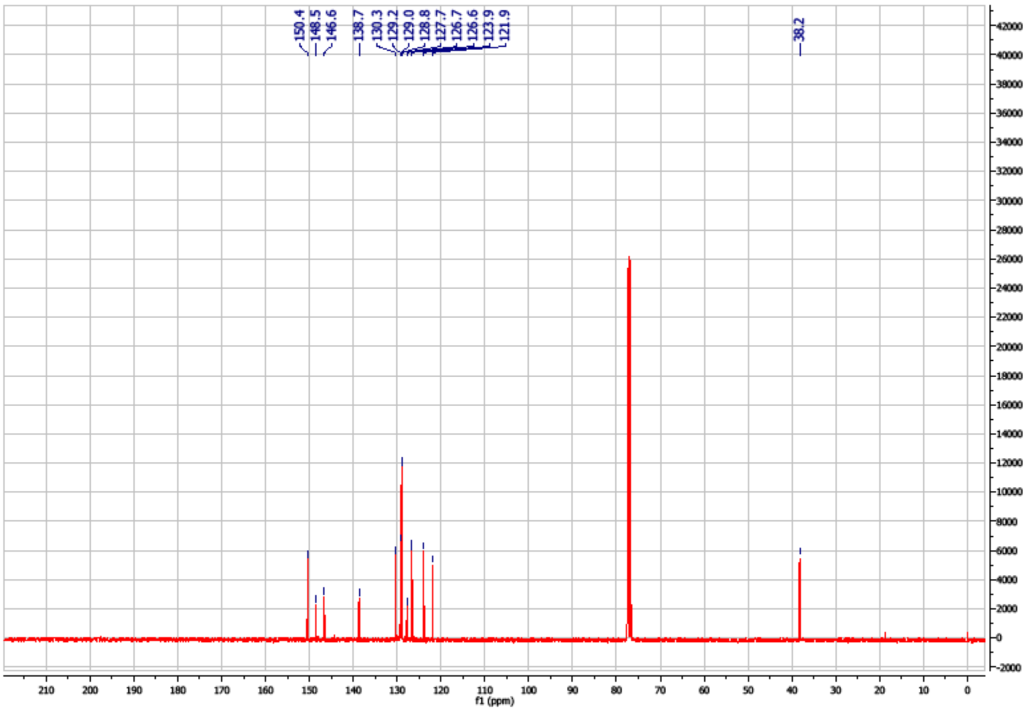

4-(Pyridin-2-ylmethyl)-2,8-bis(trifluoromethyl)quinoline

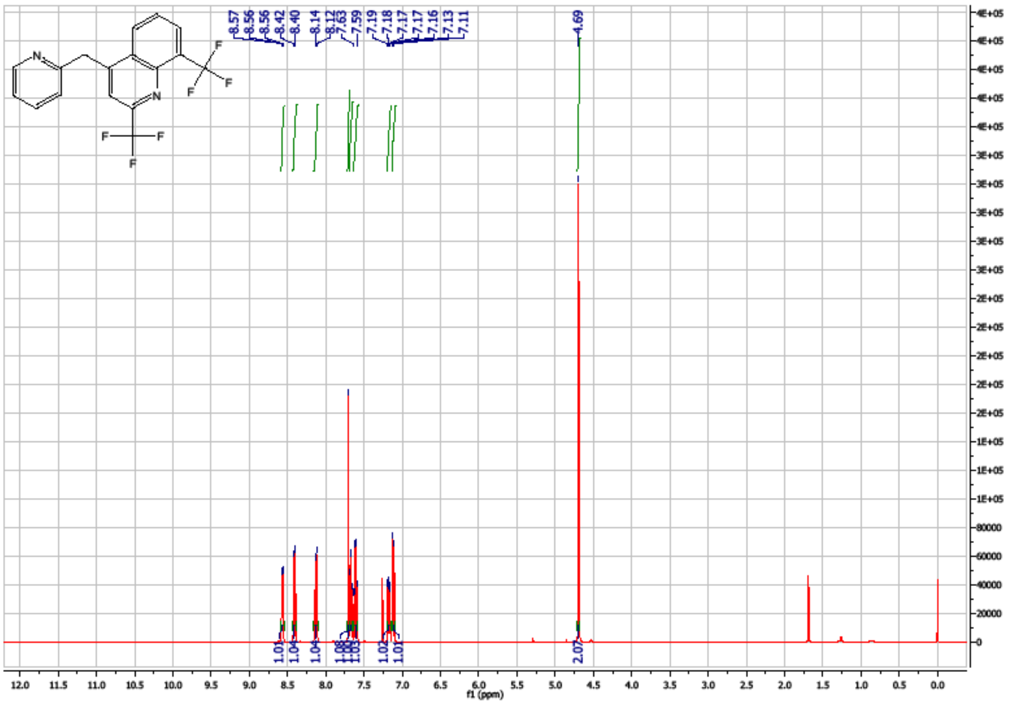

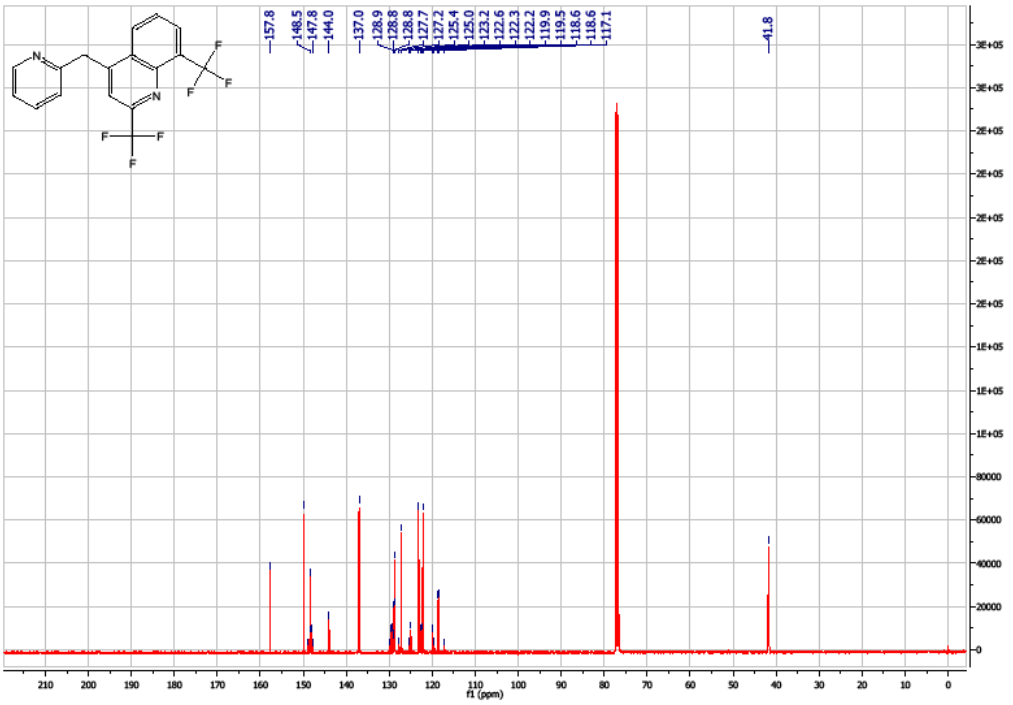

2-(4-Chlorobenzyl)pyrimidine

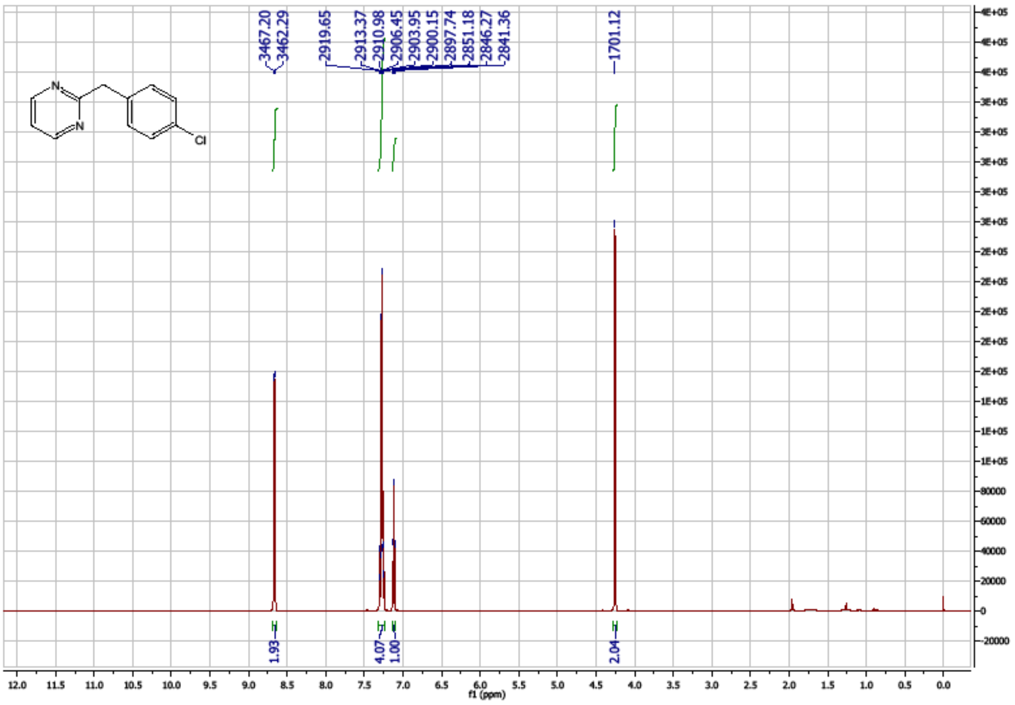


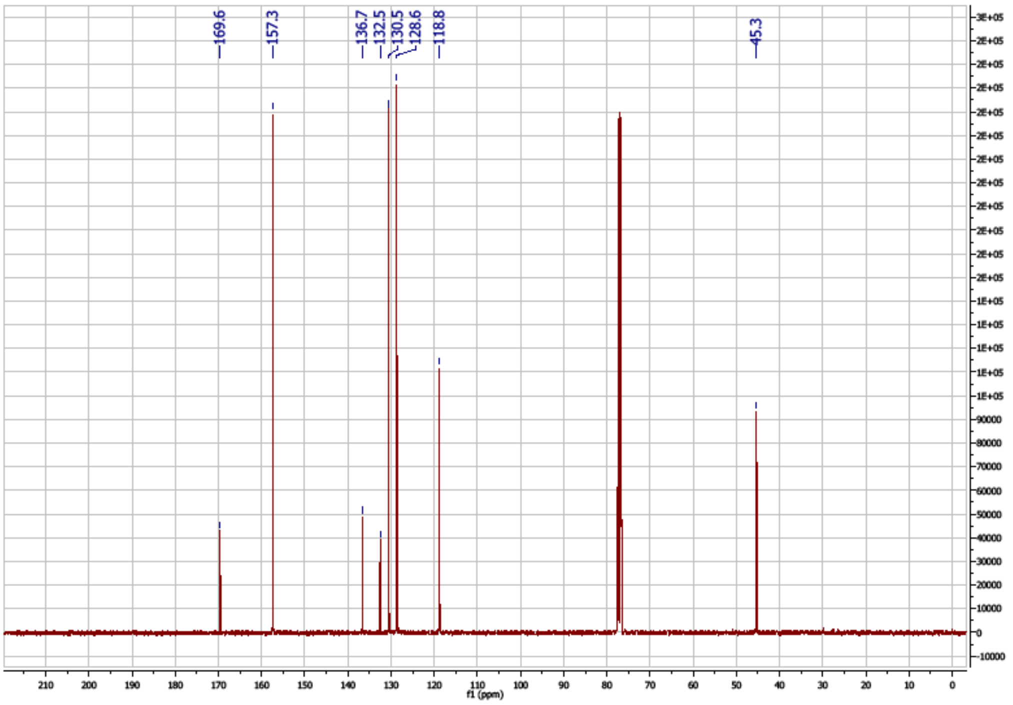

2-(4-Chlorobenzyl)pyrazine

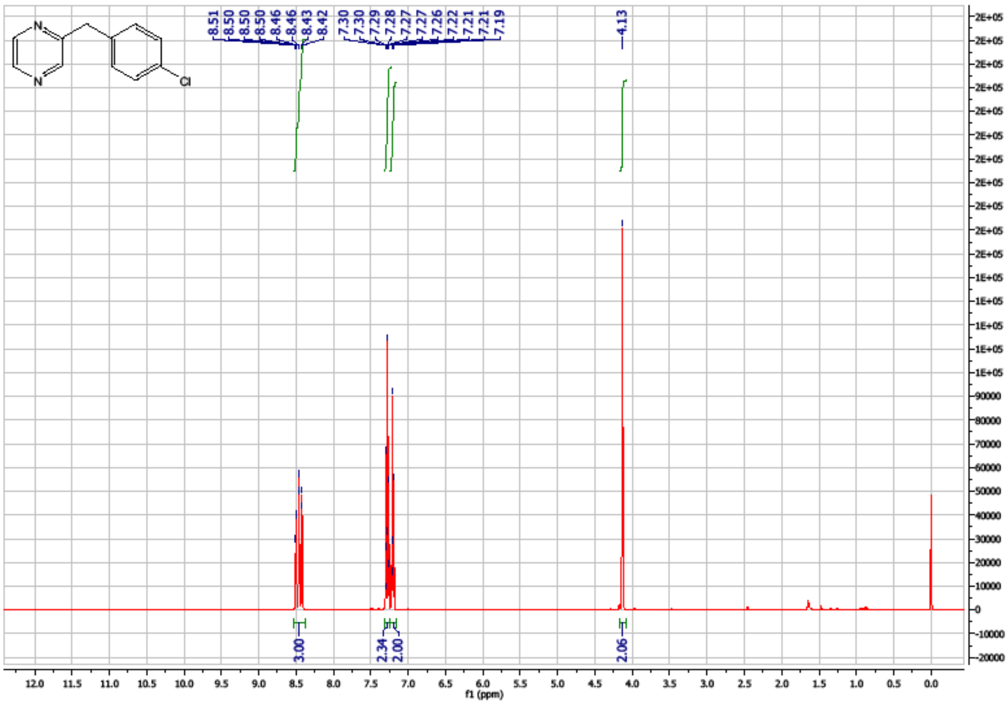


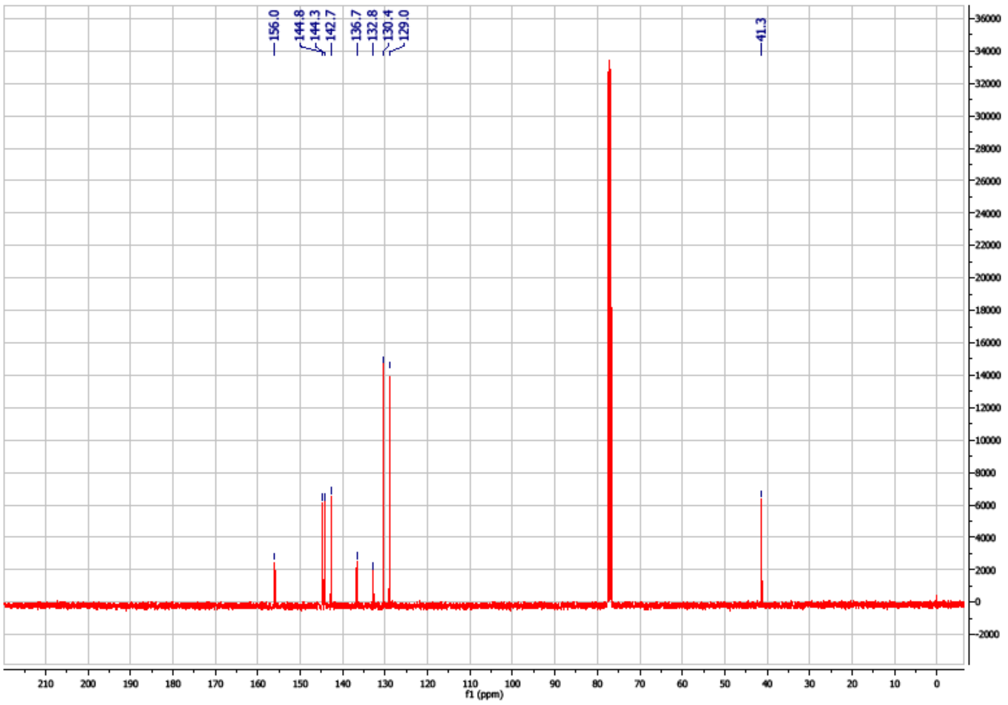

6-(4-Methylbenzyl)-2-methylpyrazine

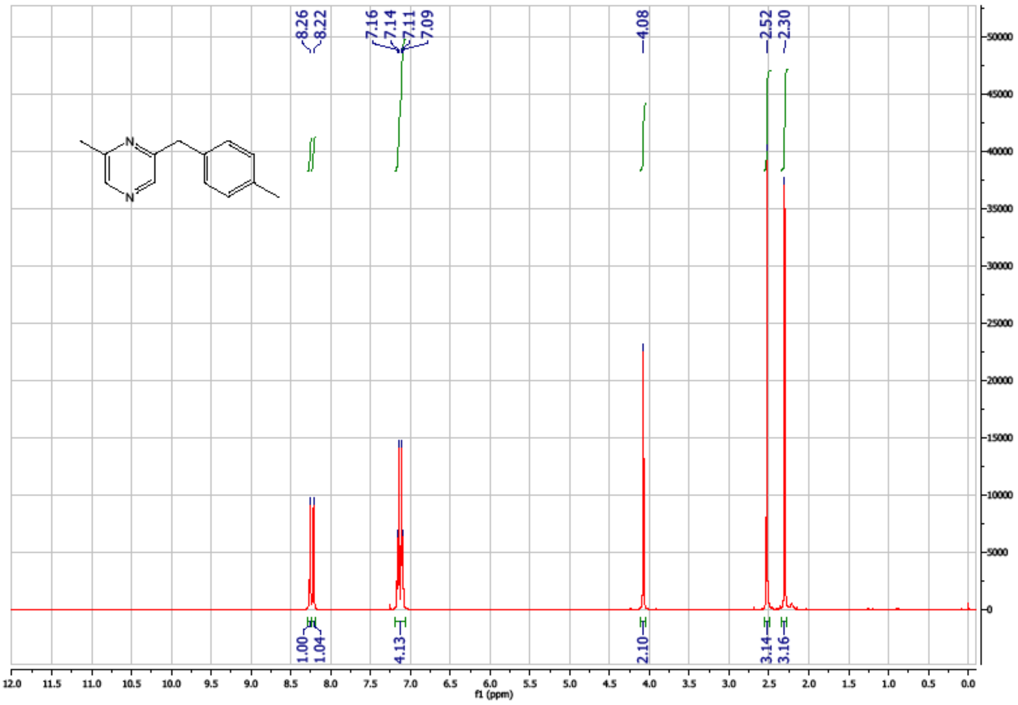


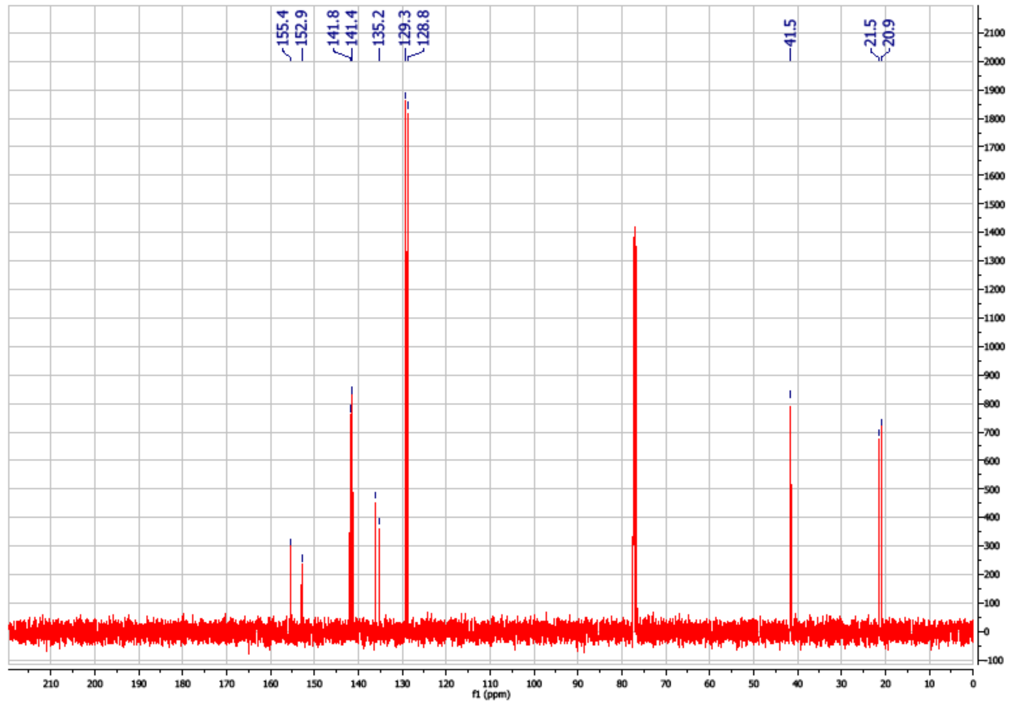

Supplement: File 1 — Experimental procedures, compound characterization data and copies of 1H and 13C NMR spectra of all new starting materials and reaction products. [file Beilstein_J_Org_Chem-12-144-s001.pdf]
